# Impact of catch-up human papillomavirus vaccination on cervical cancer incidence in Kenya: A mathematical modeling evaluation of HPV vaccination strategies in the context of moderate HIV prevalence

**DOI:** 10.1016/j.eclinm.2022.101306

**Published:** 2022-02-19

**Authors:** Gui Liu, Nelly R Mugo, Cara Bayer, Darcy White Rao, Maricianah Onono, Nyaradzo M Mgodi, Zvavahera M Chirenje, Betty W Njoroge, Nicholas Tan, Elizabeth A Bukusi, Ruanne V Barnabas

**Affiliations:** a Department of Global Health, University of Washington, Seattle, USA; b Kenya Medical Research Institute, Nairobi, Kenya; c Department of Epidemiology, University of Washington, Seattle, USA; d University of Zimbabwe Clinical Trials Research Centre, Harare, Zimbabwe; e Faculty of Medicine and Health Sciences, University of Zimbabwe, Harare, Zimbabwe; f Creighton University School of Medicine, Phoenix, USA; g Department of Obstetrics and Gynecology, University of Washington, Seattle, USA; h Vaccine and Infectious Disease Division, Fred Hutchinson Cancer Research Center, Seattle, USA

**Keywords:** HPV vaccination, HIV, Cervical cancer, Mathematical model, HPV

## Abstract

**Background:**

Cervical cancer incidence is high in Kenya due to HIV and limited access to cancer prevention services. Human papillomavirus (HPV) has been shown to increase HIV acquisition; however, the potential impact of HPV vaccination on HIV is unknown. We modeled the health impact of HPV vaccination in the context of the HIV epidemiology in Kenya.

**Methods:**

Using a validated compartmental transmission model of HIV and HPV set in Kenya, we evaluated five scenarios of nonavalent HPV vaccination: single-age-vaccination of 10-year-old girls at 90% coverage; multi-age-cohort (MAC) vaccination of 10-14-year-old girls at 90% coverage; MAC plus moderate-coverage (50%) catch-up vaccination of 15-24-year-old women; MAC plus high-coverage (80%) catch-up of 15-24-year-old women; and MAC plus catch-up of 15-44-year-old women at 80% coverage (HPV-FASTER). We compared cervical cancer incidence, HIV prevalence, and cumulative cervical cancer and HIV cases averted after 50 years to a baseline scenario without vaccination. In all scenarios, we assumed the UNAIDS 90-90-90 goal for HIV treatment is attained by 2030.

**Findings:**

In 2021, model-estimated cervical cancer incidence is 44/100,000 and HIV prevalence among women is 6·5%. In 2070, projected cancer incidence declines to 27/100,000 and HIV prevalence reaches 0·3% without vaccination. With single-age-vaccination, cancer incidence in 2070 is reduced by 68%, averting 64,529 cumulative cancer cases. MAC vaccination reduces cancer incidence by 75%, averting 206,115 cancer cases. Moderate and high-coverage catch-up and HPV-FASTER reduce cancer incidence by 80%, 82%, and 84%, averting 254,930, 278,690, and 326,968 cancer cases, respectively. In all scenarios, HIV prevalence in 2070 is reduced by a relative 8-11%, with 15,609-34,981 HIV cases averted after 50 years.

**Interpretation:**

HPV vaccination can substantially reduce cervical cancer incidence in Kenya in the next 50 years, particularly if women up to age 24 are vaccinated. HIV treatment scale-up can also alleviate cervical cancer burden. However, HPV vaccination has modest additional impact on HIV when antiretroviral therapy coverage is high.

**Funding:**

National Institutes of Health, Bill and Melinda Gates Foundation

**Research in context**

**Evidence before this study**

Cervical cancer is the leading cancer among women in Kenya, due to the high burden of HIV and low coverage of vaccination against human papillomavirus (HPV) infection and other cervical cancer prevention services. We searched PubMed on September 5, 2021 for modeling studies evaluating HPV vaccination impact in Kenya without language or date restrictions using the terms: (“model* OR “simulation”) AND (“HPV” OR “human papillomavirus”) AND (“vaccin*”) AND (“Kenya”). None of the resulting studies evaluated high-coverage vaccination strategies, such as catch-up vaccination for older adolescents and young women or accounted for the impact of HIV on cervical cancer. In addition, modifying our search to remove restrictions to Kenya and adding the terms: (“HIV incidence” OR “HIV acquisition” OR “HIV risk” OR “HIV prevalence”), we found no studies evaluating the impact of HPV vaccination on HIV burden.

**Added value of this study**

Our results suggest that HPV vaccination strategies that include young and mid-adult women have the largest impact in reducing cervical cancer burden. In addition, HIV prevention interventions can reduce cervical cancer incidence in the absence of HPV vaccination. Although HPV vaccination has a modest impact on HIV burden in Kenya, vaccination may have a larger impact in settings with higher HIV incidence.

**Implications of all the available evidence**

Our findings support the widespread implementation of HPV vaccination to prevent cervical cancer. In addition to a high-coverage routine vaccination program, temporarily providing catch-up vaccination for older adolescents and young women can lead to significant reductions in cervical cancer incidence in a country with limited access to cervical cancer screening and treatment.

Alt-text: Unlabelled box

## Introduction

With advances in primary and secondary cervical cancer prevention in high-income countries (HICs), cervical cancer is increasingly an indicator of global health disparities. Approximately 90% of the estimated 604,000 new cervical cancer cases and 342,000 deaths due to cervical cancer in 2020 occurred in low- and middle-income countries (LMIC).^1^ Due to limited access to cervical cancer screening and treatment, the cervical cancer incidence rate is more than 5 times higher in Kenya compared to HICs.^1^^,^^2^ Inequitable access to vaccines against human papillomavirus (HPV) infection is likely to exacerbate cervical cancer disparities in the coming decades.

Cervical cancer is one of the most common cancers and cause of cancer deaths among Kenyan women.^1^ HIV infection among women is an important contributing factor to the high burden of cervical cancer in Kenya.^3^ Women with HIV have significantly higher risk of HPV infection and a six-fold higher risk of cervical cancer compared to women without HIV.^4^ As of 2020, HIV prevalence among Kenyan adult women was 6%; representing almost 900,000 women with HIV who are at elevated risk for cervical cancer compared to women without HIV.^5^ In addition, a meta-analysis of 11 studies found that HPV infection is associated with a two-fold increase in HIV acquisition, suggesting that preventing HPV infection could potentially reduce HIV burden.^4^

HPV vaccines are safe, durable, and highly efficacious in preventing infection with several oncogenic HPV types and the sequelae associated with these infections.^6^^,^^7^ Further, vaccination has been shown to be a cost-effective cervical cancer prevention strategy across a wide range of countries, including Kenya, even after accounting for opportunity cost to other health services.^8^ To accelerate HPV vaccination impact at the population level, the World Health Organization (WHO) recommends multi-age-cohort vaccination of girls 9-15 years old.^9^ However, Kenya and many other LMICs are vaccinating a single age cohort (girls aged 9 or 10) due to limited vaccine supply and funding, resulting missed opportunities for primary prevention in older cohorts.^10^ Encouragingly, investment priorities of local ministries of health and international donors may shift following the passage of the resolution to eliminate cervical cancer as a public health problem globally at the 2020 World Health Assembly.^11^ The resolution was adopted with support from 194 member countries.^11^

A crucial component to the success of the cervical cancer elimination initiative is increasing HPV vaccination coverage in LMICs.^11^ Vaccine implementation will need to be tailored to each country; mathematical models have estimated that the population-level impact of vaccination on cervical cancer rates will vary depending on the ages targeted for vaccination and the baseline level of cervical cancer risk.^12^ In addition, in high HIV burden countries, prevention of HIV could independently reduce cervical cancer incidence and mortality.^13^ In this modeling study, we estimated the population-level impact of different HPV vaccination strategies on cervical cancer burden in Kenya, while accounting for the effects of HIV prevention scale-up and changing HIV epidemiology. As a secondary objective, we evaluated the impact of HPV vaccination on HIV burden, as our model captures the increased risk of HIV acquisition among women with an HPV infection.^4^ Estimates from our study could inform public health decision-making in Kenya and other countries with similar levels of cervical cancer and HIV.

## Methods

### Model description

We adapted a previously published compartmental, dynamic model of heterosexual HPV and HIV transmission to the Kenyan setting.^14^ The model simulated demographic dynamics, HIV infection and progression, HPV infection and progression to cervical cancer, and the interactions between HIV and HPV infections. Births and background mortality over time were parameterized using Kenya-specific fertility and non-HIV mortality rates from United Nations Population Division (Tables S2 and S4).^15^ We modelled males and females aged 0-79 in five-year age groups with age- and sex-specific sexual behaviors.

Susceptible individuals in the model acquired HIV through heterosexual contact or, to a lesser extent, through mother-to-child transmission. We assumed women with an HPV infection had higher probability of HIV acquisition compared to those without HPV infection (relative risk of 1.7, 95% CI 1.4 to 2.2).^4^ HIV progression was characterized by decreasing CD4 cell count and increasing HIV viral load over time. People with HIV had higher mortality rates compared to individuals without HIV, with mortality rates increasing as CD4 counts decreased. We replicated the historical CD4 count-based eligibility criteria for antiretroviral therapy (ART) initiation in Kenya.^16^ We modeled treatment only for individuals who achieved viral suppression and assumed that increasing proportion of people on ART become virally suppressed over time, reflecting the historical trends observed in Kenya (Table S12 and Figure S2). Additionally, we assumed that 72·9% of men and women with HIV will be virally suppressed by 2030, achieving the Joint United Nations Programme on HIV/AIDS (UNAIDS) 90-90-90 goals. Male circumcision coverage also followed historical trends in Kenya (Table S13 and Figure S3), and was assumed reach at least 90% in all ages by 2030.

We modeled oncogenic HPV infection in two groupings: infection with an HPV type targeted by the nonavalent HPV vaccine (HPV 16/18/31/33/45/52/58) and infection with other oncogenic HPV types (HPV 35/39/51/56/59/68) (Figure S1). Acquisition and progression of these two groups of infection were independent of each other and could occur simultaneously. While men could only be HPV-infected or susceptible, women in the model had additional HPV-associated states of cervical intraepithelial neoplasia (CIN) grades 1 to 3 and cervical cancer. Further, women who cleared HPV infection develop natural immunity against reinfection with the same HPV group that wanes over time. Women with HIV had a higher probability of HPV acquisition, persistence, and progression to CIN, and reduced regression from CIN states.^4^^,^^17^ Specifically, HPV acquisition and CIN progression increased with decreasing CD4 count while HPV clearance and CIN regression decreased with decreasing CD4 count.

**Figure 1:**
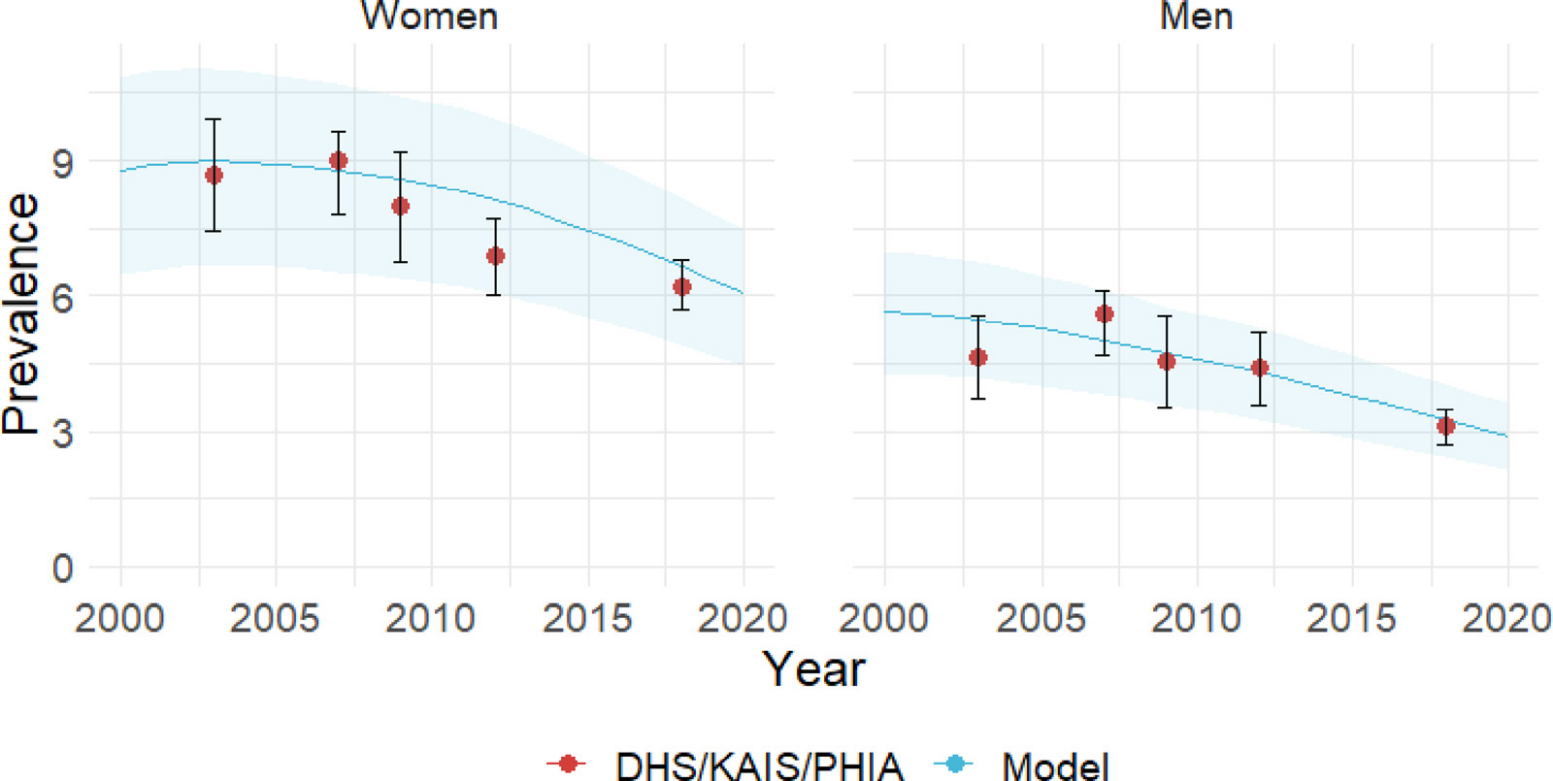
Model estimated HIV-prevalence among men and women between 2000-2020 (blue line) compared to data (red dots) from 2003 and 2008-2009 Demographic and Health Surveys (DHS), 2007 and 2012 Kenya AIDS Indicator Surveys (KAIS), and 2018 Population-based HIV Impact Assessment (PHIA). Shaded areas represent interquartile ranges of the model estimates.

**Figure 2:**
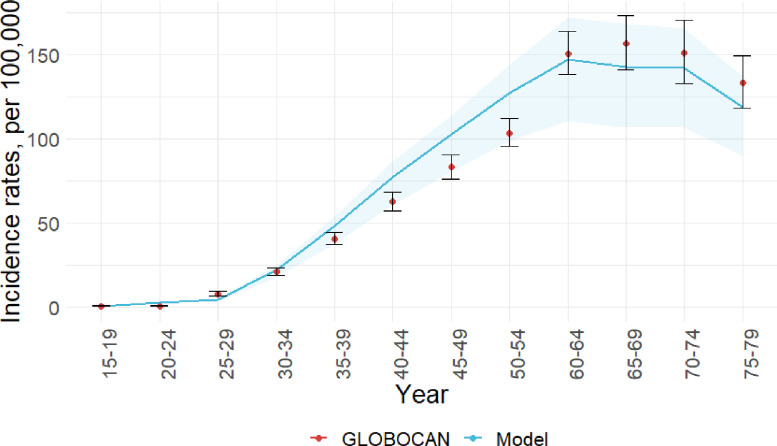
Model estimated age-specific cervical cancer incidence rates (blue line) compared to GLOBOCAN 2012 estimates (red dots). Shaded areas represent interquartile ranges of the model estimates.

We calibrated the model by fitting epidemiological and clinical parameters, including HIV and HPV transmission probabilities and CIN progression rates, to reproduce HIV, HPV, and cervical cancer epidemiology. Specifically, we calibrated the model to HIV prevalence from nationally representative surveys (Figures 1, S6-7) and HPV prevalence from observational studies (Figure S8). Additionally, we fit cervical cancer incidence to GLOBOCAN 2012 estimates (Figure 2). We validated the HIV prevalence and cervical cancer incidence against recent national survey data (Figure 1) and GLOBOCAN 2020 (Figures S9-10). To represent uncertainty around select influential parameters, specifically HIV transmission probability, HPV transmission probability, and the risk of HIV acquisition among women infected with HPV relative to HPV-uninfected women, we varied these parameters within ranges derived from published estimates and calibration. We ran 100 iterations, with the model randomly selecting a value from the specified range for these three parameters at each iteration. Model dynamics are governed by a system of ordinary differential equations that are solved in MATLAB using a 4th-order Runge-Kutta numerical method. Detailed description of model processes, calibration, and parameters is included in the Appendix. All data used in model parameterization, calibration, and validation were previously published, therefore, ethical approval was not required for this study.

### HPV vaccination scenarios

We modeled the impact of nonavalent HPV vaccination on cervical cancer incidence over 50 years, with vaccination beginning in 2021. Specific scenarios are as follows:

1 No vaccination. This is the baseline for evaluating the impact of vaccination strategies.
2 Single-age-cohort vaccination. 90% of girls vaccinated by age 10. This scenario reflects Kenya's vaccination strategy in 2019.
3 Multi-age-cohort (MAC) vaccination. Girls aged 10-14 are vaccinated at 90% coverage. This scenario represents the vaccination strategy proposed by the WHO to eliminate cervical cancer.^11^
4 MAC plus moderate-coverage catch-up vaccination. Vaccinating girls aged 10-14 at 90% coverage, with one year of catch-up vaccination for women aged 15-24 years at 50% coverage.
5 MAC plus high-coverage catch-up vaccination. Vaccinating girls aged 10-14 at 90% coverage, with one year of catch-up vaccination for women aged 15-24 years at 80% coverage. Scenarios 4 and 5 reflect the strategies adopted by several high-income countries.
6 HPV-FASTER. Vaccinating girls aged 10-14 at 90% coverage, with one year of vaccination for women aged 15-44 at 80% coverage. This scenario is similar to the strategy proposed by Bosch et al based on clinical trial data showing high vaccine efficacy among mid-adult women up to aged 45.^18^ Although Bosch et al proposed HPV-based screening for women older than 30 years in addition to vaccination, we are modeling only the vaccination component of the strategy.

We assumed the nonavalent vaccine provided lifelong protection and is 100% efficacious against the HPV types targeted by the vaccine regardless of the recipients’ HIV status. Only girls and women who were not currently infected with a vaccine-targeted HPV type received protection from vaccination, consistent with data showing low vaccine efficacy in recipients infected with a vaccine-targeted HPV type at the time of vaccination.^19^ For all scenarios, we modelled a constant one-time cervical cancer screening coverage of 7·4% in women without HIV and 12·3% in women with HIV,^20^ both at ages 35-39 years. Screening was with visual inspection with acetic acid, and those diagnosed with ≥CIN2 were treated with cryotherapy. ART coverage and male circumcision prevalence were consistent across all scenarios. However, to assess effect modification on HPV vaccination impact by ART coverage, we conducted a sensitivity analysis by varying ART coverage levels (Tables S18-S19).

### Outcomes

To evaluate vaccination impact on cervical cancer, we estimated annual cervical cancer incidence rates from 2021 to 2070, which were age-standardized using the WHO 2000-2025 Standard Population distribution. We calculated the impact of vaccination by dividing incidence rates from scenarios with vaccination by the rates from the baseline no vaccination scenario in the same year. We also estimated the cumulative number of cervical cancer cases averted 30 and 50 years after vaccine introduction relative to no vaccination. To assess the impact of HPV vaccination on HIV burden, we estimated yearly relative percent reductions in HIV prevalence compared to no vaccination. We also estimated the cumulative number of HIV cases averted after 30 and 50 years of vaccination. For all outcome estimates, we report the median and the interquartile range (IQR) of the 100 iterations.

### Role of the funding source

Study funders had no role in study design, data collection, data analysis, data interpretation, or writing of the report. GL had full access to all of the data and the final responsibility to submit for publication.

## Results

### Vaccine impact on cervical cancer

**Figure 3:**
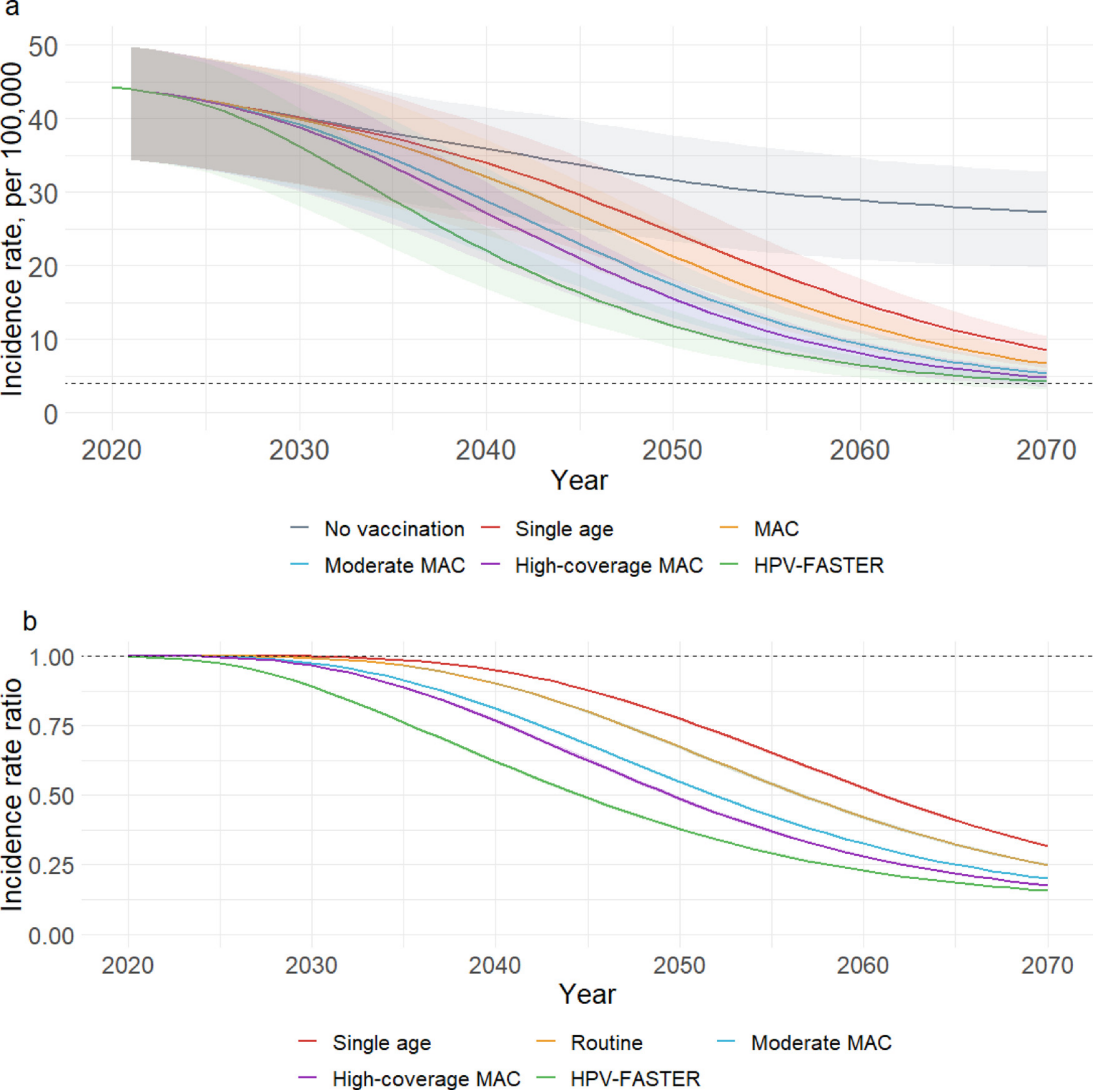
HPV vaccination impact on cervical cancer outcomes. a). Yearly age-standardized cervical cancer incidence rates from 2020-2070 by model scenario. The dashed line indicates the cervical cancer elimination threshold of 4 cases per 100,000 women. b). Yearly incidence rate ratios compared to no vaccination, with dashed horizontal line indicating no change relative to the scenario without vaccination. Shaded areas represent interquartile ranges of the model estimates. The HPV-FASTER scenario is similar to the strategy proposed by Bosch et al,^18^ except that only the vaccination component is modelled in this scenario.

The model-estimated cervical cancer incidence in 2021 was 44 per 100,000 women (IQR 35-50, Figure 3a). Without vaccination, the cervical cancer incidence rate was projected to be 32 per 100,000 women (IQR 23-38) in 2050 and 27 per 100,000 women (IQR 20-33) in 2070, resulting in 449,430 (IQR 345,822-509,880) and 745,975 (IQR 584,770-860,472) cumulative cases of cervical cancer since 2021.

By 2050, single-age-cohort vaccination was predicted to decrease cervical cancer incidence, relative to no vaccination, by 23%, averting 21,342 cases (IQR 18,235-25,311) of cervical cancer over 30 years (Table 1, figure 3b). With MAC vaccination, cervical cancer incidence was reduced by 33% and 35,038 cancer cases (IQR 29,501-41,407) were averted. Cervical cancer incidence was reduced by 45% with moderate-coverage catch-up and 51% with high-coverage catch-up, averting 57,370 (IQR 47,396-66,759) and 68,310 (IQR 55,612-79,092) cases of cervical cancer, respectively. The HPV-FASTER strategy reduced cervical cancer incidence by 62% and 98,783 cases (IQR 77,219-114,567) were averted.

**Table 1:** Projected cervical cancer incidence rates and HPV vaccine impact on cervical cancer in 2050 and 2070 relative to a baseline scenario with no vaccination.

	CC incidence, per 100,000	Percent reduction in incidence	Cumulative cancer cases averted
**2050**			
No vaccination	31.7 (23.3-37.7)	Reference	Reference
Single age cohort	24.5 (18.1-29.2)	22.5 (22.1-23.1)	21,342 (18,235-25,311)
MAC	21.3 (15.8-25.3)	32.7 (32.1-33.4)	35,038 (29,501-41,407)
Moderate catch-up	17.4 (12.9-20.6)	45.2 (44.7-46)	57,370 (47,396-66,759)
High-coverage catch-up	15.5 (11.5-18.3)	51.3 (50.5-52)	68,310 (55,612-79,092)
HPV-FASTER*	11.9 (9.0-13.8)	62.2 (61.7-62.7)	98,936 (77,697-114,505)
**2070**			
No vaccination	27.3 (19.7-32.8)	Reference	Reference
Single age cohort	8.5 (6.1-10.4)	68.3 (67.8-68.7)	164,529 (139,946-197,108)
MAC	6.7 (4.8-8.2)	75.1 (74.6-75.4)	206,115 (173,778-247,221)
Moderate catch-up	5.4 (3.9-6.5)	80 (79.6-80.3)	254,930 (212,510-303,618)
High-coverage catch-up	4.8 (3.5-5.8)	82.3 (82-82.6)	278,690 (229,763-330,529)
HPV-FASTER*	4.2 (3.2-5.0)	84.4 (83.9-84.6)	325,875 (263,001-382,380)

Abbreviation: HPV, human papillomavirus; CC, cervical cancer; MAC, multi-age cohort.

⁎ The HPV-FASTER scenario is similar to the strategy proposed by Bosch et al,^18^ except that only the vaccination component is modelled in this scenario.

By 2070, relative to no vaccination, cervical cancer incidence was reduced by 68% with single-age-cohort vaccination, 75% with MAC vaccination, 80% with moderate catch-up, 82% with high-coverage catch-up, and 84% with HPV-FASTER (Figure 3b). Over 50 years, single-age-cohort vaccination was expected to prevent 164,529 (IQR 139,946-197,108) cancer cases, while 206,115 (IQR 173,778-247,221) cases were averted with MAC vaccination, 254,930 (IQR 212,510-303,618) with moderate catch-up, 278,690 (IQR 229,763-330,529) cases with high-coverage catch-up, and 326,968 (IQR 262,991-383,806) cases with HPV-FASTER (Table 1).

### HPV Vaccination impact on HIV

**Figure 4:**
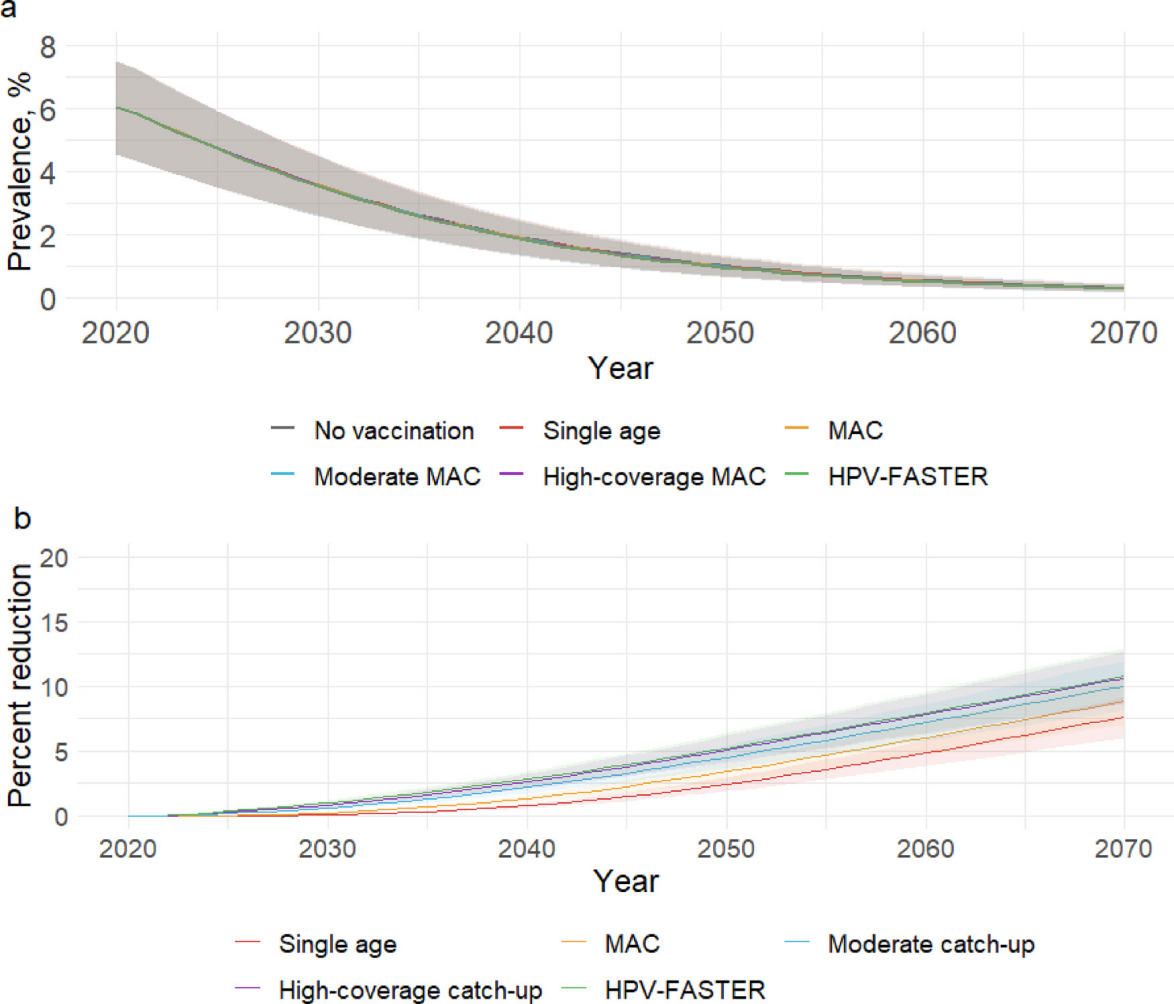
HPV vaccination impact on HIV burden in women. a) HIV prevalence among women over time by scenario. Because HIV prevalence was similar in all scenarios, the estimates and interquartile ranges for all scenarios overlap almost completely. b) Percent reduction in HIV prevalence relative to no vaccination. Shaded areas represent interquartile ranges of the model estimates. The HPV-FASTER scenario is similar to the strategy proposed by Bosch et al,^18^ except that only the vaccination component is modelled in this scenario.

HIV prevalence was projected to be 6·5% (IQR 4·4-7·5) among women and 2·9% (IQR 2·1-3·6) among men in 2021. Assuming the UNAIDS 90-90-90 goals are achieved by 2030, prevalence among women and men in the baseline scenario were predicted to decrease to 1·0% (IQR 0·7-1·3%) and 0·5% (IQR 0·3-0·7%) in 2050 and to 0·3% (IQR 0·2-0·4%) and 0·2% (IQR 0·1-0·2%) in 2070 (Table 2, figure 4a). In 2050, HIV prevalence in both women and men was reduced by a relative 5·4% or less in all scenarios relative to no vaccination (Table 2), equivalent to a <0·1% absolute difference. However, a cumulative 7,596 cases (IQR 5,018-12,627) of HIV were averted in women with single-age-cohort vaccination, 11,370 cases (IQR 7,548-18,702) with MAC vaccination, 17,183 cases (IQR 11,570-27,740) with moderate catch-up, 20,116 cases (IQR 13,533-32,301) with high-coverage catch-up, and 23,626 cases (IQR 15,811-37,286) with HPV-FASTER (Table 2). Because pre-vaccination HIV burden among men was lower compared to women, the number of HIV cases averted among men was proportionately lower even though the percent reductions in HIV prevalence were similar (Table 2).

**Table 2:** Projected HIV prevalence and HPV vaccination impact on HIV burden among women and men in 2050 and 2070 relative to a baseline scenario with no vaccination.

		Women			Men	
	HIV prevalence (%)	% reduction in prevalence	Cumulative HIV cases averted	HIV prevalence (%)	% reduction in prevalence	Cumulative HIV cases averted
**2050**						
No vaccination	1.0 (0.7-1.4)	Reference	Reference	0.5 (0.4-0.7)	Reference	Reference
Single age	1.0 (0.7-1.4)	2.4 (1.9-3.0)	7,596 (5,018-12,627)	0.5 (0.4-0.7)	2.2 (1.7-2.5)	3,491 (2,320-5,622)
MAC	1.0 (0.7-1.3)	3.4 (2.7-4.1)	11,370 (7,548-18,702)	0.5 (0.4-0.7)	3.1 (2.5-3.7)	5,352 (3,597-8,609)
Moderate catch-up	1.0 (0.7-1.3)	4.5 (3.6-5.4)	17,183 (11,570-27,740)	0.5 (0.4-0.7)	4.3 (3.5-5.1)	8,192 (5,630-13,000)
High-coverage catch-up	1.0 (0.7-1.3)	5.1 (4.1-6.2)	20,116 (13,533-32,301)	0.5 (0.4-0.7)	4.9 (4.0-5.8)	9,620 (6,636-15,268)
HPV-FASTER*	1.0 (0.7-1.3)	5.4 (4.4-6.6)	23,626 (15,811-37,286)	0.5 (0.4-0.7)	5.3 (4.3-6.2)	10,945 (7,651-17,366)
**2070**						
No vaccination	0.3 (0.2-0.5)	Reference	Reference	0.2 (0.1-0.2)	Reference	Reference
Single age	0.3 (0.2-0.4)	7.6 (6.1-9.1)	15,609 (9,916-27,192)	0.2 (0.1-0.2)	7.4 (5.9-8.9)	8,253 (5,156-13,666)
MAC	0.3 (0.2-0.4)	8.9 (7.1-10.6)	20,570 (13,284-35,553)	0.2 (0.1-0.2)	8.6 (6.9-10.3)	10,944 (6,959-18,009)
Moderate catch-up	0.3 (0.2-0.4)	10.0 (8.1-12.0)	27,568 (18,298-46,725)	0.2 (0.1-0.2)	9.8 (8.0-11.7)	14,604 (9,596-23,731)
High-coverage catch-up	0.3 (0.2-0.4)	10.6 (8.7-12.7)	31,145 (20,691-52,430)	0.2 (0.1-0.2)	10.4 (8.5-12.4)	16,444 (10,870-26,697)
HPV-FASTER*	0.3 (0.2-0.4)	11.0 (8.9-13.2)	34,981 (23,224-57,825)	0.2 (0.1-0.2)	10.8 (8.8-12.8)	17,970 (12,023-29,161)

Abbreviation: HPV, human papillomavirus; MAC, multi-age cohort

⁎ The HPV-FASTER scenario is similar to the strategy proposed by Bosch et al,^18^ except that only the vaccination component is modelled in this scenario.

Relative to no vaccination, HIV prevalence among women in 2070 was reduced by 7·6-11·0% in the vaccination scenarios, with similar relative reductions in men (Figure S13). However, the absolute changes in HIV prevalence due to vaccination were <0·1% (Table 2). The cumulative number of HIV cases averted in women after 50 years of vaccination was 15,609 (IQR 9,916-27,192) with single-age-cohort vaccination, 20,570 (IQR 13,284-35,553) with MAC vaccination, 27,568 (IQR 18,298-46,725) with moderate catch-up, 31,145 (IQR 20,691-52,430) with high-coverage catch-up, and 34,981 (IQR 23,224-57,825) with HPV-FASTER (Table 2). The number of HIV infections averted in men were approximately half of that in women (Table 2).

### Effect of ART on HPV vaccine impact

The impact of HPV vaccination on cervical cancer and HIV burden is slightly modified by ART coverage levels among people living with HIV (Table S19-S20). Findings from our sensitivity analyses show that HPV vaccination led to a greater reduction in cervical cancer incidence (Table S19) and HIV prevalence (Table S20) when ART coverage is lower.

## Discussion

HIV prevention and HPV vaccination have the potential to substantially decrease cervical cancer burden in Kenya in the next 50 years, particularly if young women up to age 24 are vaccinated. Compared to no vaccination, cervical cancer risk in 2070 was three times lower with single-age-cohort vaccination of 10-year-old girls and was up to 5·7 times lower with catch-up vaccination of women aged 15-24. Catch-up vaccination could prevent 90,401 to 114,161 cases of cervical cancer in addition to the 164,529 cases prevented through single-age-cohort vaccination. The total cumulative cervical cancer cases averted by 2070 was almost doubled with HPV-FASTER compared to single-age-cohort vaccination. In addition, HIV prevention can also reduce cervical cancer incidence on the population-level, as shown by the results from the no vaccination scenario and the sensitivity analyses.

Since catch-up vaccination and HPV-FASTER lead to higher vaccination coverage in older age groups, the impact of vaccination on cervical cancer incidence will be realized earlier. With catch-up vaccination and HPV-FASTER, 23-30% of the projected cumulative cases of cervical cancers by 2050 were prevented in our simulations, compared to 13% with single-age-cohort vaccination. These findings are consistent with post-vaccination HPV surveillance data from European and North American countries, which show that population-level HPV prevalence declined more rapidly and to a greater extent with catch-up vaccination (up to age 19 or 26) than with single-age-cohort vaccination.^20^ The HPV-FASTER strategy resulted in the greatest reduction of cervical cancer incidence in our analysis. Although the HPV-FASTER strategy had been found not to be cost-effective in settings with high coverage of cervical cancer screening,^21^ our results suggest that evaluation of HPV-FASTER in contexts with low screening coverage is warranted. Further investigation of HPV-FASTER in settings with significant HIV burden is also required. As women with HIV have high prevalence of HPV, countries with high HIV burden implementing HPV-FASTER may have to screen women with HIV for current HPV infection prior to vaccination to increase the cost-effectiveness of this strategy. While adding HPV-based screening before vaccination could increase the cost of delivering vaccines to adult women, the cost may be offset by cervical cancers prevented and/or treated as a result of the added screening.

While the WHO strongly recommends routine vaccination of girls 9-14 years old, its recommendation for vaccinating young women 15 and older is more tentative, leaving countries to decide whether to vaccinate young women.^22^ One consideration regarding vaccinating this population is the reduced vaccine effectiveness in young women due to more HPV infections compared to girls aged 9-14.^23^ To account for lower vaccine efficacy among HPV-infected individuals in our model, only girls and women who were susceptible to or have cleared previous infections with the types targeted by the nonavalent vaccine were conferred protection from HPV vaccination. The additional reduction in cervical cancer cases and incidence rates in the catch-up scenarios compared to the single-age-cohort and MAC vaccination scenarios demonstrate that older adolescents and young women can indeed benefit from HPV vaccination, and expanding eligibility to this age group can lead to population-level reduction in cancer burden. While we found a greater and earlier impact with catch-up vaccination compared to single-age-cohort and MAC vaccination, the difference between the two catch-up scenarios was moderate. Relative to moderate-coverage catch-up vaccination, high-coverage catch-up vaccination resulted in an additional 23,760 cases of cervical cancer averted over 50 years. This is likely because the proportion of women vaccinated in the moderate-coverage catch-up scenario reached the same level as in the high-coverage catch-up scenario within 10 years of vaccine introduction (Table S17 and Figure S12).

We modelled bidirectional interactions between HIV and HPV infections, such that HIV infection increased the risk of HPV acquisition and progression, and HPV infection increased HIV acquisition. Kenya has made marked progress in HIV prevention, leading to a decline in HIV prevalence in the last two decades.^5^ Assuming high coverage of ART and male circumcision was achieved by 2030, the model predicted that HIV prevalence among both men and women would decrease to less than 0·5%. Reflecting the decline in HIV burden, the model projected a 38% decline in cervical cancer incidence without HPV vaccination. Our findings are in line with those of Hall et al, which projected a 32% decline in cervical cancer incidence due to ART and male circumcision in Tanzania.^13^ Conversely, while women with HPV infection were assumed to have on average 2 times higher risk of acquiring HIV compared to women who are HPV-negative,^4^ HPV vaccination had minimal impact on the overall HIV burden. This is likely due to high coverage of ART and circumcision that led to low HIV incidence in the baseline scenario. Findings from our sensitivity analyses suggest that HPV vaccination may have a higher impact in settings with lower HIV prevention coverage. However, the cumulative number of HIV infections prevented as a result of vaccination was not trivial, with up to 27,812 cases averted in women and 14,693 cases averted in men over 50 years. Although men were not vaccinated, they indirectly benefited from the reduced HIV burden in women. These findings support a role for HPV vaccination in a comprehensive HIV prevention package that includes sexual and reproductive health services.^24^

Of note, we found that even in the highest coverage scenario (90% of 9-14-year-old girls and 80% of 15-44-year-old women vaccinated), cervical cancer elimination, defined as incidence of ≤4 per 100,000, was not achieved in Kenya by 2070. This is consistent with other modeling studies showing that cervical cancer elimination in LMICs may not be achievable even in the next century with vaccination alone.^12^^,^^25^ Additional investment in secondary prevention will be necessary to accelerate cervical cancer elimination in Kenya. Increasing screening coverage and treatment retention would prevent cancer among unvaccinated women and prevent cervical cancer caused by HPV types not targeted by the vaccine.^26^

A strength of our model is that HIV and HPV transmission occurred dynamically, allowing the model to capture herd effects from interventions. This enabled us to estimate the total, population-level effect of vaccination on cervical cancer in Kenya. In addition, we explicitly modelled the biological interactions between HIV and HPV infections, making it possible to isolate the effect of HPV vaccination on cervical cancer from the effect of HIV prevention on cervical cancer. Accurate projection of vaccination impact will be important as Kenya plans to meet the challenge of cervical cancer elimination amidst changing HIV epidemiology. Further, while the convergence of evidence that HPV infection increased the risk of HIV acquisition prompted questions regarding the effectiveness of HPV vaccination for HIV prevention, clinical trials to investigate these questions were not recommended due to ethical and financial concerns.^27^ Using a well-parameterized mathematical model, we were able to estimate the impact of HPV vaccination on HIV burden while circumventing the ethical and financial issues associated with observational studies or randomized controlled trials.

We acknowledge the study limitations and their impact on the findings. Sexual behavior patterns, including condom use and mixing, are highly influential on disease trends. For simplicity, we assumed these parameters remained constant. However, the accuracy of our projections of HIV and cervical cancer burden would be affected if changes in future sexual behavior patterns occur. Similarly, our predictions regarding the benefit of catch-up vaccination among young women aged 15-24 could be biased if sexual behaviors in this population change in the future. Due to a lack of national-level cervical cancer incidence data in Kenya, we calibrated our model to the cervical cancer incidence rates estimated by GLOBOCAN.^1^ These rates were derived from regional cancer registries in Kenya and from registries in neighboring countries, and may not be representative of the overall cancer incidence in Kenya. Therefore, our cancer incidence projection could be inaccurate if GLOBOCAN over- or underestimated the true cancer incidence. Further, vaccination coverage in our model scales up to the specified level immediately in all vaccination scenarios. In reality, such high coverage, especially among older adolescents and adult women, may be difficult to achieve rapidly, as had been demonstrated in Australia and the United States.^28^^,^^29^ If scale-up of vaccination is gradual, the incremental health benefits may be delayed. Additionally, HPV-FASTER may be even more challenging to implement. However, single-dose HPV vaccination, if efficacious, would simplify the logistics of expanded HPV vaccination. Finally, although HPV vaccination commenced in 2021 in our model, the full program roll-out in Kenya may be delayed due to the impact of the Coronavirus disease 2019 pandemic on healthcare budget and personnel. Assuming Kenya's healthcare system recovers to pre-pandemic conditions, our results will still be applicable if the vaccine program is delayed by one or two years.

In conclusion, cervical cancer burden in Kenya can be substantially reduced with HPV vaccination and HIV prevention. The reduction in cancer incidence is greater and occurs earlier when multiple age cohorts are included in the vaccination strategy. While the elimination threshold is not projected to be reached, substantial cervical cancer-associated morbidity and mortality can be averted in high HIV prevalence settings with more equitable HPV vaccine coverage. In addition, concurrent scale-up of cervical precancer screening and treatment will be needed to eliminate cervical cancer as a public health problem in Kenya.

### Author contribution

Conceptualization and design: GL, CJB, DRW, and RVB

Model adaption, calibration, and validation: GL, CJB, and DRW

Data review for parameterization and scenario definition: GL, NRM, CJB, DRW, and RVB

Scenario definition and analysis planning: all authors

Results interpretation and critical review: all authors

Review and approve final manuscript: all authors

### Data sharing

We did not collect participant data for this study, however, data used to parameterize, calibrate, and validate the model are available in the Supplemental Appendix.

## Declaration of interests

NRM is the recipient of an investigator-initiated award from Merck for a HPV vaccine study. RVB declares support from the National Institutes of Health and the Bill and Melinda Gates Foundation; and from Regeneron Pharmaceuticals for manuscript writing and abstract submission outside the submitted work. All other authors declare no competing interests.

## Appendix Supplementary materials

Image, application 1

Image, application 2
