# Supplementary material for: Impact of catch-up human papillomavirus vaccination on cervical cancer incidence in Kenya: A mathematical modeling evaluation of HPV vaccination strategies in the context of moderate HIV prevalence
Source: eClinicalMedicine. 2022 Feb 19;45:101306. doi: 10.1016/j.eclinm.2022.101306 (PMC8860915; doi:10.1016/j.eclinm.2022.101306)
Supplement: Supplementary file 1 [file mmc1.docx]

Supplementary Appendix:

Technical appendix for: Liu G, Mugo NR, Bayer C, et al. Impact of catch-up human papillomavirus vaccination on cervical cancer incidence in Kenya: A mathematical modeling evaluation of HPV vaccination strategies in the context of moderate HIV prevalence.
