# Supplementary material for: Impact of catch-up human papillomavirus vaccination on cervical cancer incidence in Kenya: A mathematical modeling evaluation of HPV vaccination strategies in the context of moderate HIV prevalence
Source: eClinicalMedicine. 2022 Feb 19;45:101306. doi: 10.1016/j.eclinm.2022.101306 (PMC8860915; doi:10.1016/j.eclinm.2022.101306)
Supplement: Supplementary file 2 [file mmc2.docx]

**Technical appendix for:**

Impact of catch-up human papillomavirus vaccination on cervical cancer incidence in Kenya: A mathematical modeling evaluation of HPV vaccination strategies in the context of moderate HIV prevalence

Gui Liu, PhD; Nelly R Mugo, MBChB MMeD; Cara Bayer, MS; Darcy White Rao, PhD; Maricianah Onono, PhD; Nyaradzo M Mgodi, MBChB; Zvavahera M Chirenje, MD; Betty W Njoroge, MBChB; Nicholas Tan, BS; Elizabeth A Bukusi, PhD; and Ruanne V Barnabas, MBChB

[I. Model overview 2](#_Toc93580022)

[II. Modules and parameter values 4](#_Toc93580023)

[a. Demography 4](#_Toc93580024)

[b. Sexual behavior 5](#_Toc93580025)

[c. Natural history 6](#_Toc93580026)

[i. HIV 6](#_Toc93580027)

[ii. HPV and cervical cancer 7](#_Toc93580028)

[e. Historical interventions 10](#_Toc93580029)

[i. HIV antiretroviral therapy (ART) 10](#_Toc93580030)

[ii. Condoms 11](#_Toc93580031)

[iii. Circumcision 11](#_Toc93580032)

[iv. HPV vaccination 12](#_Toc93580033)

[v. HPV screening and treatment 12](#_Toc93580034)

[III. Calibration and validation 13](#_Toc93580035)

[a. Calibration 13](#_Toc93580036)

[b. Validation 16](#_Toc93580037)

[c. Comparing model output to data 17](#_Toc93580038)

[IV. Additional results 20](#_Toc93580039)

[V. Sensitivity analyses 21](#_Toc93580040)

[VI. Differential equations 24](#_Toc93580041)

[a. Demography 26](#_Toc93580042)

[b. Sexual Behavior 27](#_Toc93580043)

[c. Transmission Probabilities 28](#_Toc93580044)

[d. Natural History and Interventions 29](#_Toc93580045)

[VII. References 33](#_Toc93580046)

# I. Model overview

For this modeling study, we used a deterministic, compartmental model parameterized to represent transmission and progression of HIV and oncogenic human papillomavirus (or high-risk HPV (hrHPV)) in Kenya, which has an HIV prevalence of 4.5% in 2019.^1^ The model was adapted from a previously published model fitted to KwaZulu-Natal, South Africa.^2^ The primary objective of the current modeling study is to evaluate and predict population-level impact of HPV vaccination on cervical cancer outcomes, while taking into account the changing HIV epidemiology and scaling-up of HIV prevention in Kenya.

Because the data we use to inform the model are not stratified by gender, our model population represents a primarily heterosexual population in Kenya. Men and women infected with hrHPV may clear the infection, and infections in women may progress through stages of precancerous lesions to cervical cancer (Figure S1). HIV progression is tracked by CD4+ T-cell (CD4) count and HIV RNA concentration (viral load), and infected individuals may achieve viral suppression with antiretroviral therapy (ART) beginning in 2005 (Figure S1). A key feature of our model is representation of the bidirectional interaction between HIV and HPV, whereby HIV infection increases the probability of HPV acquisition and the rate of disease progression and HPV infection increases the probability of HIV acquisition. We calibrate the model to fit to HIV, HPV, and cervical cancer epidemiology in Kenya.

Model dynamics are governed by a system of differential equations that are solved in MATLAB using a 4^th^-order Runge-Kutta numerical method. The model simulates events in discrete time with two-month intervals. At each time step, differential equations are evaluated to estimate population demographics and the number of persons in each infection, disease, or treatment compartment for the following time step. The dynamic nature of our transmission model captures population-level effects such as herd immunity.

This work was facilitated through the use of advanced computational, storage, and networking infrastructure provided by the Hyak supercomputer system at the University of Washington.

**Figure S1.** Model schematic illustrating HPV progression and HIV progression. We model HPV infection with high-risk HPV (hrHPV) types targeted by HPV vaccines (HPV 16, 18, 31, 33, 45, 52, and 58) and other high risk, non-vaccine targeted types (HPV 35, 39, 51, 56, 59 and 68). Arrows with * indicate processes that are affected by HIV infection, and arrows with ⱡ indicated processes affected by HPV infection.


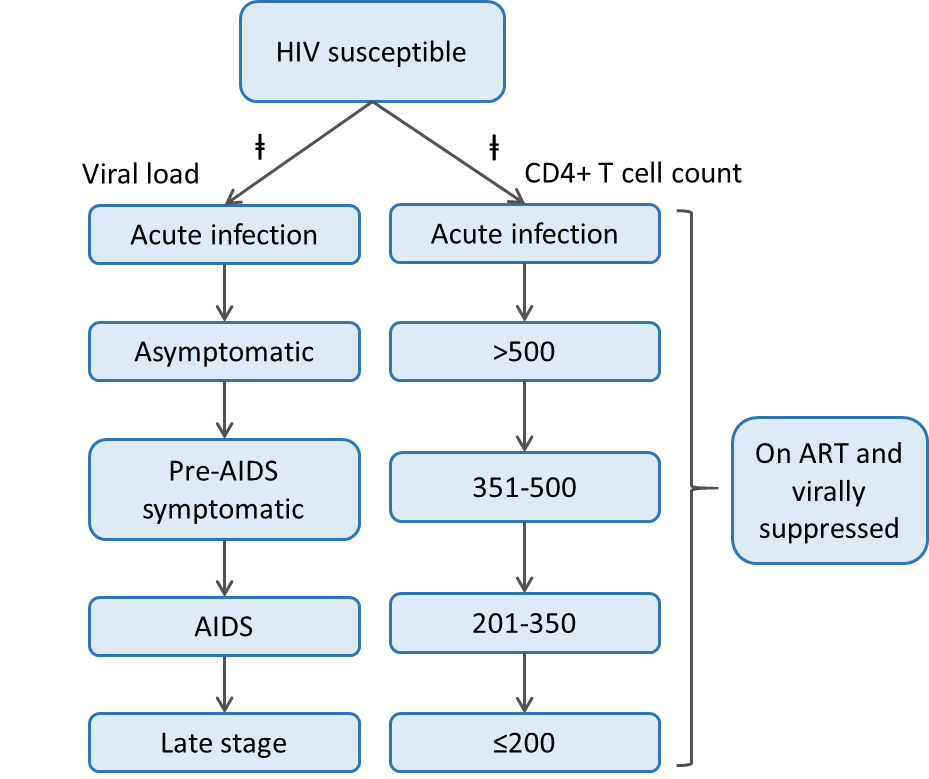

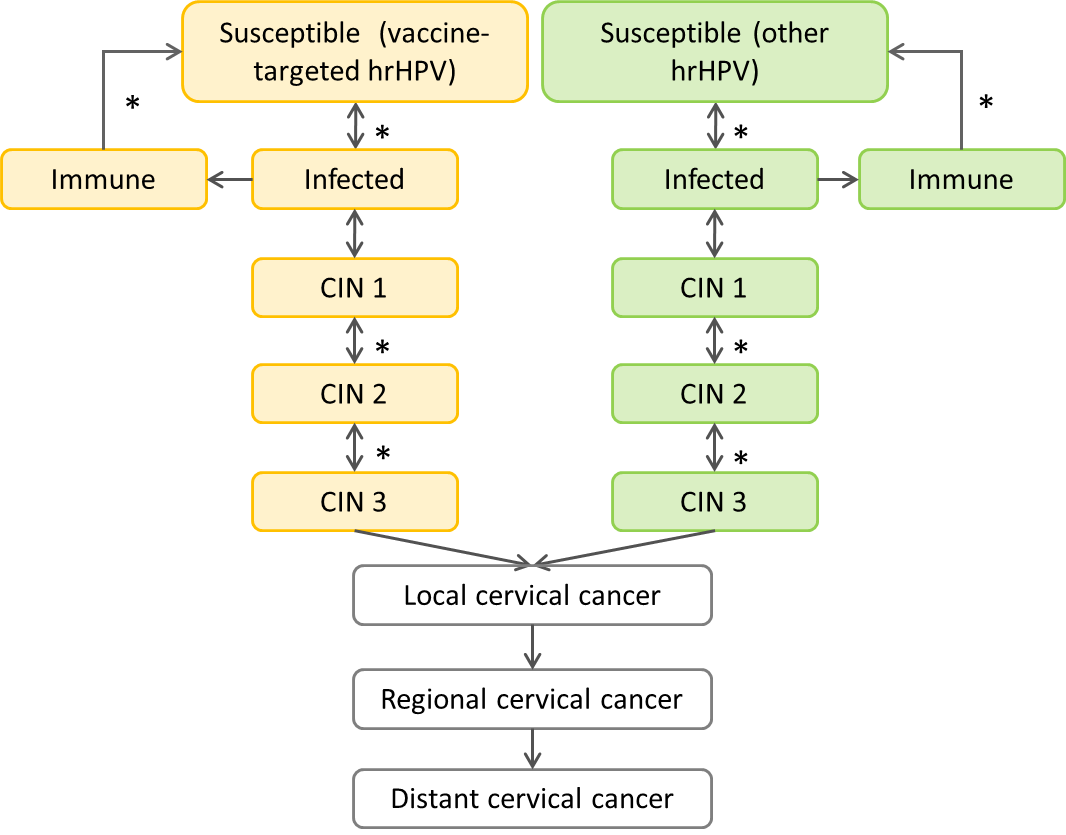


# II. Modules and parameter values

## a. Demography

We model a dynamic, open population of males and females aged 0 to 79 in Kenya in 5-year age groups. To allow HPV transmission dynamics and cervical cancer to equilibrate before introducing HIV in 1980, we initialize the model in 1925. The sex- and age-specific distribution of our initial population in 1925 is based data from United Nations Population Division (UNPD), which estimates Kenya population by sex and age starting in 1950.^3^ We fit exponential distributions for each sex and age group to extrapolate backwards to 1925 (Table S1).

At each two-month time step, the model calculates the number of births and deaths based on fertility and mortality rate inputs. The initial fertility rates are based on UNPD-estimated rates for 1960.^3^ We then scale down fertility rates to produce population distributions consistent with UNPD estimates from 1950-2020 as well as Kenya Census data from 2009 and 2019.^3-5^ We assume fertility rates for women living with HIV (WLHIV) with CD4 count of <500 cells/mm^3^ are 0.41-0.58 of women without HIV and the rates for WLHIV on ART or had CD4 >500 cells/mm3 are the same as women without HIV (Table S2).^6,7^ We further scale down fertility rates after 2020 to fit projected population estimates.

To age the population, one-fifth of each compartment moves to the next age group annually. Persons leave the population due to death or aging past 79. To model deaths, we apply age- and sex-specific mortality rates estimated by UNPD (Table S3).^3^ We assume linear changes in rates between 1950, 1985, 2010, and 2020, which are the years for which rates are available. After initializing HIV in 1980, we model background and HIV-specific mortality separately. To estimate background mortality rates not related to HIV, we subtract HIV-specific mortality^8,9^ from UNPD-estimated mortality rates.

**Table S1. Initial population size.** The population distribution by sex and 5-year age groups is projected backward from UNPD estimates for Kenya in 1950.

| Age Group | Initial Population Size | | Source |
| --- | --- | --- | --- |
|  | **Male** | **Female** | UNPD^3^ |
| 0 – 4 | 237966 | 240166 |  |
| 5 – 9 | 143534 | 145291 |  |
| 10 – 14 | 110242 | 110999 |  |
| 15 – 19 | 99096 | 97339 |  |
| 20 – 24 | 91121 | 86235 |  |
| 25 – 29 | 84685 | 75947 |  |
| 30 – 39 | 80170 | 68470 |  |
| 35 – 39 | 78914 | 63502 |  |
| 40 – 44 | 79282 | 61809 |  |
| 45 – 49 | 76761 | 63348 |  |
| 50 – 54 | 68455 | 62295 |  |
| 55 – 59 | 52798 | 53896 |  |
| 60 – 64 | 39427 | 45400 |  |
| 65 – 69 | 27875 | 36647 |  |
| 70 – 74 | 16553 | 25006 |  |
| 75 – 79 | 7905 | 14683 |  |
| TOTAL | 1294783 | 1251034 |  |

**Table S2. Baseline fertility rates per 1000 women by age.** These fertility rates are applied from 1925-1970, then scaled down thereafter. Age groups not shown in this table have fertility rates of 0.

| Age Group | HIV Uninfected | Source |
| --- | --- | --- |
| 15 – 19 | 0.182 | UNPD^3^ |
| 20 – 24 | 0.379 |  |
| 25 – 29 | 0.365 |  |
| 30 – 34 | 0.306 |  |
| 35 – 39 | 0.219 |  |
| 40 – 44 | 0.119 |  |
| 45 – 49 | 0.042 |  |

**Table S3. UNPD-estimated age- and sex-specific background mortality rates for 1950, 1985, 2000, and 2020.^3^** We assume linear changes in background mortality between the years. These rates do not include deaths caused by HIV, which are estimated separately.

|  | 1925-1950 | | By 1985 | | By 2000 | | By 2020 | | Source |
| --- | --- | --- | --- | --- | --- | --- | --- | --- | --- |
| **Age Group** | **Male** | **Female** | **Male** | **Female** | **Male** | **Female** | **Male** | **Female** | UNPD^3^ |
| **0 – 4** | 0.103 | 0.088 | 0.047 | 0.040 | 0.071 | 0.062 | 0.036 | 0.027 |  |
| **5 – 9** | 0.009 | 0.009 | 0.003 | 0.002 | 0.004 | 0.003 | 0.001 | 0.001 |  |
| **10 – 14** | 0.005 | 0.005 | 0.002 | 0.002 | 0.003 | 0.002 | 0.001 | 0.001 |  |
| **15 – 19** | 0.006 | 0.006 | 0.003 | 0.002 | 0.004 | 0.003 | 0.001 | 0.001 |  |
| **20 – 24** | 0.009 | 0.007 | 0.004 | 0.003 | 0.006 | 0.005 | 0.002 | 0.001 |  |
| **25 – 29** | 0.010 | 0.008 | 0.005 | 0.004 | 0.009 | 0.009 | 0.003 | 0.002 |  |
| **30 – 34** | 0.011 | 0.009 | 0.006 | 0.005 | 0.012 | 0.012 | 0.004 | 0.003 |  |
| **35 – 39** | 0.012 | 0.010 | 0.007 | 0.006 | 0.016 | 0.016 | 0.005 | 0.004 |  |
| **40 – 44** | 0.014 | 0.012 | 0.009 | 0.007 | 0.019 | 0.017 | 0.007 | 0.005 |  |
| **45 – 49** | 0.016 | 0.013 | 0.011 | 0.008 | 0.023 | 0.018 | 0.009 | 0.006 |  |
| **50 – 54** | 0.020 | 0.016 | 0.015 | 0.010 | 0.027 | 0.019 | 0.012 | 0.008 |  |
| **55 – 59** | 0.025 | 0.020 | 0.019 | 0.013 | 0.033 | 0.022 | 0.016 | 0.010 |  |
| **60 - 64** | 0.035 | 0.030 | 0.028 | 0.020 | 0.044 | 0.028 | 0.024 | 0.015 |  |
| **65- 69** | 0.050 | 0.045 | 0.042 | 0.032 | 0.061 | 0.040 | 0.036 | 0.024 |  |
| **70 - 74** | 0.075 | 0.071 | 0.066 | 0.054 | 0.091 | 0.061 | 0.056 | 0.039 |  |
| **75 - 79** | 0.116 | 0.110 | 0.108 | 0.091 | 0.138 | 0.096 | 0.089 | 0.066 |  |

## b. Sexual behavior

In our model, sexual activity begins in the 10-14 age group. In each sexually active age group, we divide the population into low-, medium-, or high-risk groups with variable rates of partnership formation. The distribution of these risk groups by sex and age is informed by self-reported sexual behaviors data from 15-54 year old respondents in the 2014 Kenya Demographic and Health Survey (DHS),^10^ and is calculated as the percentage of the cohort who reported 0-1 partner (low risk), 2-4 partners (moderate risk), or ≥5 partners (high risk) in the past year. We then calibrate risk group distribution for ages 15-54 to fit to observed HIV and HPV data. Individuals aged 10-14 are assumed to be predominately low risk, while the risk distribution for ages 55 and older is extrapolated from the 50-54 age group (Table S4).

Similarly, while the initial sexual partnership inputs are informed by the 2014 Kenya DHS data,^10^ we calibrate the yearly partner change rates in each risk group by sex and age to fit to observed disease data (Table S5). This approach partially compensates for reporting biases and the lack of partnership concurrency in our model. Our compartmental model structure is not equipped to represent concurrent partnerships. Therefore, using the number of sex partners as reported would underestimate the rate of HPV and HIV transmission.

Using methods similar to other models, patterns of sexual contact in our model are characterized by age and sexual risk groups.^11^ The degree of mixing is defined by the parameter, $\epsilon$ ($\epsilon_{\alpha}$ for mixing by age and $\epsilon_{r}$ for mixing by risk group), which ranges from 0, indicating completely assortative (like-with-like), to 1, indicating random mixing that is proportional to compartment size. Based on studies that show that Kenyan women are on average 4-7 years younger than their male partners,^12^ we assume an age mixing matrix with $\epsilon_{\alpha}=0.2$. For mixing by risk group, we assume $\epsilon_{r}=0.3$.

Because we model heterosexual contact, the modeled number of partnerships must be the same for men and women. However, the observed data we used to inform these parameters are subject to selection and response biases, resulting in imbalances. To correct this, we adjust contact rates such that the number of partners for men equals the number of partners for women.

**Table S4. Proportion of individuals in low-, medium-, and high-risk groups by sex and age.** The risk group distribution for those older than 55 is extrapolated from the 50-54 age group.

|  | Males | | | Females | | | Source |
| --- | --- | --- | --- | --- | --- | --- | --- |
| **Age Group** | **Low-Risk** | **Medium-Risk** | **High-Risk** | **Low-Risk** | **Medium-Risk** | **High-Risk** | 2014 Kenya DHS^10^ / calibrated |
| **10 – 14** | 0.972 | 0.026 | 0.002 | 0.972 | 0.026 | 0.002 |  |
| **15 – 19** | 0.779 | 0.216 | 0.004 | 0.757 | 0.234 | 0.009 |  |
| **20 – 24** | 0.564 | 0.424 | 0.012 | 0.595 | 0.377 | 0.028 |  |
| **25 – 29** | 0.611 | 0.365 | 0.023 | 0.635 | 0.354 | 0.010 |  |
| **30 – 34** | 0.656 | 0.331 | 0.013 | 0.689 | 0.301 | 0.010 |  |
| **35 – 39** | 0.717 | 0.275 | 0.008 | 0.730 | 0.263 | 0.007 |  |
| **40 – 44** | 0.732 | 0.262 | 0.006 | 0.781 | 0.213 | 0.005 |  |
| **45 – 49** | 0.807 | 0.188 | 0.005 | 0.826 | 0.169 | 0.005 |  |
| **50 – 54** | 0.891 | 0.106 | 0.004 | 0.891 | 0.106 | 0.004 |  |
| **55 – 79** | 0.891 | 0.106 | 0.004 | 0.891 | 0.106 | 0.004 |  |

**Table S5. Average number of partners in each risk group by sex and age.** Estimates for those older than 55 is extrapolated from the 55-60 age group.

|  | Males | | | Females | | | Source |
| --- | --- | --- | --- | --- | --- | --- | --- |
| **Age Group** | **Low-Risk** | **Medium-Risk** | **High-Risk** | **Low-Risk** | **Medium-Risk** | **High-Risk** | 2014 Kenya DHS^10^ / calibrated |
| **10 – 14** | 0.03 | 0.00 | 0.00 | 0.03 | 0.01 | 0.00 |  |
| **15 – 19** | 0.23 | 2.45 | 5.41 | 0.50 | 2.45 | 6.41 |  |
| **20 – 24** | 0.87 | 2.58 | 9.78 | 0.87 | 2.58 | 11.08 |  |
| **25 – 29** | 0.87 | 2.33 | 11.10 | 0.90 | 2.33 | 10.15 |  |
| **30 – 34** | 0.93 | 2.11 | 10.15 | 0.95 | 2.11 | 9.76 |  |
| **35 – 39** | 0.93 | 2.08 | 9.67 | 0.93 | 2.08 | 9.67 |  |
| **40 – 44** | 0.93 | 2.06 | 8.28 | 0.93 | 2.06 | 8.28 |  |
| **45 – 49** | 0.93 | 2.00 | 7.35 | 0.93 | 2.00 | 7.35 |  |
| **50 – 54** | 0.92 | 1.90 | 7.03 | 0.92 | 1.90 | 7.03 |  |
| **55 – 79** | 0.92 | 1.85 | 2.50 | 0.92 | 1.85 | 2.50 |  |

## c. Natural history

### i. HIV

HIV begins in 1980 in our model with an initial prevalence of 0.5% in ages 15-54 and 0.2% in other age groups. HIV infection occurs either through mother-to-child transmission or heterosexual contact. We model mother-to-child transmission rates that decrease over time to reflect improvements in services for pregnant women living with HIV (Table S6).^13,14^ The force of HIV infection is estimated as a function of sexual mixing (by age and sexual risk group), HIV prevalence in the opposite sex, male circumcision, HIV viral load, and, among women, their current HPV infection status. The risk of HIV transmission is highest during the acute stage of infection. Risk decreases during the asymptomatic phase of HIV infection before increasing in the pre-AIDS symptomatic and AIDS stages.^15-18^ We base HIV transmission per act in the asymptomatic stage on literature^19^ and apply risk multipliers across the other stages of infection (Tables S6).^15-18,20^ We assume male-to-female transmission probability is equal to female-to male transmission probability across all viral load stages. As a proxy for decreased sexual activity due to advanced disease during late-stage HIV, we reduce HIV per-act transmission to be 10% of the AIDS rate.^18^ Based on the evidence that HPV, like other sexually transmitted infections, increased the risk of HIV acquisition, we assume that women with an HPV infection have between 1.4-2.2 times higher risk of acquiring HIV.^21,22^ Eleven observational studies have been done to evaluate the association between HPV and HIV acquisition in women.^23-33^ In five of the observational studies, HPV infection was ascertained one or more years before HIV seroconversion.^23,26,27,31,32^ Because most HPV infections are transient and clear without intervention in less than one year,^34^ measurement bias related to the exposure is likely in these studies. One study did not adjust for any confounders despite a high prevalence of herpes simplex virus 2 among its study participants.^30^ Of the remaining five studies, four found that HPV infections significantly increased HIV risk^24,25,28,33^ while one found no association.^29^ All but two of the observational studies^32,33^ were included in at least one of two meta-analyses summarizing the association between HPV infection and HIV acquisition.^21,22^

The transition rates between CD4 count stages and viral load stages are based on literature describing the average duration in each CD4 and viral load stage by sex and age (Tables S7).^16,35-37^ Starting in 2005, we model individuals with ART-mediated viral suppression. HIV-associated mortality rates with untreated HIV are estimated from studies of untreated persons living with HIV and depend on CD4 cell count and age (Table S8).^9,38,39^ As a result of these combination of disease progression and mortality rates, untreated women have a longer average life expectancy than untreated men. In addition, children under five have the highest HIV-specific mortality, and adults >50 years have HIV mortality rates two times that of persons aged 5-49.^8,40^ HIV-associated mortality for people on ART treatment is relative to background mortality, and decreases over time to reflect improvement in baseline health among persons initiating treatment.^41-43^ From 2004 to 2011, HIV-associated mortality among people with HIV on ART is 1.5 times the background rate. This rate decreases to 1.4, 1.25, and 1.15 times the background mortality rate in 2011, 2015, and 2016, respectively.

**Table S6.** Annual HIV transmission rates by route of transmission. To capture uncertainty related to the values of select influential parameters, we let the probability of HIV transmission via sexual contact vary within a range.

| Route | Value | Risk multipliers by viral load stages | | | | | Reference |
| --- | --- | --- | --- | --- | --- | --- | --- |
|  |  | Acute | Asymptomatic | Pre-AIDS symptomatic | AIDS | Late-stage |  |
| Mother to child |  |  |  |  |  |  |  |
| Before 2004  By 2007  By 2013 | 0.42  0.32  0.20 | 1.0 | 1.0 | 1.0 | 1.0 | 1.0 | ^13,14^ |
| Sexual contact | 0.0008-0.0012 | 9.0 | 1.0 | 2.5 | 7.0 | 0.7 | ^15-20^ |

**Table S7. Average duration of time (in years) spent in each CD4 stage and viral load stage with untreated HIV by sex and age.** However, the actual time spent in each state is lower due to the effects of background and disease-specific mortality rates. We estimate time in the asymptomatic stage such that the total time spent in all viral load stages matches the total time spent in CD4 stages.

| HIV states | Males | | | Females | | | Reference |
| --- | --- | --- | --- | --- | --- | --- | --- |
|  | 0-4 | 5-49 | 50-79 | 0-4 | 5-49 | 50-79 |  |
| CD4 counts |  |  |  |  |  |  |  |
| Acute | 0.25 | 0.25 | 0.25 | 0.25 | 0.25 | 0.25 | ^35,37^ |
| ≥501 | 0.25 | 0.25 | 0.25 | 0.93 | 0.93 | 0.29 |  |
| 351-500 | 3.56 | 3.56 | 2.85 | 3.71 | 3.71 | 3.34 |  |
| 201-350 | 4.67 | 4.67 | 4.51 | 4.68 | 4.68 | 4.23 |  |
| ≤200 | 2.13 | 3.70 | 1.85 | 2.13 | 3.70 | 1.85 |  |
| Viral load |  |  |  |  |  |  |  |
| Acute | 0.25 | 0.25 | 0.25 | 0.25 | 0.25 | 0.25 | ^16,36^ |
| Asymptomatic | 5.87 | 6.60 | 3.88 | 5.87 | 7.44 | 4.13 |  |
| Pre-AIDS symptomatic | 4.00 | 4.00 | 4.00 | 4.00 | 4.00 | 4.00 |  |
| AIDS | 0.75 | 0.75 | 0.75 | 0.75 | 0.75 | 0.75 |  |
| Late-stage | 0.83 | 0.83 | 0.83 | 0.83 | 0.83 | 0.83 |  |

**Table S8. HIV-specific mortality by CD4 cell count and age.**

| Age Group | Acute | CD4 ≥500 | CD4 350-500 | CD4 200-350 | CD4 ≤200 | Reference |
| --- | --- | --- | --- | --- | --- | --- |
| 0 – 4 | 0 | 0.4700 | 0.4700 | 0.4700 | 0.4700 | ^8,9,38-40^ |
| 5 – 49 | 0 | 0.0035 | 0.0255 | 0.0455 | 0.2655 |  |
| 50 – 79 | 0 | 0.0071 | 0.0511 | 0.0911 | 0.5311 |  |

######

### ii. HPV and cervical cancer

The model simulation begins in 1925 with an initial HPV prevalence of 20% among 15-44-year-old men and women. Because we are primarily interested in cervical cancer outcomes, our model represents infection with the HPV types classified as high risk, or oncogenic, by the International Agency for Research on Cancer.^44^ Infections are due to either vaccine-targeted hrHPV types (HPV 16, 18, 31, 33, 45, 52, and 58) or non-vaccine-targeted hrHPV types (HPV 35, 39, 51, 56, 59 and 68). Per-coital HPV transmission probability is based on literature^45^ then calibrated (Table S9). We assume that transmission probability of non-vaccine-targeted HPV type is higher than vaccine-targeted HPV type to reproduce type distribution of HPV infections in sub-Saharan Africa.^46,47^ HPV transmission among women with regional or distant cervical cancer is reduced by 50%. We set that male-to-female and female-to-male transmission probabilities to be equal. However, we assume that men do not develop natural immunity.^48^ Women develop partial immunity against reinfection with the same HPV type group (vaccine-type or non-vaccine type) that wanes at an annual rate of 0.024 (Table S9).**^49^** In our model, women with persistent HPV infection can progress to precancerous lesions (represented as cervical intraepithelial neoplasia (CIN) grades 1, 2, or 3) and cervical cancer (categorized as local, regional, or distant) as shown in Figure S1. CIN 1, 2, and 3 can regress and HPV infection can clear naturally. CIN progression and regression rates were based on data previously described by Tan et al. (Supplement Table S23).^2^ The transition rates were defined separately for nonavalent vaccine-targeted hrHPV types and other hrHPV types. The original model used by Tan et al included additional transitional states. We reweighted transitions by types and consolidated transitions to better match our current HPV type grouping and natural history structure. For reweighting purposes, we assumed the type distribution of CIN2 to be the average of the type distributions for low-grade and high-grade lesions. After the reweighting, transition rates for each infection type (vaccine-targeted or non-vaccine-targeted) were manually calibrated (Table S9).

Individuals with HIV have higher rates of HPV acquisition, immunity waning, and disease progression, and lower rates of HPV clearance and CIN regression.^50^ The effect size of HIV on HPV natural history is inversely correlated with CD4 cell count and is represented by risk multipliers on HPV infection acquisition and clearance and CIN progression or regression in women without HIV. The risk multipliers are initially based on literature then calibrated to fit to observed data (Table S9). Women on ART have lower risk of HPV infection and cervical precancer lesions compared to untreated women living with HIV and similar HPV prevalence compared to women without HIV.^51,52^ However, sub-Saharan Africa women living with HIV continue to have elevated cancer incidence rates despite being on ART.^53,54^ Based on these data, we assume women on treatment and virally suppressed have HPV acquisition and disease progression rates comparable to women without HIV, but HPV clearance, disease regression rates, and cervical cancer-associated mortality are equivalent to untreated women with high (>500) CD4 count. WLHIV also have higher cervical cancer-associated mortality rates than women without HIV,^55^ with the mortality rates increasing with decreasing CD4 count (Table S10).^56^ The effect of HIV on mortality decreases as cervical cancer progresses.^56^

Because the model does not track infection duration, we use age multipliers as proxies for HPV persistence. The multipliers are applied to both HPV types and scaled up linearly across each age grouping (Table S11). The age multipliers are based on those previously described by Tan et al., 2018 in Supplement Table S25.^2^ The multiplier values are the average of the vaccine-type and non-vaccine-type relative risks after rate adjustment.

**Table S9. HPV-related transition rates per year.** Multipliers are relative risks of transitioning between HPV states for women with HIV compared to women without HIV, and depended on CD4 count and ART status. To capture uncertainty related to the values of select influential parameters, we let the probability of vaccine-targeted HPV transmission vary within a range.

| Parameter description | Without HIV | | Multipliers with HIV | | | | | |
| --- | --- | --- | --- | --- | --- | --- | --- | --- |
|  | **Value** | **Reference** | **On ART** | **CD4 ≥500** | **CD4 350-500** | **CD4 200-350** | **CD4 ≤200** | **Reference** |
| Per-partner transmission probability |  |  |  |  |  |  |  |  |
| Vaccine-targeted | 0.008-0.014 | ^45^, calibrated | 1 | 2.14 | 2.39 | 2.54 | 2.78 | ^22,57^, calibrated |
| Non-vaccine-targeted | 0.016 | Calibrated |  |  |  |  |  |  |
| HPV to CIN 1 |  |  |  |  |  |  |  |  |
| Vaccine-targeted | 0.586 | Calibrated | NA | NA | NA | NA | NA |  |
| Non-vaccine-targeted | 0.266 | Calibrated |  |  |  |  |  |  |
| CIN 1 to CIN 2 |  |  |  |  |  |  |  |  |
| Vaccine-targeted | 0.182 | Calibrated | 1 | 1.05 | 1.47 | 1.89 | 2.31 | Calibrated |
| Non-vaccine-targeted | 0.065 | Calibrated |  |  |  |  |  |  |
| CIN 2 to CIN 3 |  |  |  |  |  |  |  |  |
| Vaccine-targeted | 0.169 | Calibrated | 1 | 1 | 1.1 | 1.2 | 1.4 | Calibrated |
| Non-vaccine-targeted | 0.076 | Calibrated |  |  |  |  |  |  |
| CIN3 to local cancer |  |  |  |  |  |  |  |  |
| Vaccine-targeted | 0.002 | Calibrated | NA | NA | NA | NA | NA |  |
| Non-vaccine-targeted | 0.001 | Calibrated |  |  |  |  |  |  |
| Local to regional cancer | 0.020 | ^58^ |  |  |  |  |  |  |
| Regional to distant cancer | 0.025 | ^58^ |  |  |  |  |  |  |
| HPV to immune in women |  |  |  |  |  |  |  |  |
| Vaccine-targeted | 1.772 | Calibrated | 0.6 | 0.6 | 0.55 | 0.45 | 0.30 | Calibrated |
| Non-vaccine-targeted | 1.768 | Calibrated |  |  |  |  |  |  |
| HPV to susceptible in men |  |  |  |  |  |  |  |  |
| Vaccine-targeted | 1.238 | Calibrated | 0.6 | 0.6 | 0.55 | 0.45 | 0.30 | Calibrated |
| Non-vaccine-targeted | 1.241 | Calibrated |  |  |  |  |  |  |
| CIN 1 to HPV |  |  |  |  |  |  |  |  |
| Vaccine-targeted | 0.360 | Calibrated | 0.6 | 0.6 | 0.55 | 0.45 | 0.30 | Calibrated |
| Non-vaccine-targeted | 0.423 | Calibrated |  |  |  |  |  |  |
| CIN2 to CIN 1 |  |  |  |  |  |  |  |  |
| Vaccine-targeted | 0.494 | Calibrated | 0.93 | 0.93 | 0.853 | 0.698 | 0.465 | Calibrated |
| Non-vaccine-targeted | 0.301 | Calibrated |  |  |  |  |  |  |
| CIN 3 to CIN 2 |  |  |  |  |  |  |  |  |
| Vaccine-targeted | 0.102 | Calibrated | 1.02 | 1.02 | 0.935 | 0.765 | 0.51 | Calibrated |
| Non-vaccine-targeted | 0.101 | Calibrated |  |  |  |  |  |  |
| Natural immunity waning | 0.024 | ^49^ | 1.42 | 1.42 | 1.57 | 1.97 | 2.83 | Calibrated |
| Natural immunity protection | 70% | ^59^ | NA | NA | NA | NA | NA |  |

**Table S10.** Cervical cancer-associated mortality by cancer stage and CD4 cell count among women living with HIV.

|  | Local | Regional | Distant | Reference |
| --- | --- | --- | --- | --- |
| **HIV uninfected** | 0.0624 | 0.1502 | 0.5207 | ^55,56^ |
| **HIV-positive on ART** | 0.2071 | 0.2418 | 0.5207 |  |
| **CD4 ≥500** | 0.2071 | 0.2418 | 0.5207 |  |
| **CD4 350-500** | 0.2515 | 0.2936 | 0.5207 |  |
| **CD4 200-350** | 0.3052 | 0.3563 | 0.5207 |  |
| **CD4 ≤200** | 0.4489 | 0.5240 | 0.5207 |  |

**Table S11.** Age multipliers on transition rates.

| Transition rate | 25-49 | 50-69 | 70-79 | Reference |
| --- | --- | --- | --- | --- |
| HPV infection to CIN1 progression | 0.49 | 0.35 | 0.35 | Calibrated |
| CIN1 to CIN2 progression | 1.17 | 1.42 | 1.42 |  |
| CIN2 to CIN3 progression | 1.79 | 2.54 | 3.04 |  |
| CIN3 to cervical cancer progression | 10.86 | 25.72 | 30.64 |  |
| HPV infection clearance to immune (females) or susceptible (males) | 0.79 | 0.51 | 0.45 |  |
| CIN1 to HPV infection regression | 1.00 | 1.00 | 1.00 |  |
| CIN2 to CIN1 regression | 1.00 | 1.00 | 1.00 |  |
| CIN3 to CIN2 regression | 0.77 | 0.27 | 0.14 |  |

## e. Historical interventions

### i. HIV antiretroviral therapy (ART)

Beginning in 2005, we model the effects of ART in populations that achieve viral suppression. People living with HIV who initiate ART without achieving viral suppression are assumed to have no benefit from treatment and are not tracked in our model. Populations in the ART/viral suppression compartment have reduced HIV-associated mortality, which also decreases over time as the average CD4 count at ART initiation increases.^41-43,60^ Additionally, we assume no onward transmission of HIV with ART.^61,62^ And women on ART have the same fertility rates as women without HIV.^7^

We define ART coverage as the percentage of all persons with HIV who are on treatment and virally suppressed. Reflecting historical treatment eligibility policy in Kenya,^63^ only people with CD4 count ≤200 cells/µL are put on ART in 2005. The ART initiation threshold is subsequently raised to CD4 <250 cells/mm^3^ in 2007, CD4 ≤350 cells/mm^3^ in 2011, and to CD4 ≤500 cells/mm^3^ in 2014. Finally, anyone with HIV regardless of CD4 cell count is eligible for ART from 2016 on, including those with acute infection. Our model recreates the historical ART coverages reported by the Kenya Ministry of Health.^64-66^ However, due to treatment non-adherence and discontinuation as well as the CD4-based eligibility criteria,^67,68^ the proportion of viral suppression among all people living with HIV was likely lower than the reported historical ART coverage levels, especially in the earlier years of ART. To adjust for this, we assume that 43% of people on ART are virally suppressed before 2013.^64^ We increase the proportion virally suppressed among people on ART linearly from 43% to 75% between 2013-2015,^64^ and from 75% to 90% from 2015 to 2018.^66^ The proportions of all people with HIV who are virally suppressed in our model is listed in Table S12 and represented in Figure S2. Given the current trajectory, we assume that Kenya will achieve the Joint United Nations Programme (UNAIDS) on HIV/AIDS 90-90-90 goal to have 72.9% of all people living with HIV virally suppressed by 2030. We do not model ART discontinuation (i.e., loss of viral suppression). As a result, the cumulative probability of being on ART increases with age.

Trends in HIV-associated mortality on treatment mirror changes to the ART initiation threshold to reflect higher baseline health among persons initiating treatment over time. HIV-associated mortality with treatment is relative to background mortality.^41-43^ From 2004 to 2011, HIV-associated mortality among people with HIV on ART is 0.5 times the background rate. This rate decreases to 0.4, 0.25, and 0.15 times the background mortality rate in 2011, 2015, and 2016, respectively.

**Table S12. Proportion of persons living with HIV on ART and virally suppressed Kenya.** Guided by viral suppression proportion among respondents of KAIS 2012 and the 2016 Kenya AIDS Progress Report and assuming an increasing proportion of individuals on ART achieve viral suppression over time, we derived the following estimates of viral suppression proportion among people living with HIV in Kenya.. Because data was not available for 2014, we assume a linear increase in viral suppression from 2013 to 2015. We assume that Kenya will achieve UNAIDS 90-90-90 goal by 2030.

| Year | Female | Male | Reference |
| --- | --- | --- | --- |
| 2005 | 2.9 | 2.2 | ^65,66,69^ |
| 2006 | 6.6 | 4.9 |  |
| 2007 | 9.2 | 6.8 |  |
| 2008 | 12.5 | 9.3 |  |
| 2009 | 23.0 | 19.6 |  |
| 2010 | 31.4 | 26.7 |  |
| 2011 | 35.3 | 30.0 |  |
| 2012 | 38.7 | 32.9 |  |
| 2013 | 44.4 | 37.7 |  |
| 2015 | 44.8 | 38.0 |  |
| 2016 | 54.2 | 46.1 |  |
| 2017 | 63.0 | 53.6 |  |
| 2030 | 72.9 | 72.9 |  |

######

**Figure S2.** Viral suppression proportion among all men and women with HIV in the model (blue), compared to adjusted observed data (red).

### ii. Condoms

Condoms use is initiated in the model in 1995 and stabilized in 2000. Based on data from observational studies and DHS surveys, we assume the condom use varied by sexual risk group, with people in the highest risk group most likely to use condoms.^10,70,71^ We specify that condom use was 12% in the low risk group, 25% in the medium risk group, and 35% in the high risk group. We assume condoms reduce HIV acquisition in both males and females by 80%.^72^ However, we assume no protection against HPV acquisition with condom use.^73-76^

### iii. Circumcision

We assume that circumcised men without HIV have 55% lower risk of HIV acquisition compared to uncircumcised men,^77^ however, their risk for HPV acquisition is not reduced.^78-81^ We also assume no protective effect of circumcision against HPV infection for men with HIV.^82^ In addition, while circumcision does not reduce the risk of HIV transmission to female partners,^83,84^ we assume that women with circumcised, HPV-infected male partner have 23% lower risk of HPV acquisition.^85^

We model medical circumcision beginning in 1960 for 15-19 and 20-24 age groups to account for the traditional practice of male circumcision as a rite of passage for many young men in Kenya.^86^ For this reason, the national prevalence of circumcision was >80% in 2003^87^ before the campaigns to scale up circumcision began in 2008.^88^ We adjusted the circumcision rate so that the proportion of men circumcised matches the coverage levels reported in 2003, 2008-2009, and 2014 DHS surveys (Table S13).^10,87,89^ The prevalence of circumcision among men without HIV between 2000 and 2020 in the model is illustrated in Figure S3. The model does not track circumcision among men with HIV. We assume circumcision coverage scales up to 90% in all ages by 2030.

**Table S13. Proportion of men without HIV who are circumcised.**

| Year | 15-19 | 20-24 | 25-29 | 30-39 | 40-49 | Reference |
| --- | --- | --- | --- | --- | --- | --- |
| 2003 | 71.5 | 89 | 88.3 | 89.3 | 83.7 | ^87^ |
| 2008 | 75.8 | 88.6 | 85.1 | 89.5 | 91.9 | ^89^ |
| 2014 | 87.1 | 96.5 | 94.6 | 93.4 | 91.9 | ^10^ |

######
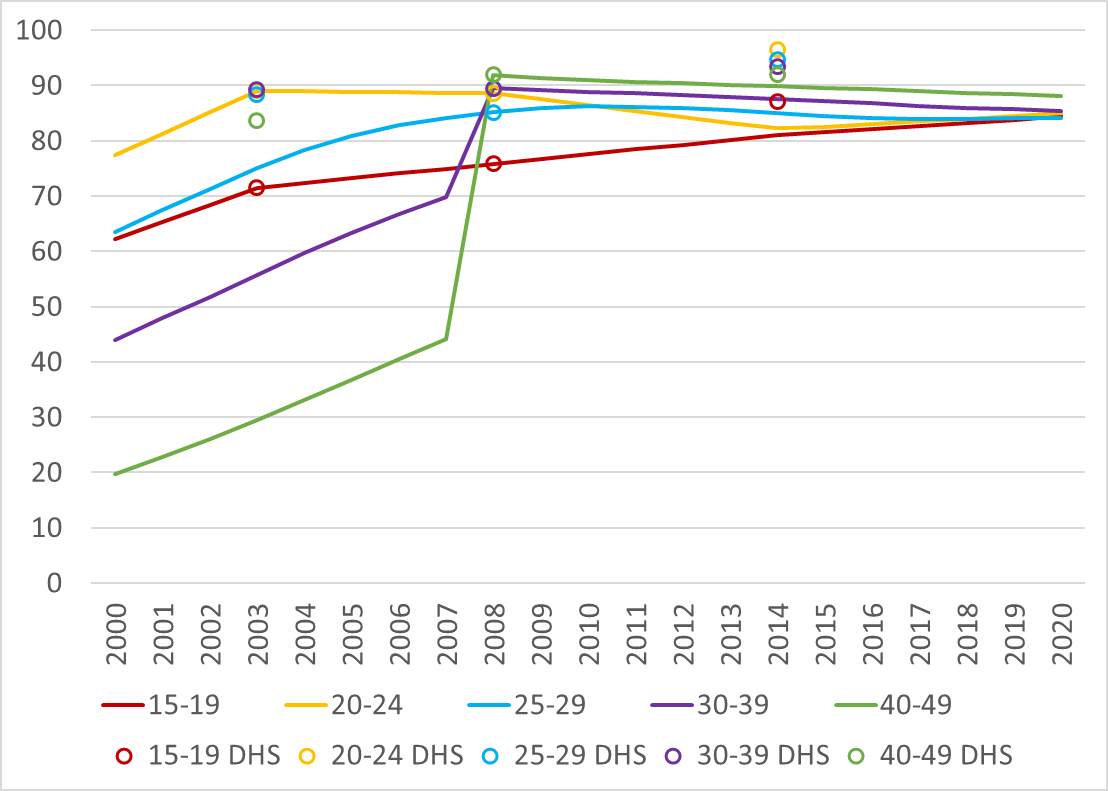


**Figure S3.** Prevalence of male circumcision among men without HIV in the model (lines) compared to DHS estimates for the entire Kenyan male population (circles).

### iv. HPV vaccination

Our model was designed to evaluate the impact of the nonavalent HPV vaccine, in that we group HPV infections into vaccine-targeted types (HPV 16, 18, 31, 33, 45, 52, and 58) and other high-risk HPV types. Under this scheme, the nonavalent vaccine is 100% efficacious against infection with the vaccine-targeted types. We assume that the vaccine provides complete, lifelong protection against covered types. The vaccine is ineffective for persons currently infected with a vaccine-targeted HPV types.

### v. HPV screening and treatment

Beginning in 2000, we model once-per-lifetime cervical cancer screening for women in the age range of 35-39. Although the Kenyan Ministry of Health during this time^90^ recommended screening every 5 years beginning at age 30, observed data suggest low compliance with this schedule.^91^ Informed by consultation with in-country experts, we conservatively assume only one lifetime screen in the model. Based on our analysis of 2014 DHS data, we assume 7.4% screening coverage in women without HIV and 12.3% screening coverage among women with HIV. We assume that women are screened using visual inspection with acetic acid (VIA) with a sensitivity of 62%.^92^ Of the women who test positive, 72% return for triage using colposcopic biopsy.^93^ Of those confirmed to have CIN2-3, we assume 50% are lost to follow up^93^ while the remaining 50% are treated with cryotherapy, which as an efficacy of 97% in women without HIV and 66% in women with HIV.^94,95^ Among women confirmed to have cervical cancer after triage, 40% return for hysterectomy treatment.^93^ These rates reflect challenges with follow-up and retention for cervical cancer prevention and treatment, particularly with a multi-visit screening and treatment approach.

Additionally, evidence suggests that 28% of women treated for CIN2-3 have persistent HPV infection, including cases with residual or recurrent CIN.^96^ Because this estimate is drawn from studies of primarily women without HIV, we used the estimate of 9% treatment failure in women without HIV above to calculate the percent of women who would have persistent HPV with successfully treated lesions (18.5%). Lacking data on differential HPV persistence by HIV status, we assume that HPV persistence after lesion clearance is the same among women living with HIV. Women with a successfully treated lesion who also clear HPV are assumed to develop temporary, partial immunity against reinfection with the same modeled HPV type (vaccine-targeted type vs. other hrHPV).

We assume that hysterectomy is 100% effective at treating cervical cancer. We do not account for treatment or recurrence of late-stage cancers that have spread to other organ systems. After hysterectomy, women are assumed to be infertile, unable to acquire or transmit HPV, and to have no increase in mortality due to their prior cancer status. We do not model symptomatic diagnosis of cervical cancer or treatment with other methods such as radiation or chemotherapy. We assume cervical screening and treatment are equally effective for both vaccine-targeted type and other hrHPV. For women infected with both, their disease status is determined by her most advanced HPV type.

# III. Calibration and validation

## a. Calibration

The initial model parameter values were based on a previously published version of the model fitted to KwaZulu-Natal, South Africa; the calibration method is discussed in detail in the supplemental appendix to the publication.^2^ For this current modeling study, we manually calibrated the parameters to fit to HIV, HPV, and cervical cancer epidemiology in 2000-2020 in Kenya. Our calibration targets are listed in Table S14. We calibrated the parameters related to demographics to match UNPD-estimated population size from 1950-2070. We based sexual behavior parameters on data reported in the 2014 Kenya DHS,^10^ then calibrated these parameters to produce time and age trends in HIV and HPV prevalence that match the observed data. To calibrate sexual behaviors, we assume that people in higher risk groups have more sex partners per year but have fewer coital acts per partnership, reflecting higher partnership formation rate and shorter partnership duration. We calibrate the age-specific number of coital acts per partnership in women. We assume men aged 10-19 have the same number of acts per partnership as women, whereas men aged 20-79 have equal acts to women of the next lowest age group, reflecting age disparities in partnerships.^12^

To reflect higher transmission probability of non-vaccine-targeted types, we calibrate the HPV transmission probability for vaccine-targeted type, then we apply a multiplier on this probability to approximate the transmission probability of non-vaccine-targeted types. We adjust this multiplier so that relative distribution of types matches those observed in studies from sub-Saharan Africa (Table S15). We calibrated HPV and CIN progression parameters to cervical cancer incidence estimates from GLOBOCAN 2012^97^ for Kenya due to a lack of high-quality national-level data. Although there two regional cancer registries in Kenya, their coverage is limited to the urban populations in Nairobi and Eldoret.^98^ As a result, the registries may be underestimating the cancer burden in the country. Moreover, low coverage of cervical cancer screening uptake and incomplete case-finding within the registries’ catchment areas could further contribute to the underestimation of cervical cancer burden. The value for the relative risk of HIV acquisition with HPV infection is randomly selected from a uniform distribution with a minimum of 1.6 and maximum of 2.2.^21^ We ran 100 iterations, and report the median and the interquartile range of outcomes.

**Table S14:** Calibration targets.

|  | **Year** | **Age** | **Value** | **Reference** |
| --- | --- | --- | --- | --- |
| **Total population size** | 2000 | 0-79 | 31890777 | ^3^ |
|  | 2005 | 0-79 | 36547290 |  |
|  | 2010 | 0-79 | 41942714 |  |
|  | 2015 | 0-79 | 47765057 |  |
|  | 2020 | 0-79 | 53627022 |  |
|  | 2025 | 0-79 | 59981315 |  |
|  | 2030 | 0-79 | 66449655 |  |
|  | 2035 | 0-79 | 73026286 |  |
|  | 2040 | 0-79 | 79469672 |  |
|  | 2045 | 0-79 | 85669262 |  |
|  | 2050 | 0-79 | 91575092 |  |
|  | 2055 | 0-79 | 97174763 |  |
|  | 2060 | 0-79 | 102398265 |  |
|  | 2065 | 0-79 | 107170157 |  |
|  | 2070 | 0-79 | 111411341 |  |
| **Population age distribution, men and women combined** | 2010 | 0-9 | 0.31 | ^3^ |
|  |  | 10-19 | 0.23 |  |
|  |  | 20-29 | 0.19 |  |
|  |  | 30-39 | 0.12 |  |
|  |  | 40-49 | 0.07 |  |
|  |  | 50-59 | 0.04 |  |
|  |  | 60-79 | 0.03 |  |
| **Population age distribution, men and women combined** | 2020 | 0-9 | 0.31 | ^3^ |
|  |  | 10-19 | 0.23 |  |
|  |  | 20-29 | 0.19 |  |
|  |  | 30-39 | 0.12 |  |
|  |  | 40-49 | 0.07 |  |
|  |  | 50-59 | 0.04 |  |
|  |  | 60-79 | 0.03 |  |
| **HIV Prevalence, men** | 2003 | 15-19 | 0.004 | ^87^ |
|  |  | 20-24 | 0.024 |  |
|  |  | 25-29 | 0.073 |  |
|  |  | 30-34 | 0.066 |  |
|  |  | 35-39 | 0.084 |  |
|  |  | 40-44 | 0.088 |  |
|  |  | 45-49 | 0.052 |  |
|  |  | 15-49 | 0.046 |  |
|  | 2007 | 15-19 | 0.01 | ^99^ |
|  |  | 20-24 | 0.02 |  |
|  |  | 25-29 | 0.07 |  |
|  |  | 30-34 | 0.09 |  |
|  |  | 35-39 | 0.09 |  |
|  |  | 40-44 | 0.10 |  |
|  |  | 45-49 | 0.06 |  |
|  |  | 15-49 | 0.056 |  |
|  | 2009 | 15-19 | 0.007 | ^89^ |
|  |  | 20-24 | 0.015 |  |
|  |  | 25-29 | 0.065 |  |
|  |  | 30-34 | 0.068 |  |
|  |  | 35-39 | 0.104 |  |
|  |  | 40-44 | 0.057 |  |
|  |  | 45-49 | 0.043 |  |
|  |  | 15-49 | 0.046 |  |
|  | 2012 | 15-19 | 0.009 | ^100^ |
|  |  | 20-24 | 0.013 |  |
|  |  | 25-29 | 0.043 |  |
|  |  | 30-34 | 0.066 |  |
|  |  | 35-39 | 0.05 |  |
|  |  | 40-44 | 0.081 |  |
|  |  | 45-49 | 0.089 |  |
|  |  | 15-49 | 0.044 |  |
| **HIV prevalence, women** | 2003 | 15-19 | 0.030 | ^87^ |
|  |  | 20-24 | 0.090 |  |
|  |  | 25-29 | 0.129 |  |
|  |  | 30-34 | 0.117 |  |
|  |  | 35-39 | 0.118 |  |
|  |  | 40-44 | 0.095 |  |
|  |  | 45-49 | 0.039 |  |
|  |  | 15-49 | 0.087 |  |
|  | 2007 | 15-19 | 0.035 | ^99^ |
|  |  | 20-24 | 0.074 |  |
|  |  | 25-29 | 0.102 |  |
|  |  | 30-34 | 0.133 |  |
|  |  | 35-39 | 0.112 |  |
|  |  | 40-44 | 0.094 |  |
|  |  | 45-49 | 0.088 |  |
|  |  | 15-49 | 0.09 |  |
|  | 2009 | 15-19 | 0.03 | ^89^ |
|  |  | 20-24 | 0.06 |  |
|  |  | 25-29 | 0.10 |  |
|  |  | 30-34 | 0.11 |  |
|  |  | 35-39 | 0.09 |  |
|  |  | 40-44 | 0.14 |  |
|  |  | 45-49 | 0.06 |  |
|  |  | 15-49 | 0.08 |  |
|  | 2012 | 15-19 | 0.011 | ^100^ |
|  |  | 20-24 | 0.046 |  |
|  |  | 25-29 | 0.079 |  |
|  |  | 30-34 | 0.066 |  |
|  |  | 35-39 | 0.123 |  |
|  |  | 40-44 | 0.106 |  |
|  |  | 45-49 | 0.107 |  |
|  |  | 15-49 | 0.069 |  |
| **HPV prevalence, women** **without HIV** | 2005 | 20-24 | 0.29 (0.25-0.52) | ^101,102^ |
|  |  | 25-29 | 0.30 (0.12-0.47) |  |
|  |  | 30-39 | 0.26 (0.13-0.39) |  |
|  |  | 40-49 | 0.24 (0.10-0.39) |  |
| **HPV prevalence, women with HIV** | 2005 | 20-24 | 0.66 (0.58-0.75) |  |
|  |  | 25-29 | 0.67 (0.56-0.79) |  |
|  |  | 30-39 | 0.57 (0.45-0.71) |  |
|  |  | 40-49 | 0.56 (0.43-0.68) |  |
| **Cervical cancer incidence, per 100,00 women** | 2012 | 15-19 | 0.7 | ^97^ |
|  |  | 20-24 | 0.8 |  |
|  |  | 25-29 | 8.0 |  |
|  |  | 30-34 | 21.0 |  |
|  |  | 35-39 | 40.7 |  |
|  |  | 40-44 | 62.7 |  |
|  |  | 45-49 | 83.1 |  |
|  |  | 50-54 | 103.4 |  |
|  |  | 55-59 | 130.3 |  |
|  |  | 60-64 | 150.5 |  |
|  |  | 65-69 | 156.3 |  |
|  |  | 70-74 | 150.7 |  |
|  |  | 75-79 | 133.2 |  |
|  |  | 15-79 | 40.0 |  |

**Table S15.** Relative distribution of HPV types (vaccine-targeted vs non-vaccine-targeted) at each stage of HPV infection and progression.

| **Criteria** | **HPV Type** | **Value** | **Reference** |
| --- | --- | --- | --- |
| **HPV** | Vaccine-targeted | 0.4682 | ^103,104^ |
|  | Non-vaccine-targeted | 0.5318 |  |
| **CIN1** | Vaccine-targeted | 0.5192 | ^103,105^ |
|  | Non-vaccine-targeted | 0.4808 |  |
| **CIN3** | Vaccine-targeted | 0.7371 | ^47,103,106^ |
|  | Non-vaccine-targeted | 0.2629 |  |
| **Cervical cancer** | Vaccine-targeted | 0.8578 | ^46,103,107,108^ |
|  | Non-vaccine-targeted | 0.1422 |  |


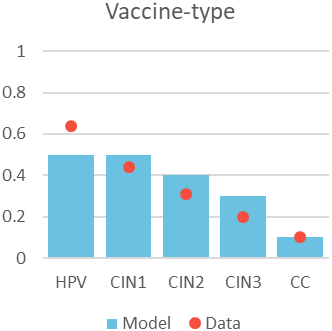

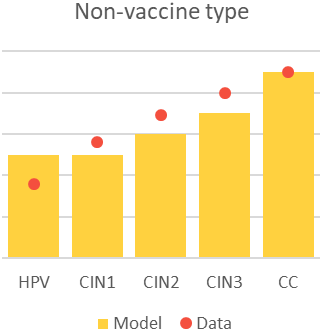


**Figure S4.** Distribution of HPV types at each HPV state in the model compared to data.

## b. Validation

We validated our HIV natural history module to additional time points and data sources (Table S16). We compared our model HIV output to age- and sex-specific HIV prevalence reported in the Population-based HIV Impact Assessment (PHIA) in 2018, which used a similar sampling method as DHS and KAIS surveys.^66^ To validate cervical cancer incidence projection, we compared model output against GLOBOCAN 2020.^109^

**Table S16.** Validation targets.

|  | **Year** | **Age** | **Value** | **Reference** |
| --- | --- | --- | --- | --- |
| **HIV Prevalence, men** | 2018 | 15-19 | 0.005 | ^66^ |
|  |  | 20-24 | 0.006 |  |
|  |  | 25-29 | 0.022 |  |
|  |  | 30-34 | 0.032 |  |
|  |  | 35-39 | 0.043 |  |
|  |  | 40-44 | 0.063 |  |
|  |  | 45-49 | 0.083 |  |
|  |  | 15-49 | 0.031 |  |
| **HIV Prevalence, women** | 2018 | 15-19 | 0.012 | ^66^ |
|  |  | 20-24 | 0.034 |  |
|  |  | 25-29 | 0.060 |  |
|  |  | 30-34 | 0.095 |  |
|  |  | 35-39 | 0.087 |  |
|  |  | 40-44 | 0.119 |  |
|  |  | 45-49 | 0.106 |  |
|  |  | 15-49 | 0.062 |  |
| **Cervical cancer incidence, per 100,00 women** | 2020 | 15-19 | 0.2 | ^109^ |
|  |  | 20-24 | 0.38 |  |
|  |  | 25-29 | 3.5 |  |
|  |  | 30-34 | 14.4 |  |
|  |  | 35-39 | 29.1 |  |
|  |  | 40-44 | 50.4 |  |
|  |  | 45-49 | 72.9 |  |
|  |  | 50-54 | 91.4 |  |
|  |  | 55-59 | 109.7 |  |
|  |  | 60-64 | 122.3 |  |
|  |  | 65-69 | 124.7 |  |
|  |  | 70-74 | 114.1 |  |
|  |  | 75-79 | 80.67 |  |
|  |  | 15-79 | 45.7 |  |

## c. Comparing model output to data

***Demographics***


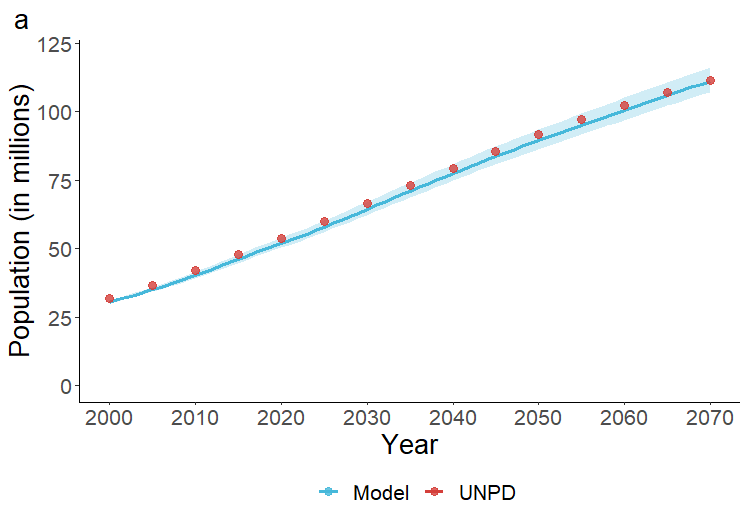

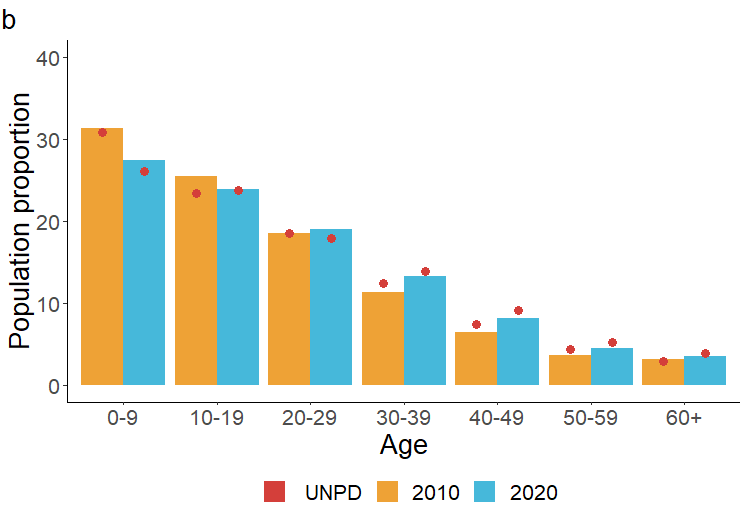


**Figure S5.** A). Model-projected total population size, including men and women, over time (blue line) compared to UNPD estimates for Kenya (red dots). B). Age distribution of model population, in 10-year age groups, in 2010 (yellow) and 2020 (blue) compared to UNPD estimates (red).

***HIV***


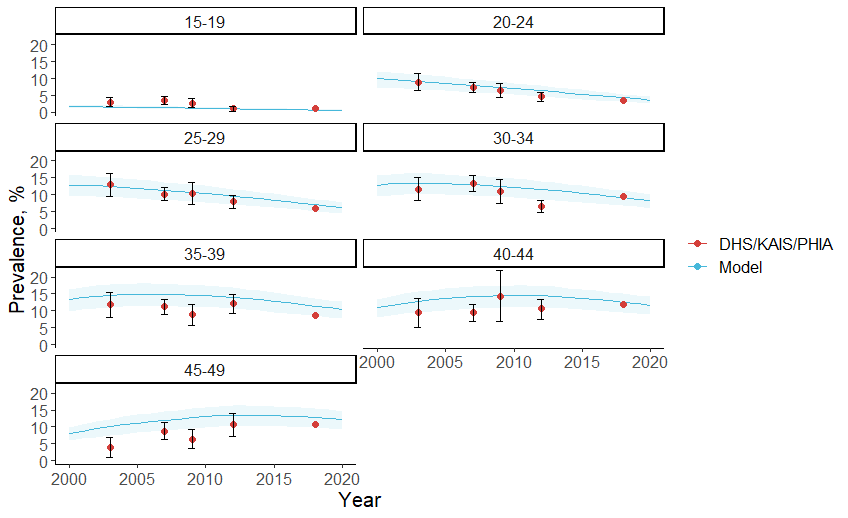


**Figure S6.** Model-estimated HIV prevalence among women by 5-year age groups (blue) compared to age-specific 2003 DHS, 2007 KAIS, 2008-2009 DHS, 2012 KAIS, and 2018 PHIA data for the same age groups (red). We calibrated to 2003, 2007, 2008-2009, and 2012 values and validated against the 2018 values.


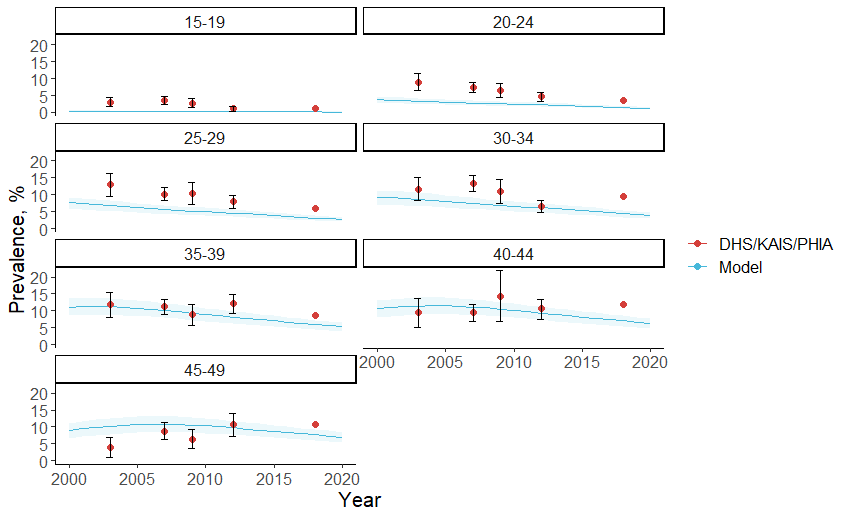


**Figure S7.** Model-estimated HIV prevalence among men by 5-year age groups (blue) compared to age-specific 2003 DHS, 2007 KAIS, 2008-2009 DHS, 2012 KAIS, and 2018 PHIA data for the same age groups (red). We calibrated to 2003, 2007, 2008-2009, and 2012 values and validated against the 2018 values.


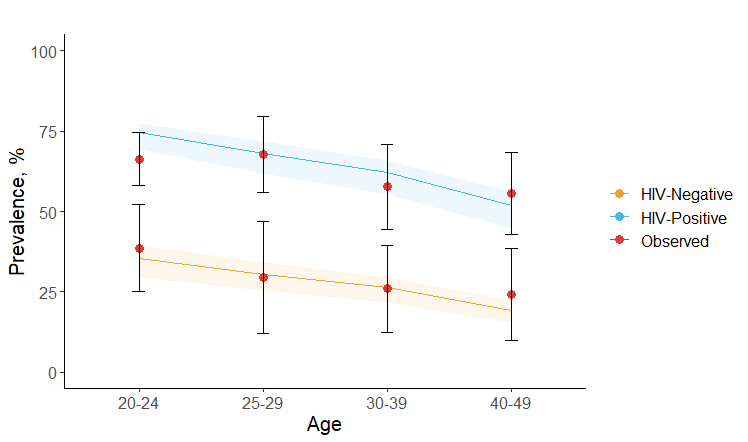


**Figure S8**. Model estimated HPV prevalence by 10-year age group among women without HIV (yellow) and women with HIV (blue) compared to estimates from observational studies.^101,102^


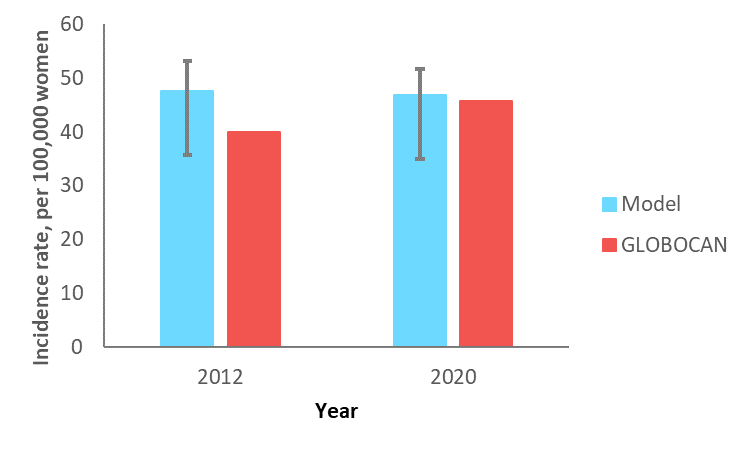


**Figure S9**. Age-standardized cervical cancer incidence rates in 2012 and 2020 for women aged 15-79 (blue) compared to age-standardized estimates for women of the same age from GLOBOCAN (red).


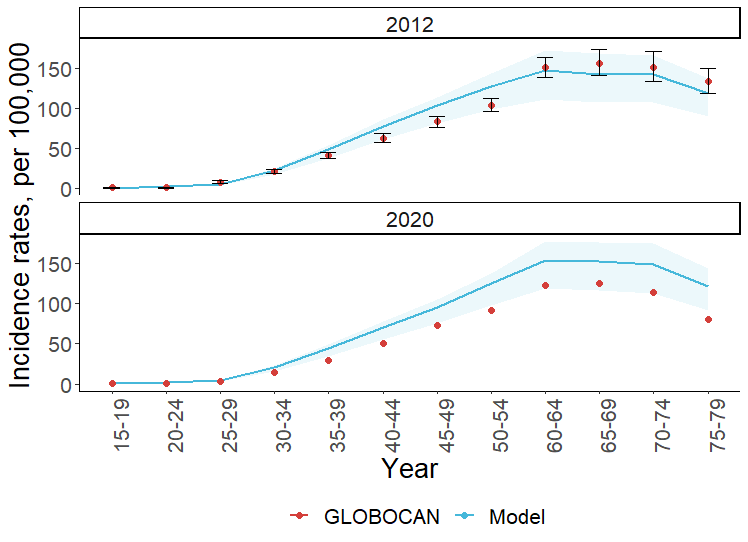


**Figure S10**. Model-estimated age-specific cervical cancer incidence rates (blue), compared to GLOBOCAN estimates (red). We calibrated to 2012 GLOBOCAN data and validated against 2020 GLOBOCAN data.

# IV. Additional results


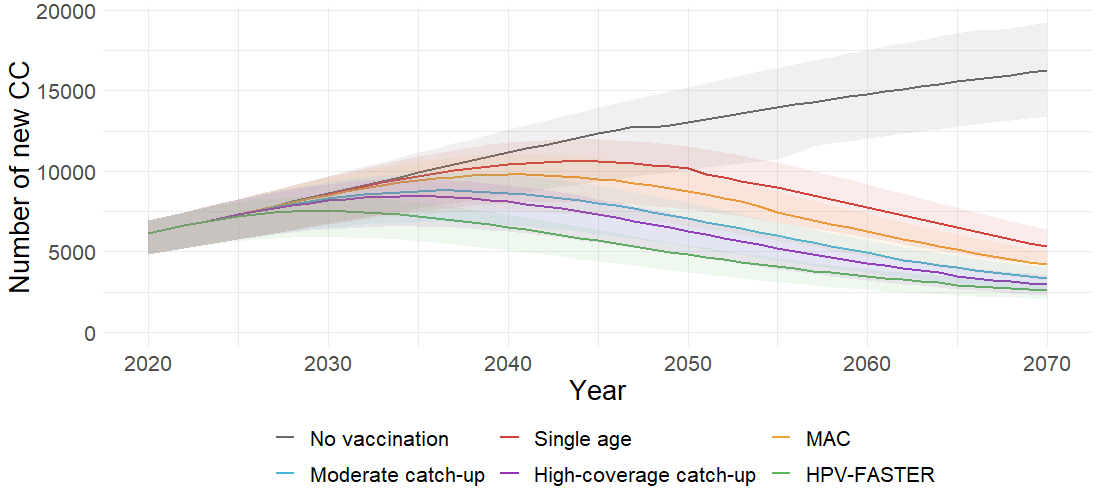


**Figure S11**. Annual number of new cervical cancer cases. Shaded areas represent interquartile ranges of the model estimates.

**Table S17.** The proportion of 15-79 year-old women vaccinated in each scenario.

| Year | Single age | Routine | Moderate catch-up | High-coverage catch-up | HPV-FASTER |
| --- | --- | --- | --- | --- | --- |
| 2030 | 25% | 37% | 48% | 55% | 76% |
| 2040 | 43% | 53% | 61% | 66% | 82% |
| 2050 | 57% | 64% | 71% | 75% | 85% |
| 2060 | 67% | 73% | 78% | 81% | 87% |
| 2070 | 76% | 80% | 83% | 83% | 88% |


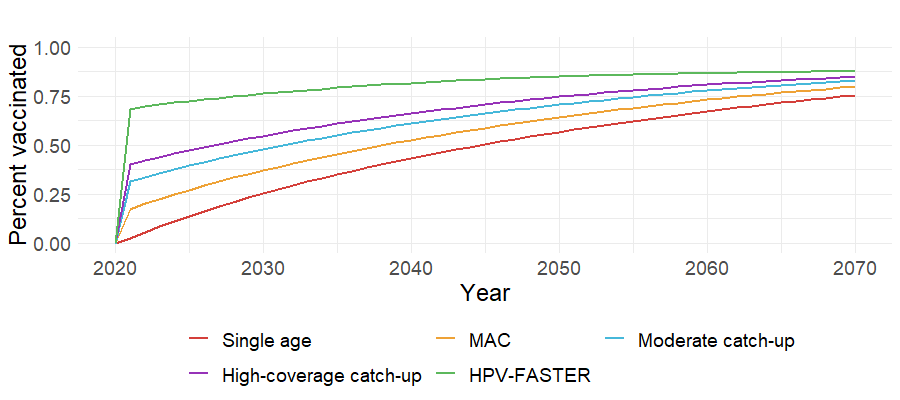


**Figure S12.** The proportion of all girls and women aged 15-79 vaccinated in each scenario.


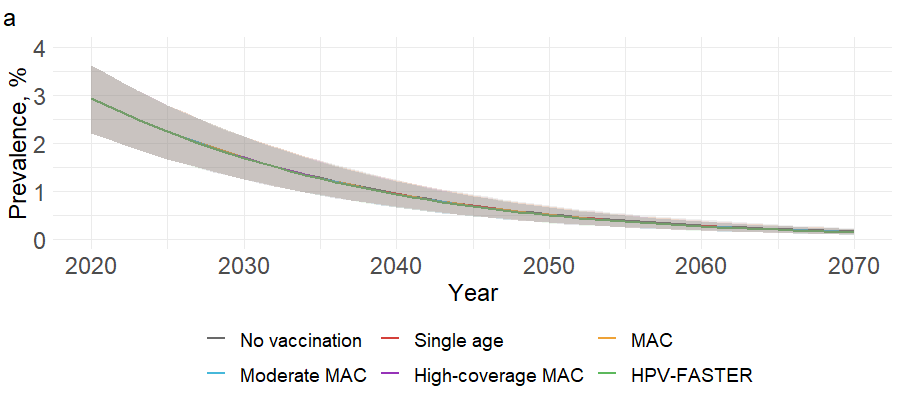

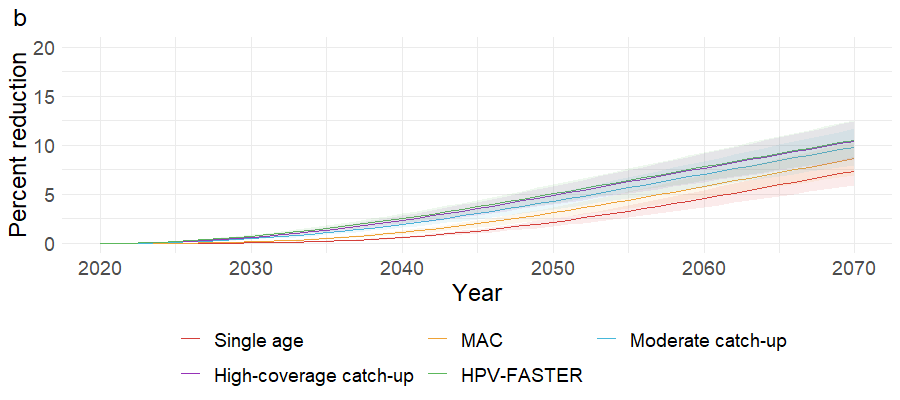


**Figure S13.** HPV vaccination impact on HIV burden in men. Subplot a) HIV prevalence among men over time by scenario. Because HIV prevalence was similar in all scenarios, lines for scenarios overlap almost completely. Subplot b) percent reduction in HIV prevalence relative to no vaccination. Shade areas represent interquartile ranges of the model estimates.

# V. Sensitivity analyses

To assess the effect of ART on HPV vaccination impact on cervical cancer incidence and HIV prevalence, we conduct a sensitivity analysis by varying the ART coverage levels. In our main analysis, we assume that the UNAIDS 90-90-90 goal for HIV treatment in Kenya was achieved by 2030, resulting in 72.9% of people living with HIV virally suppressed by 2030. In the sensitivity analyses, we repeat the main analysis with the following assumptions regarding ART coverage:

1. HIV treatment during the period 2018-2070 is maintained at 2018 levels, which is the latest year for which we have data. Under this assumption, 63% of women living with HIV and 54% of men living with HIV are virally suppressed.
2. UNAIDS 95-95-95 for HIV treatment is achieved by 2030, with 85.7% of both men and women living with HIV achieving viral suppression.

Results from the sensitivity analyses are presented in Tables S19 and S20. Because ART coverage levels in the sensitivity analyses are not drastically different than in the main analysis, projected cervical cancer incidence rates are similar in the sensitivity analyses relative to the main analysis (Table S19). However, compared to the main analysis, relative reduction in cancer incidence due to vaccination was greater when ART coverage was lower and smaller when ART coverage was higher. This shows that HPV vaccine impact on cervical cancer incidence is modified by ART coverage, with the magnitude of effect modification more notable in the near term. Similarly, HPV vaccination impact on HIV burden is also modified by ART coverage (Table S20). Relative to the main analysis, HPV vaccination led to a greater reduction in HIV prevalence and more cumulative cases of HIV averted when ART coverage was lower. Conversely, HPV vaccination resulted in a smaller reduction in HIV prevalence and fewer cases of HIV averted when ART coverage was higher.

**Table S18.** Projected cervical cancer incidence rates and cumulative cervical cancer cases averted in 2050 and 2070 given varying levels of ART coverage.

|  | ART maintained at 2018 level^┼^ | | | UNAIDS 95-95-95 achieved^┼^ | | |
| --- | --- | --- | --- | --- | --- | --- |
|  | **CC incidence, per 100,000** | **% reduction in incidence** | **Cumulative cancer cases averted** | **CC incidence, per 100,000** | **% reduction in incidence** | **Cumulative cancer cases averted** |
| 2050 |  |  |  |  |  |  |
| No vaccination | 31.5 (23.2-37.4) | Reference | Reference | 31.3 (23-37.3) | Reference | Reference |
| Single age cohort | 24.2 (17.9-28.8) | 22.9 (22.7-23.4) | 21435 (18165-25264) | 24.3 (17.9-29) | 22.2 (21.7-22.9) | 21012 (18027-25002) |
| MAC | 21 (15.5-24.9) | 33.2 (32.8-33.8) | 35179 (29366-41240) | 21.3 (15.7-25.2) | 32.1 (31.4-33.0) | 34381 (29145-40855) |
| Moderate catch-up | 17.1 (12.6-20.2) | 45.8 (45.4-46.5) | 57317 (47101-66345) | 17.4 (12.9-20.5) | 44.6 (43.8-45.5) | 56538 (46833-65964) |
| High-coverage catch-up | 15.2 (11.3-17.9) | 51.9 (51.4-52.4) | 68128 (55246-78583) | 15.6 (11.6-18.2) | 50.6 (49.5-51.5) | 67375 (54947-78153) |
| HPV-FASTER | 11.6 (8.8-13.5) | 63.5 (62.9-64.0) | 98042 (76548-113502) | 11.9 (9-13.8) | 62.4 (61.7-63.2) | 98208 (76511-114096) |
| 2070 |  |  |  |  |  |  |
| No vaccination | 27.3 (19.6-32.7) | Reference | Reference | 26.7 (19.4-31.5) | Reference | Reference |
| Single age cohort | 8.5 (6-10.4) | 68.5 (68.2-68.9) | 164014 (138990-195866) | 8.4 (5.9-10.2) | 68.2 (67.6-68.7) | 161730 (136535-194364) |
| MAC | 6.6 (4.8-8.1) | 75.4 (75.0-75.6) | 205949 (172410-245432) | 6.6 (4.7-8) | 74.9 (74.3-75.4) | 202536 (171313-243642) |
| Moderate catch-up | 5.3 (3.9-6.5) | 80.3 (79.9-80.5) | 254194 (210656-300888) | 5.3 (3.8-6.4) | 79.9 (79.3-80.2) | 250507 (209528-299587) |
| High-coverage catch-up | 4.7 (3.5-5.7) | 82.6 (82.2-82.7) | 277751 (227698-327491) | 4.7 (3.5-5.7) | 82.1 (81.7-82.4) | 274006 (226536-326164) |
| HPV-FASTER | 4.2 (3.1-5) | 84.6 (84.1-84.8) | 324577 (260349-379643) | 4.1 (3.1-4.9) | 84.3 (83.9-84.6) | 323216 (259713-379923) |

^┼^If ART coverage is maintained at 2018 levels, 63% of women living with HIV and 54% of men living with HIV are assumed to be virally suppressed. If the UNAIDS 95-95-95 goal for HIV treatment is achieved by 2030, viral suppression level was 85.7% for both men and women.

**Table S19**. Projected HIV prevalence and cumulative cases of HIV averted in 2050 and 2070 given varying levels of ART coverage.

|  | ART maintained at 2018 level^┼^ | | | UNAIDS 95-95-95 achieved^┼^ | | |
| --- | --- | --- | --- | --- | --- | --- |
| Women | **HIV prevalence** | **% reduction in prevalence** | **Cumulative HIV cases averted** | **HIV prevalence** | **% reduction in prevalence** | **Cumulative HIV cases averted** |
| 2050 |  |  |  |  |  |  |
| No vaccination | 1.42 (0.94-1.92) | Reference | Reference | 0.83 (0.57-1.09) | Reference | Reference |
| Single age cohort | 1.37 (0.92-1.84) | 3.3 (2.6-4) | 14466 (9582-24162) | 0.81 (0.57-1.07) | 1.6 (1.2-1.9) | 3789 (2512-6221) |
| MAC | 1.35 (0.91-1.82) | 4.5 (3.6-5.5) | 21240 (14147-35119) | 0.81 (0.56-1.06) | 2.2 (1.7-2.7) | 5816 (3894-9520) |
| Moderate catch-up | 1.33 (0.90-1.79) | 5.9 (4.8-7.1) | 30827 (20813-49884) | 0.8 (0.56-1.05) | 3.1 (2.4-3.8) | 9352 (6336-15030) |
| High-coverage catch-up | 1.32 (0.89-1.78) | 6.6 (5.4-8) | 35663 (24084-57466) | 0.8 (0.56-1.04) | 3.6 (2.8-4.4) | 11159 (7530-17797) |
| HPV-FASTER | 1.31 (0.89-1.77) | 7.1 (5.7-8.5) | 41092 (27590-65136) | 0.79 (0.56-1.04) | 3.8 (2.9-4.6) | 13385 (8978-21012) |
| 2070 |  |  |  |  |  |  |
| No vaccination | 0.58 (0.34-0.87) | Reference | Reference | 0.18 (0.11-0.25) | Reference | Reference |
| Single age cohort | 0.52 (0.32-0.78) | 9.8 (8-11.8) | 36257 (22093-62603) | 0.17 (0.11-0.24) | 5.2 (4.1-6.4) | 6579 (4299-11212) |
| MAC | 0.52 (0.31-0.76) | 11.4 (9.3-13.6) | 46290 (28733-79394) | 0.17 (0.11-0.23) | 6.1 (4.8-7.5) | 9002 (5963-15239) |
| Moderate catch-up | 0.51 (0.31-0.75) | 12.9 (10.5-15.1) | 58858 (37717-99361) | 0.16 (0.11-0.23) | 7.1 (5.6-8.6) | 13013 (8714-21469) |
| High-coverage catch-up | 0.51 (0.31-0.74) | 13.7 (11.1-15.9) | 65118 (42030-109936) | 0.16 (0.11-0.23) | 7.6 (6-9.2) | 15065 (10062-24636) |
| HPV-FASTER | 0.50 (0.31-0.74) | 14.2 (11.5-16.4) | 70740 (46315-119386) | 0.16 (0.11-0.23) | 7.8 (6.2-9.5) | 17577 (11573-28070) |
| Men |  |  |  |  |  |  |
| 2050 |  |  |  |  |  |  |
| No vaccination | 0.65 (0.43-0.88) | Reference | Reference | 0.4 (0.28-0.53) | Reference | Reference |
| Single age cohort | 0.63 (0.42-0.85) | 3.2 (2.6-3.7) | 6700 (4432-10753) | 0.39 (0.28-0.52) | 1.4 (1.1-1.6) | 1636 (984-2624) |
| MAC | 0.62 (0.42-0.84) | 4.4 (3.6-5.3) | 10033 (6718-16077) | 0.39 (0.27-0.52) | 2.1 (1.7-2.5) | 2584 (1709-4131) |
| Moderate catch-up | 0.61 (0.41-0.82) | 5.9 (4.8-6.9) | 14719 (10062-23280) | 0.39 (0.27-0.51) | 3 (2.4-3.6) | 4187 (2929-6625) |
| High-coverage catch-up | 0.61 (0.41-0.82) | 6.7 (5.4-7.8) | 17074 (11731-27011) | 0.39 (0.27-0.51) | 3.5 (2.8-4.1) | 4985 (3499-7904) |
| HPV-FASTER | 0.60 (0.41-0.81) | 7.1 (5.9-8.3) | 19171 (13326-30326) | 0.39 (0.27-0.51) | 3.8 (3.1-4.4) | 5743 (4013-9097) |
| 2070 |  |  |  |  |  |  |
| No vaccination | 0.27 (0.16-0.41) | Reference | Reference | 0.09 (0.06-0.13) | Reference | Reference |
| Single age cohort | 0.25 (0.15-0.37) | 9.9 (8.1-11.7) | 18981 (11438-31412) | 0.09 (0.06-0.12) | 5.3 (4.2-6.3) | 3258 (2081-5323) |
| MAC | 0.24 (0.15-0.36) | 11.4 (9.4-13.5) | 24385 (14973-40053) | 0.08 (0.06-0.12) | 6.2 (4.9-7.5) | 4491 (2925-7321) |
| Moderate catch-up | 0.24 (0.14-0.35) | 13 (10.6-15) | 31048 (19700-50389) | 0.08 (0.05-0.12) | 7.3 (5.8-8.7) | 6384 (4259-10289) |
| High-coverage catch-up | 0.24 (0.14-0.35) | 13.7 (11.2-15.8) | 34404 (21997-55777) | 0.08 (0.05-0.12) | 7.8 (6.2-9.3) | 7327 (4924-11819) |
| HPV-FASTER | 0.24 (0.14-0.35) | 14.2 (11.5-16.3) | 37064 (24057-60054) | 0.08 (0.05-0.12) | 8 (6.4-9.6) | 8134 (5454-13116) |

^┼^If ART coverage is maintained at 2018 levels, 63% of women living with HIV and 54% of men living with HIV are assumed to be virally suppressed. If the UNAIDS 95-95-95 goal for HIV treatment is achieved by 2030, viral suppression level was 85.7% for both men and women.

# VI. Differential equations

We use a system of differential equations to estimate changes in population and infection dynamics over each time step. We split the full system of equations into topic-based modules and solve each iteratively using a 4^th^-order Runge-Kutta numerical solver in MATLAB. The order is largely historical and based on the work of (Tan et al., 2018)^2^. The modules are:

1. HPV natural history
   1. Progression and clearance of HPV
   2. Progression and regression of precancerous lesions
   3. Development and progression of cervical cancer
   4. Cervical cancer- associated mortality
2. Cervical cancer screening and treatment
   1. Screening
   2. Treatment
3. HPV and HIV transmission
   1. Heterosexual mixing by gender, age, and risk group
   2. Partnership adjustment
   3. HPV infection by type
   4. HIV infection
4. HIV natural history and treatment
   1. CD4 progression
   2. Viral load progression
   3. ART initiation, discontinuation, and scale-up by CD4 count
   4. HIV-associated mortality
5. Demography
   1. Births
   2. Mother-to-child HIV transmission
   3. Aging and risk-group redistribution
   4. Natural deaths
6. Male circumcision
7. HPV vaccination
   1. School-based regimen
   2. Catch-up regimen

Throughout each simulation, we track population demographics and the number of persons with infection, with progressed disease, or with preventative or therapeutic treatment. We describe these states $X_{g,a,r}^{d,v,h,s,x,p}(t)$ with the following indices (using 1-based indexing):

| **Index** | **Description** | **Values** |
| --- | --- | --- |
| *d* | HIV disease state, CD4 count, circumcision status, and ART status | 1. HIV-negative, uncircumcised 2. HIV-negative, circumcised 3. HIV-positive, acute infection 4. HIV-positive, CD4 > 500 cells/µL 5. HIV-positive, CD4 350-500 cells/µL 6. HIV-positive, CD4 200-350 cells/µL 7. HIV-positive, CD4 ≤ 200 cells/µL 8. HIV-positive, on ART |
| *v* | HIV viral load | 1. If ( 2 ˂ *d* ˂ 8 ), Acute infection; if ( *d* = 1,2 ), HIV-negative: VL = 0.0 2. Asymptomatic: VL = 3.0-4.5 log_10_ 3. Pre-AIDS symptomatic: VL = 4.0-5.5 log_10_ 4. AIDS: VL = 5.5-7.0 log_10_ 5. Late-stage 6. On ART and virally suppressed: VL = 0.0 |
| *h* | Vaccine-type HPV precancer or disease state | 1. Susceptible 2. Infected 3. CIN1 4. CIN2 5. CIN3 6. Cervical cancer or hysterectomy 7. Immune |
| *s* | Non-vaccine type HPV precancer or disease state | 1. Susceptible 2. Infected 3. CIN1 4. CIN2 5. CIN3 6. Cervical cancer or hysterectomy 7. Immune |
| *x* | Cervical cancer or hysterectomy status | 1. If ( *h* = 6 or *s* = 6 ), Cervical cancer, local; else, no cancer or hysterectomy 2. Cervical cancer, regional 3. Cervical cancer, distant 4. Hysterectomy |
| *p* | Vaccination and screening history | 1. Non-vaccinated, non-screened 2. Vaccinated 3. Screened 4. Vaccinated and screened |
| *g* | Gender | 1. Male 2. Female |
| *a* | Age | 1. 0-4 2. 5-9 3. 10-14 4. 15-19 5. 20-24 6. 25-29 7. 30-34 8. 35-39 9. 40-44 10. 45-49 11. 50-54 12. 55-59 13. 60-64 14. 65-69 15. 70-74 16. 75-79 |
| *r* | Risk | 1. Low risk 2. Medium risk 3. High risk |

## a. Demography

| **Equation variables** | |
| --- | --- |
| $\gamma_{a}^{d}$(t) | The annual fertility rate for females by age *a* and HIV disease stage *d*.  Women ages 15-49 bear children. |
| $\eta\left( t \right)$ | The proportion of births from HIV-positive females that result in vertical transmission. |
| $b\_s\left( t \right)$ | Number of births by women without HIV and women on ART. |
| $b\_i\left( t \right)$ | Number of births by women living with HIV. |
| $b_{g,a,r}^{d,1,h,s,x,p}(t)$ | Number of infant births of HIV disease stage *d* and gender *g.*  We assume an equal gender ratio at birth of 1:1, that all newborns are born as low risk, no vertical transmission of HPV, and that if HIV is vertically transmitted, that infected newborns are born into the acute stage of HIV. |
| $\phi_{g,a,r}$ | Distribution of sexual risk *r* by gender *g* and age *a.* (Currently, the risk distribution derived from male partner data is used for both males and females for simplicity.) |
| ${\mu\_bkrd}_{g,a}(t)$ | Annual background mortality rate by gender *g* and age *a.* |

###### Fertility

The number of births by HIV status of the mother are calculated as:

*HIV-negative women and women on ART*

$$b\_s\left( t \right)=\sum_{h=1}^{7} \sum_{s=1}^{7} \sum_{x=1}^{3} \sum_{p=1}^{4} \sum_{a=4}^{10} \sum_{r=1}^{3} \left[ \gamma_{a}^{1}\left( t \right)\cdot X_{g,a,r}^{1,1,h,s,x,p}\left( t \right)+\gamma_{a}^{8}\left( t \right)\cdot X_{g,a,r}^{8,6,h,s,x,p}\left( t \right) \right]$$

*Women living with HIV*

$$b\_i\left( t \right)=\sum_{d=3}^{7} \sum_{v=1}^{5} \sum_{h=1}^{7} \sum_{s=1}^{7} \sum_{x=1}^{3} \sum_{p=1}^{4} \sum_{a=4}^{10} \sum_{r=1}^{3} \left[ \gamma_{a}^{d}\left( t \right)\cdot X_{g,a,r}^{d,v,h,s,x,p}(t) \right]$$

We then compute the number of births by gender and HIV status of the infant as:

*HIV-negative, uncircumcised births*

*For h = s = x = p = a = r = 1,*

$$b_{g,a,r}^{1,1,h,s,x,p}\left( t \right)=0.5 \left( b\_s\left( t \right)+\left( 1-\eta\left( t \right) \right)b\_i\left( t \right) \right)$$

else,

$$b_{g,a,r}^{1,1,h,s,x,p}\left( t \right)=0$$

*HIV-positive births*

*For h = s = x = p = a = r = 1,*

$$b_{g,a,r}^{3,1,h,s,x,p}\left( t \right)=0.5 \eta\left( t \right)\cdot b\_i(t)$$

else,

$$b_{g,a,r}^{3,1,h,s,x,p}\left( t \right)=0$$

###### Aging

To age the population, one-fifth of each compartment moves to the next age group while maintaining the same gender, disease state, and sexual risk distribution $\phi_{g,a,r}$:

$$\frac{dX_{g,1,r}^{d,v,h,s,x,p}(t)}{dt}=-\frac{1}{5}\sum_{r=1}^{3} X_{g,1,r}^{d,v,h,s,x,p}\left( t \right) \cdot\phi_{g,a,r} (for a=1)$$

$$\frac{dX_{g,a,r}^{d,v,h,s,x,p}(t)}{dt}=-\frac{1}{5}\sum_{r=1}^{3} X_{g,a,r}^{d,v,h,s,x,p}\left( t \right) \cdot\phi_{g,a,r}+\frac{1}{5}\sum_{r=1}^{3} X_{g,a-1,r}^{d,v,h,s,x,p}\left( t \right) \cdot\phi_{g,a-1,r} (for a\neq1)$$

Upon aging to the next five-year group, individuals are re-distributed into the closest unfilled risk group to match observed data on the age distribution of low, medium, and high-risk individuals.

###### Mortality

We compute the number of deaths due to background mortality as:

$$\frac{dX_{g,a,r}^{d,v,h,s,x,p}(t)}{dt}=-{\mu\_bkrd}_{g,a}\left( t \right)\cdot X_{g,a,r}^{d,v,h,s,x,p}\left( t \right)$$

## b. Sexual Behavior

###### Mixing matrix

| **Equation variables** | |
| --- | --- |
| $c_{g,a,r}$ | Number of partners a person has per year of gender *g,* age *a,* and sexual-risk group *r* (ie. the partner exchange rate, or contact rate). |
| $\epsilon_{a}$ | Mixing parameter by age *a.*  We assume a mixing pattern that is partially random and partially off-diagonal (0 < $\epsilon_{a}$ < 1), where ($\epsilon_{a}=0)$ indicates completely off-diagonal mixing, and ($\epsilon_{a}=1)$ indicates completely random mixing. |
| $\epsilon_{r}$ | Mixing parameter by sexual-risk group *r.*  We assume a mixing pattern that is partially random and partially on-diagonal (0 < $\epsilon_{r}$ < 1), where ($\epsilon_{r}=0)$ indicates completely on-diagonal mixing, and ($\epsilon_{r}=1)$ indicates completely random mixing. |
| $\delta_{g,a,a^{'}}$ | Mixing pattern by age.  In completely non-random mixing by age, females are most likely to form partnerships with males of the next oldest age group*.* We represent this pattern using on off-diagonal matrix.  For males (*g* = 1) of age *a* mixing with females of age *a’*:  = 0.3 if ($a=a^{'}$)  = 0.7 if ($a=a^{'}+1$)  except for the following (correct for no sexual activity before age group 3):  = 0.0 if ($a=a^{'}=1$)  = 0.0 if ($a=$ 2) and (*a’* = 1)  = 0.0 if ($a=$2) and (*a’* = 2)  = 0.0 if ($a=$ 3) and (*a’* = 2)  For females (*g* = 2) of age *a* mixing with males of age *a’*:  = 0.3 if ($a=a^{'}$)  = 0.7 if ($a=a^{'}-1$)  except for the following (correct for no sexual activity before age group 3):  = 0.0 if ($a=a^{'}=$1)  = 0.0 if ($a=$1) and (*a’* = 2)  = 0.0 if ($a=a^{'}=$2)  = 0.0 if ($a=$2) and (*a’* = 3) |
| $\delta_{r,r^{'}}$ | Mixing pattern by risk.  Completely non-random mixing by risk confines sexual encounters to individuals within the same risk group. We represent this pattern using an identity matrix.  = 1.0 if ($r=r^{'}$)  = 0.0 if ($r\neq r^{'}$) |

For a person of gender *g,* age *a,* and sexual-risk group *r,* we use the mixing matrix $\rho_{g,a,a^{'},r,r^{'}}(t)$ to describe the proportion of sexual partners that come from age group *a’* and sexual-risk group *r’*. We assume that mixing is partially random and partially designated by a mixing pattern $\delta_{g,a,a^{'}}$ or $\delta_{r,r^{'}}$. The overall mixing matrix is therefore a weighted average of random mixing proportional to the number of available partnerships of each group, and mixing among groups with similar characteristics. Although an off-diagonal mixing pattern results in the first and last ages groups (ages 10-14 and 75-79) having fewer than 100% of their partnerships, these age groups have relatively few partnerships and contribute marginally to overall infection transmission.

$$\rho_{g,a,a^{'},r,r^{'}}\left( t \right)=\left( \epsilon_{a}\cdot\frac{\sum_{r^{'}=1}^{3} \left( c_{g^{'},a^{'},r^{'}}\cdot\sum_{d^{'}=1}^{8} \sum_{v^{'}=1}^{6} \sum_{h^{'}=1}^{7} \sum_{s^{'}=1}^{7} \sum_{x^{'}=1}^{4} \sum_{p^{'}=1}^{4} X_{g',a',r'}^{d',v',h',s',x',p'}\left( t \right) \right)}{\sum_{a^{'}=1}^{16} \sum_{r^{'}=1}^{3} \left( c_{g^{'},a^{'},r^{'}}\cdot\sum_{d^{'}=1}^{8} \sum_{v^{'}=1}^{6} \sum_{h^{'}=1}^{7} \sum_{s^{'}=1}^{7} \sum_{x^{'}=1}^{4} \sum_{p^{'}=1}^{4} X_{g',a',r'}^{d',v',h',s',x',p'}\left( t \right) \right)}+\left( 1-\epsilon_{a} \right)\delta_{g,a,a^{'}} \right)\cdot\left( \epsilon_{r}\cdot\frac{\left( c_{g^{'},a^{'},r^{'}}\cdot\sum_{d^{'}=1}^{8} \sum_{v^{'}=1}^{6} \sum_{h^{'}=1}^{7} \sum_{s^{'}=1}^{7} \sum_{x^{'}=1}^{4} \sum_{p^{'}=1}^{4} X_{g',a',r'}^{d',v',h',s',x',p'}\left( t \right) \right)}{\sum_{r^{'}=1}^{3} \left( c_{g^{'},a^{'},r^{'}}\cdot\sum_{d^{'}=1}^{8} \sum_{v^{'}=1}^{6} \sum_{h^{'}=1}^{7} \sum_{s^{'}=1}^{7} \sum_{x^{'}=1}^{4} \sum_{p^{'}=1}^{4} X_{g',a',r'}^{d',v',h',s',x',p'}\left( t \right) \right)}+\left( 1-\epsilon_{r} \right)\delta_{g,r,r^{'}} \right)$$

###### Rate of partner change

| **Equation variables** | |
| --- | --- |
| $c_{g,a,r}$ | Number of partners a person has per year of gender *g,* age *a,* and sexual-risk group *r* (ie. the partner exchange rate, or contact rate).  We assume zero partnerships for individuals below the age of sexual debut (age 10). |
| $\theta$ | Gender influence on contact rate adjustment.  We assume an adjusted contact rate equally driven by rates reported by males and females ($\theta$= 0.5), where ($\theta$ = 0) when completely female-driven, and ($\theta$ = 1) when completely male-driven. |
| $\rho_{g,a,a^{'},r,r^{'}}\left( t \right)$ | Mixing matrix for a person of gender *g,* age *a,* and sexual-risk group *r* that describes the proportion of sexual partners that come from age group *a’* and sexual-risk group *r’.*  We assume a solely heterosexual population and therefore that all contacts are with the opposite gender. |

Bias in observed data leads to contact rates $c_{g,a,r}$ that, when assuming solely heterosexual contact, are inconsistent between males and females. We account for this variability by using an adjusted contact rate $c_{g,a,a^{'},r,r^{'}}^{*}(t)$ that ensures that the number of partnerships of males of age *a* and risk group *r* with females of age *a’* and risk group *r’* equals the number of partnerships of females of age *a* and risk group *r* with males of age *a’* and risk group *r’*.

We first calculate the discrepancy between reported male and female contacts as:

$$B_{a,a^{'},r,r^{'}}\left( t \right)=\frac{c_{1,a,r}\cdot\rho_{1,a,a^{'},r,r^{'}}\left( t \right)\cdot\sum_{d^{'}=1}^{8} \sum_{v^{'}=1}^{6} \sum_{h^{'}=1}^{7} \sum_{s^{'}=1}^{7} \sum_{x^{'}=1}^{4} \sum_{p^{'}=1}^{4} X_{1,a',r'}^{d',v',h',s',x',p'}\left( t \right)}{c_{2,a,r}\cdot\rho_{2,a,a^{'},r,r^{'}}\left( t \right)\cdot\sum_{d=1}^{8} \sum_{v=1}^{6} \sum_{h=1}^{7} \sum_{s=1}^{7} \sum_{x=1}^{4} \sum_{p=1}^{4} X_{2,a,r}^{d,v,h,s,x,p}\left( t \right)}$$

We then compute the adjusted contact rate for females as:

$$c_{2,a,a^{'},r,r^{'}}^{*}\left( t \right)=c_{2,a,r}\cdot\rho_{2,a,a^{'},r,r^{'}}\left( t \right)\cdot{B_{a,a^{'},r,r^{'}}\left( t \right)}^{\theta}\cdot\left( \frac{\sum_{d^{'}=1}^{8} \sum_{v^{'}=1}^{6} \sum_{h^{'}=1}^{7} \sum_{s^{'}=1}^{7} \sum_{x^{'}=1}^{4} \sum_{p^{'}=1}^{4} X_{1,a',r'}^{d',v',h',s',x',p'}\left( t \right)}{\sum_{d=1}^{8} \sum_{v=1}^{6} \sum_{h=1}^{7} \sum_{s=1}^{7} \sum_{x=1}^{4} \sum_{p=1}^{4} X_{2,a,r}^{d,v,h,s,x,p}\left( t \right)} \right)^{-\left( 1-\theta\right)}$$

and for males, an adjusted contact rate of:

$$c_{1,a,a^{'},r,r^{'}}^{*}(t)=c_{1,a,r}\cdot\rho_{1,a,a^{'},r,r^{'}}\left( t \right)\cdot{B_{a,a^{'},r,r^{'}}\left( t \right)}^{-\left( 1-\theta\right)}\cdot\left( \frac{\sum_{d^{'}=1}^{8} \sum_{v^{'}=1}^{6} \sum_{h^{'}=1}^{7} \sum_{s^{'}=1}^{7} \sum_{x^{'}=1}^{4} \sum_{p^{'}=1}^{4} X_{1,a',r'}^{d',v',h',s',x',p'}\left( t \right)}{\sum_{d=1}^{8} \sum_{v=1}^{6} \sum_{h=1}^{7} \sum_{s=1}^{7} \sum_{x=1}^{4} \sum_{p=1}^{4} X_{2,a,r}^{d,v,h,s,x,p}\left( t \right)} \right)^{\theta}$$

## c. Transmission Probabilities

###### Per-partnership probability of transmission

| **Equation variables** | |
| --- | --- |
| $A_{g,a,r}$ | Number of acts per partnership of gender *g,* age *a,* and sexual-risk group *r*.  We assume zero acts for individuals below the age of sexual debut (age 10). |
| ${\chi\_HIV}_{g}^{v^{'},x^{'}}$ | Per-act probability of HIV transmission to a person of gender *g* based on the viral load *v’* of the HIV-positive partner*.*  We assume the probability of female-to-male HIV transmission is equal to the probability of male-to-female transmission across all viral load stages (${\chi\_HIV}_{1}^{v^{'}}= {\chi\_HIV}_{2}^{v^{'}}$). We reduce HIV per-act transmission as a proxy for decreased sexual activity during late-stage HIV (*v’* = 5), regional or distant cervical cancer (*x’* = 2 or *x’* = 3), or hysterectomy (*x’* = 4). |
| ${\chi\_HPV}_{g}^{v^{'},x^{'}}$ | Per-act probability of HPV transmission to a person of gender *g*.  We assume the per-act probability of HPV transmission is the same for vaccine-type and non-vaccine-type HPV and across all stages of pre-cancer or cervical cancer. We reduce HPV per-act transmission as a proxy for decreased sexual activity during late-stage HIV (*v’* = 5) or regional or distant cervical cancer (*x’* = 2 or *x’* = 3). We assume no HPV transmission after hysterectomy (*x’* = 4). |

The per-partnership probability of HIV transmission ${\beta\_HIV}_{g,a,r}^{v^{'},x^{'}}$ is the cumulative risk of acquiring HIV from all sexual acts with a partner. This quantity depends on the per-act probability of HIV transmission and the number of acts per partnership.

We calculate the per-partnership probability of HIV transmission to a male partner:

$${\beta\_HIV}_{1,a,r}^{v^{'},x^{'}}=1-{(1-{\chi\_HIV}_{1}^{v^{'},x^{'}})}^{A_{1,a,r}}$$

Similarly, the per-partnership probability of HIV transmission to a female partner:

$${\beta\_HIV}_{2,a,r}^{v^{'},x^{'}}=1-{(1-{\chi\_HIV}_{2}^{v^{'},x^{'}})}^{A_{2,a,r}}$$

Likewise, the per-partnership probability of HPV transmission ${\beta\_HPV}_{g,a,r}^{v^{'},x^{'}}$ depends on the per-act probability of HPV transmission and the number of acts per partnership.

We calculate the per-partnership probability of HPV transmission to a male partner:

$${\beta\_HPV}_{1,a,r}^{v^{'},x^{'}}=1-{(1-{\chi\_HPV}_{1}^{v^{'},x^{'}})}^{A_{1,a,r}}$$

Similarly, the per-partnership probability of HPV transmission to a female partner:

$${\beta\_HPV}_{2,a,r}^{v^{'},x^{'}}=1-{(1-{\chi\_HPV}_{2}^{v^{'},x^{'}})}^{A_{2,a,r}}$$

###### Force of infection

| **Equation variables** | |
| --- | --- |
| $c_{g,a,a^{'},r,r^{'}}^{*}(t)$ | Adjusted yearly contact rate for persons of gender *g,* age *a,* and risk group *r,* with persons of the opposite gender, age *a’,* and risk group *r’.* |
| ${\beta\_HIV}_{g,a,r}^{v^{'},x^{'}}$ | Annual per-partnership probability of HIV transmission from a HIV-positive person with viral load *v’* and cervical cancer stage *x’* to a HIV-susceptible partner with gender *g,* age *a,* and risk group *r.* |
| ${\beta\_HPV}_{g,a,r}^{v^{'},x^{'}}$ | Annual per-partnership probability of HPV transmission from a HPV-infected person with viral load *v’* and cervical cancer stage *x’* to a HPV-susceptible partner with gender *g,* age *a,* and risk group *r.* |

The force of infection represents the cumulative risk of acquiring HIV or HPV from all possible partners, and depends on the adjusted contact rate, the per-partnership probability of transmission, and the proportion of sexually active persons who are HIV- or HPV-infected.

The force of infection ${\lambda\_HIV}_{g,a,r}(t)$ determines HIV disease transmission:

$${\lambda\_HIV}_{g,a,r}(t)=\sum_{a^{'}=1}^{16} \sum_{r^{'}=1}^{3} \left( c_{g,a,a^{'},r,r^{'}}^{*}(t)\cdot\frac{-\sum_{v^{'}=1}^{6} \sum_{x^{'}=1}^{4} ln(1-{\beta\_HIV}_{g,a,r}^{v^{'},x^{'}})\cdot\sum_{d^{'}=3}^{8} \sum_{h^{'}=1}^{7} \sum_{s^{'}=1}^{7} \sum_{p^{'}=1}^{4} X_{g',a',r'}^{d',v',h',s',x',p'}\left( t \right)}{\sum_{d^{'}=1}^{8} \sum_{v^{'}=1}^{6} \sum_{h^{'}=1}^{7} \sum_{s^{'}=1}^{7} \sum_{x^{'}=1}^{4} \sum_{p^{'}=1}^{4} X_{g',a',r'}^{d',v',h',s',x',p'}\left( t \right)} \right)$$

Similarly, the force of infection ${\lambda\_vHPV}_{g,a,r}(t)$ determines vaccine-type HPV transmission:

$${\lambda\_vHPV}_{g,a,r}(t)=\sum_{a^{'}=1}^{16} \sum_{r^{'}=1}^{3} \left( c_{g,a,a^{'},r,r^{'}}^{*}(t)\cdot\frac{-\sum_{v^{'}=1}^{6} \sum_{x^{'}=1}^{4} ln(1-{\beta\_HPV}_{g,a,r}^{v^{'},x^{'}})\cdot\sum_{d^{'}=1}^{8} \sum_{h^{'}=2}^{6} \sum_{s^{'}=1}^{7} \sum_{p^{'}=1}^{4} X_{g',a',r'}^{d',v',h',s',x',p'}\left( t \right)}{\sum_{d^{'}=1}^{8} \sum_{v^{'}=1}^{6} \sum_{h^{'}=1}^{7} \sum_{s^{'}=1}^{7} \sum_{x^{'}=1}^{4} \sum_{p^{'}=1}^{4} X_{g',a',r'}^{d',v',h',s',x',p'}\left( t \right)} \right)$$

and $\lambda{\_nvHPV}_{g,a,r}(t)$ defines non-vaccine-type HPV transmission:

$$\lambda{\_nvHPV}_{g,a,r}\left( t \right)=\sum_{a^{'}=1}^{16} \sum_{r^{'}=1}^{3} \left( c_{g,a,a^{'},r,r^{'}}^{*}(t)\cdot\frac{-\sum_{v^{'}=1}^{6} \sum_{x^{'}=1}^{4} ln(1-{\beta\_HPV}_{g,a,r}^{v^{'},x^{'}})\cdot\sum_{d^{'}=1}^{8} \sum_{h^{'}=1}^{7} \sum_{s^{'}=2}^{6} \sum_{p^{'}=1}^{4} X_{g',a',r'}^{d',v',h',s',x',p'}\left( t \right)}{\sum_{d^{'}=1}^{8} \sum_{v^{'}=1}^{6} \sum_{h^{'}=1}^{7} \sum_{s^{'}=1}^{7} \sum_{x^{'}=1}^{4} \sum_{p^{'}=1}^{4} X_{g',a',r'}^{d',v',h',s',x',p'}\left( t \right)} \right)$$

## d. Natural History and Interventions

###### HIV

| **Equation variables** | |
| --- | --- |
| ${\mu\_HIV}_{g,a}^{d}$ | Annual HIV-associated mortality rate by gender *g*, age *a*, and HIV disease stage *d* for (3 ≤ *d* ≤ 8)*.* |
| ${\lambda\_HIV}_{g,a,r}(t)$ | Force of HIV infection fo persons without HIV by gender *g,* age *a,* and risk *r*. |
| ${\rho\_HIV}_{g}$ | Reduction in HIV acquisition due to circumcision by gender.  Only males receive circumcision (${\rho\_HIV}_{2}=1$). |
| ${\psi\_HIV}_{g}$ | Reduction in HIV acquisition due to population-level condom use by gender. |
| $\omega^{d}$ | The rate of progressing from HIV stage *d* to stage *d + 1,* for (3 ≤ *d* ≤ 7)*.* |
| $l^{d}$ | The rate of progressing from viral load stage *v* to *v + 1,* for (1 ≤ *v* ≤ 5). |
| $P_{g,a}(t)$ | The proportion of HIV-negative persons of gender *g* and age *a* that are circumcised.  Only males receive circumcision ($P_{2,a}(t)$ = 0). |
| $A_{g,a}^{d}(t)$ | The proportion of persons living with HIV of disease stage *d,* gender *g,* and age *a* that initiate ART. |
| $\sigma_{g,a,r}^{d,v}(t)$ | The proportion of persons who discontinue ART based on the recent distribution of persons initiating ART by gender *g*, age *a,* risk *r,* disease *d,* and viral *v*. |

We calculate changes in HIV status and HIV stage defined by CD4 count, viral load, and treatment status. The population without HIV can acquire HIV after sexual debut with a force of infection reduced by circumcision in males and condom use by either gender. We only track circumcision among men without HIV. Individuals with HIV infection experience HIV-associated mortality, CD4 and viral load stage progression, and ART initiation and discontinuation. CD4 and viral load stage are not tracked among persons on treatment.

*HIV-negative, uncircumcised*

$$\frac{dX_{g,a,r}^{1,1,h,s,x,p}\left( t \right)}{dt}=-\left( {\psi\_HIV}_{g}{\cdot\lambda\_HIV}_{g,a,r}\left( t \right) +P_{g,a}\left( t \right) \right)X_{g,a,r}^{1,1,h,s,x,p}\left( t \right)$$

*HIV-negative, circumcised*

$$\frac{dX_{g,a,r}^{2,1,h,s,x,p}\left( t \right)}{dt}=P_{g,a}\left( t \right) \cdot X_{g,a,r}^{1,1,h,s,x,p}\left( t \right)$$

$$- \left( {\psi\_HIV}_{g}\cdot{\rho\_HIV}_{g}\cdot{\lambda\_HIV}_{g,a,r}(t) \right) X_{g,a,r}^{2,1,h,s,x,p}\left( t \right)$$

*HIV-positive, acute infection*

$$\frac{dX_{g,a,r}^{3,1,h,s,x,p}\left( t \right)}{dt}={\psi\_HIV}_{g}{\cdot\lambda\_HIV}_{g,a,r}(t)\cdot X_{g,a,r}^{1,1,h,s,x,p}\left( t \right)$$

$$+ {\psi\_HIV}_{g}\cdot{\rho\_HIV}_{g}\cdot{\lambda\_HIV}_{g,a,r}\left( t \right)\cdot X_{g,a,r}^{2,1,h,s,x,p}\left( t \right)+\sigma_{g,a,r}^{3,1}(t){\cdot X}_{g,a,r}^{8,6,h,s,x,p}\left( t \right) -\left( {\mu\_HIV}_{g,a}^{3}+\omega^{3}+A_{g,a}^{3}(t) \right)X_{g,a,r}^{3,1,h,s,x,p}\left( t \right)$$

*HIV-positive, CD4 > 500 cells/µL*

$$\frac{dX_{g,a,r}^{4,v,h,s,x,p}\left( t \right)}{dt}= \omega^{3}{\cdot X}_{g,a,r}^{3,v,h,s,x,p}\left( t \right)+ l^{v-1}{\cdot X}_{g,a,r}^{4,v-1,h,s,x,p}\left( t \right) +\sigma_{g,a,r}^{4,v}(t)\cdot X_{g,a,r}^{8,6,h,s,x,p}\left( t \right)-\left( {\mu\_HIV}_{g,a}^{4}+\omega^{4}+l^{v}+A_{g,a}^{4}(t) \right)X_{g,a,r}^{4,v,h,s,x,p}\left( t \right)$$

*HIV-positive, CD4 350-500 cells/µL*

$$\frac{dX_{g,a,r}^{5,v,h,s,x,p}\left( t \right)}{dt}= \omega^{4}\cdot X_{g,a,r}^{4,v,h,s,x,p}\left( t \right)+ l^{v-1}\cdot X_{g,a,r}^{5,v-1,h,s,x,p}\left( t \right) +\sigma_{g,a,r}^{5,v}(t){\cdot X}_{g,a,r}^{8,6,h,s,x,p}\left( t \right)-\left( {\mu\_HIV}_{g,a}^{5}+\omega^{5}+l^{v}+A_{g,a}^{5}(t) \right)X_{g,a,r}^{5,v,h,s,x,p}\left( t \right)$$

*HIV-positive, CD4 200-350 cells/µL*

$$\frac{dX_{g,a,r}^{6,v,h,s,x,p}\left( t \right)}{dt}= \omega^{5}{\cdot X}_{g,a,r}^{5,v,h,s,x,p}\left( t \right)+ l^{v-1}\cdot X_{g,a,r}^{6,v-1,h,s,x,p}\left( t \right) +\sigma_{g,a,r}^{6,v}(t){\cdot X}_{g,a,r}^{8,6,h,s,x,p}\left( t \right)-\left( {\mu\_HIV}_{g,a}^{6}+\omega^{6}+l^{v}+A_{g,a}^{6}(t) \right)X_{g,a,r}^{6,v,h,s,x,p}\left( t \right)$$

*HIV-positive, CD4 ≤ 200 cells/µL*

$$\frac{dX_{g,a,r}^{7,v,h,s,x,p}\left( t \right)}{dt}= \omega^{6}{\cdot X}_{g,a,r}^{6,v,h,s,x,p}\left( t \right)+ l^{v-1}{\cdot X}_{g,a,r}^{7,v-1,h,s,x,p}\left( t \right) +\sigma_{g,a,r}^{7,v}(t)\cdot X_{g,a,r}^{8,6,h,s,x,p}\left( t \right)-\left( {\mu\_HIV}_{g,a}^{7}+\omega^{7}+l^{v}+A_{g,a}^{7}(t) \right)X_{g,a,r}^{7,v,h,s,x,p}\left( t \right)$$

*HIV-positive, on ART*

$$\frac{dX_{g,a,r}^{8,6,h,s,x,p}\left( t \right)}{dt}= \sum_{d=3}^{7} \sum_{v=1}^{5} \left( A_{g,a}^{d}\left( t \right)\cdot X_{g,a,r}^{d,v,h,s,x,p}\left( t \right)-\sigma_{g,a,r}^{d,v}{\cdot X}_{g,a,r}^{8,6,h,s,x,p}\left( t \right) \right)$$

###### HPV

| **Equation variables** | |
| --- | --- |
| ${\mu\_HPV}_{g}^{d,h,s,x}$ | Annual cervical cancer-associated mortality rate by gender *g*, HIV disease stage *d*, vaccine-type HPV stage *h,* non-vaccine-type HPV stage *s,* and cervical cancer stage *x* for (1 ≤ *x* ≤ 3).  Only females have cervical cancer-associated mortality (${\mu\_HPV}_{1}^{d,h,s,x}$ = 0) and only when (*h* = 6 or *s* = 6). |
| ${\lambda\_vHPV}_{g,a,r}(t)$ | Force of vaccine-type HPV infection for susceptible persons of gender *g,* age *a,* and risk *r*. |
| ${\lambda\_nvHPV}_{g,a,r}(t)$ | Force of non-vaccine-type HPV infection for susceptible persons of gender *g,* age *a,* and risk *r*. |
| $ж_{d}$ | HPV acquisition risk multiplier for HIV-positive individuals with CD4 count (4 ≤ *d* ≤ 7)*.* |
| ${\rho\_HPV}_{g}$ | HPV acquisition reduction multiplier due to circumcision by gender.  Only males receive circumcision (${\rho\_HPV}_{2}=1$). |
| ${\psi\_HPV}_{g}$ | HPV acquisition reduction multiplier due to population-level condom use by gender. |
| $\xi_{g,a}$ | HPV acquisition reduction multiplier by gender and age for individuals with type-specific natural immunity.  Only females temporarily develop partial natural immunity ($\xi_{1,a}=0$). Older women develop stronger natural immunity than young girls. |
| $\phi_{a}$ | Vaccine-type HPV acquisition reduction multiplier by age for vaccinated individuals.  We assume life-long protection with vaccination ($\phi_{a}$ is equivalent for all vaccinated ages). |
| ${k\_v}_{g,a}^{h,h^{'}}$ | Transition rate of progressing or regressing from vaccine-type HPV precancer or disease stage *h* to stage *h’*.  Only women develop precancerous lesions and cervical cancer (${k\_v}_{1,a}^{h,h^{'}}$= 0 except for HPV clearance when *h* = 2 and *h’* = 1). |
| ${k\_nv}_{g,a}^{s,s^{'}}$ | Transition rate of progressing or regressing from non-vaccine-type HPV precancer or disease stage *s* to stage *s’*.  Only women develop precancerous lesions and cervical cancer (${k\_v}_{1,a}^{s,s^{'}}$= 0 except for HPV clearance when *s* = 2 and s*’* = 1). |
| $r_{g}$ | Rate of waning type-specific natural immunity.  Only females temporarily develop partial natural immunity ($r_{1}=0$). |
| $\varphi_{g}^{h,s,x,x^{'}}$ | Progression rate of cervical cancer from stage *x* to stage *x’*.  Only women develop cervical cancer ($\varphi_{1}^{h,s,x,x^{'}}=0$) and ($\varphi_{2}^{h,s,x,x^{'}}>0$ only when *h* or *s* = 6) |
| ${\zeta\_v}^{d,h,h^{'}}$ | Transition rate multiplier for HIV-positive individuals progressing or regressing from vaccine-type precancer or disease stage *h* to stage *h’* with CD4 count *d.*  Transition rate multipliers for HIV-positive individuals are the same for vaccine-type and non-vaccine-type HPV (${\zeta\_v}^{d,h,h^{'}}= {\zeta\_nv}^{d,s,s^{'}}$ when *h* = *s* and *h’* = *s’*)*.* |
| ${\zeta\_nv}^{d,s,s^{'}}$ | Transition rate multiplier for HIV-positive individuals progressing or regressing from non-vaccine-type precancer or disease stage *s* to stage *s’* with gender *g* and CD4 count *d.*  Transition rate multipliers for HIV-positive individuals are the same for vaccine-type and non-vaccine-type HPV (${\zeta\_v}^{d,h,h^{'}}= {\zeta\_nv}^{d,s,s^{'}}$ when *h* = *s* and *h’* = *s’*)*.* |
| $\mathcal{l}_{g}$ | Additional multiplier for clearance of vaccine or non-vaccine-type HPV infection.  Only applied to males ($\mathcal{l}_{2}=1$). |
| $V_{g,a}^{d}$ | The proportion of persons with HIV disease status *d,* gender *g,* and age *a* vaccinated*.* |

###### Vaccine-targeted HPV types and precancer equations

*Male susceptible*

$$\frac{dX_{1,a,r}^{d,v,1,s,1,1}\left( t \right)}{dt}= {\mathcal{l}_{1}\cdot\zeta\_v}^{d,2,1}\cdot{k\_v}_{1,a}^{2,1}\cdot X_{1,a,r}^{d,v,2,s,1,1}\left( t \right) -\left( ж_{d}\cdot{\rho\_HPV}_{1}\cdot{\psi\_HPV}_{1}{\cdot\lambda\_vHPV}_{1,a,r}(t)+V_{1,a}^{d} \right)X_{1,a,r}^{d,v,1,s,1,1}\left( t \right)$$

*Male HPV-infected*

$$\frac{dX_{1,a,r}^{d,v,2,s,1,1}\left( t \right)}{dt}={ж_{d}\cdot{\rho_{HPV}}_{1}\cdot{\psi_{HPV}}_{1}{\cdot\lambda_{vHPV}}_{1,a,r}\left( t \right)\cdot X}_{1,a,r}^{d,v,1,s,1,1}\left( t \right)$$

$$- {\mathcal{l}_{1}\cdot\zeta\_v}^{d,2,1}\cdot{k\_v}_{1,a}^{2,1}\cdot X_{1,a,r}^{d,v,2,s,1,1}\left( t \right)$$

*Male susceptible, vaccinated*

$$\frac{dX_{1,a,r}^{d,v,1,s,1,2}\left( t \right)}{dt}= V_{1,a}^{d}\cdot X_{1,a,r}^{d,v,1,s,1,1}\left( t \right)+ {\mathcal{l}_{1}\cdot\zeta\_v}^{d,2,1}\cdot{k\_v}_{1,a}^{2,1}\cdot X_{1,a,r}^{d,v,2,s,1,2}\left( t \right) -{\phi_{a}\cdotж_{d}\cdot{\rho\_HPV}_{1}\cdot{\psi\_HPV}_{1}{\cdot\lambda\_vHPV}_{1,a,r}(t)\cdot X}_{1,a,r}^{d,v,1,s,1,2}\left( t \right)$$

*Male HPV-infected, vaccinated*

$$\frac{dX_{1,a,r}^{d,v,2,s,1,2}\left( t \right)}{dt}={{\phi_{a}\cdotж}_{d}\cdot{\rho_{HPV}}_{1}\cdot{\psi_{HPV}}_{1}{\cdot\lambda_{vHPV}}_{1,a,r}\left( t \right)\cdot X}_{1,a,r}^{d,v,1,s,1,2}\left( t \right)$$

$$- {\mathcal{l}_{1}\cdot\zeta\_v}^{d,2,1}\cdot{k\_v}_{1,a}^{2,1}\cdot X_{1,a,r}^{d,v,2,s,1,2}\left( t \right)$$

*Female susceptible*

$$\frac{dX_{2,a,r}^{d,v,1,s,x,[1,3]}\left( t \right)}{dt}= {\zeta\_v}^{d,7,1}\cdot r_{2}\cdot X_{2,a,r}^{d,v,7,s,x,[1,3]}\left( t \right) -\left( ж_{d}\cdot{\psi\_HPV}_{2}{\cdot\lambda\_vHPV}_{2,a,r}(t)+V_{2,a}^{d}+{\mu\_HPV}_{2}^{d,1,s,x} \right)X_{2,a,r}^{d,v,1,s,x,[1,3]}\left( t \right)$$

*Female immune*

$$\frac{dX_{2,a,r}^{d,v,7,s,x,[1,3]}\left( t \right)}{dt}= V_{2,a}^{d}\cdot X_{2,a,r}^{d,v,7,s,x,[1,3]}\left( t \right)+{\zeta\_v}^{d,2,7}\cdot{k\_v}_{2,a}^{2,7}\cdot X_{2,a,r}^{d,v,2,s,x,[1,3]}\left( t \right) -\left( {\zeta\_v}^{d,7,1}\cdot{k\_v}_{2,a}^{7,1}+\xi_{2,a}{\cdotж}_{d}\cdot{\psi\_HPV}_{2}{\cdot\lambda\_vHPV}_{2,a,r}(t)+V_{2,a}^{d}+{\mu\_HPV}_{2}^{d,7,s,x} \right)X_{2,a,r}^{d,v,7,s,x,[1,3]}\left( t \right)$$

*Female HPV-infected*

$$\frac{dX_{2,a,r}^{d,v,2,s,x,[1,3]}\left( t \right)}{dt}= {\zeta\_v}^{d,3,2}\cdot{k\_v}_{2,a}^{3,2}\cdot X_{2,a,r}^{d,v,3,s,x,\left[ 1,3 \right]}\left( t \right) +ж_{d}\cdot{\psi\_HPV}_{2}{\cdot\lambda\_vHPV}_{2,a,r}\left( t \right)\cdot X_{2,a,r}^{d,v,1,s,x,\left[ 1,3 \right]}\left( t \right)+\xi_{2,a}{\cdotж}_{d}\cdot{\psi\_HPV}_{2}{\cdot\lambda\_vHPV}_{2,a,r}\left( t \right)\cdot X_{2,a,r}^{d,v,7,s,x,\left[ 1,3 \right]}\left( t \right)-\left( {\zeta\_v}^{d,2,7}\cdot{k\_v}_{2,a}^{2,7}+{\zeta\_v}^{d,2,3}\cdot{k\_v}_{2,a}^{2,3}+{\mu\_HPV}_{2}^{d,2,s,x} \right)X_{2,a,r}^{d,v,2,s,x,[1,3]}\left( t \right)$$

*Female susceptible, vaccinated*

$$\frac{dX_{2,a,r}^{d,v,1,s,x,[2,4]}\left( t \right)}{dt}= {\zeta\_v}^{d,7,1}\cdot r_{2}\cdot X_{2,a,r}^{d,v,7,s,x,\left[ 2,4 \right]}\left( t \right)+V_{2,a}^{d}\cdot X_{2,a,r}^{d,v,1,s,x,[1,3]}\left( t \right)-\left( \phi_{a}\cdotж_{d}\cdot{\psi\_HPV}_{2}{\cdot\lambda\_vHPV}_{2,a,r}(t)+{\mu\_HPV}_{2}^{d,1,s,x} \right)X_{2,a,r}^{d,v,1,s,x,[2,4]}\left( t \right)$$

*Female immune, vaccinated*

$$\frac{dX_{2,a,r}^{d,v,7,s,x,[2,4]}\left( t \right)}{dt}= {\zeta\_v}^{d,2,7}\cdot{k\_v}_{2,a}^{2,7}\cdot X_{2,a,r}^{d,v,2,s,x,\left[ 2,4 \right]}\left( t \right)+V_{2,a}^{d}\cdot X_{2,a,r}^{d,v,7,s,x,[1,3]}\left( t \right) -\left( {\zeta\_v}^{d,7,1}\cdot{k\_v}_{2,a}^{7,1}+{\phi_{a}\cdot\xi}_{2,a}{\cdotж}_{d}\cdot{\psi\_HPV}_{2}{\cdot\lambda\_vHPV}_{2,a,r}(t)+{\mu\_HPV}_{2}^{d,7,s,x} \right)X_{2,a,r}^{d,v,7,s,x,[2,4]}\left( t \right)$$

*Female HPV-infected, vaccinated*

$$\frac{dX_{2,a,r}^{d,v,2,s,x,[2,4]}\left( t \right)}{dt}= {\zeta\_v}^{d,3,2}\cdot{k\_v}_{2,a}^{3,2}\cdot X_{2,a,r}^{d,v,3,s,x,\left[ 2,4 \right]}\left( t \right) +{\phi_{a}\cdotж}_{d}\cdot{\psi\_HPV}_{2}{\cdot\lambda\_vHPV}_{2,a,r}\left( t \right)\cdot X_{2,a,r}^{d,v,2,s,x,\left[ 2,4 \right]}\left( t \right)+{\phi_{a}\cdot\xi}_{2,a}{\cdotж}_{d}\cdot{\psi\_HPV}_{2}{\cdot\lambda\_vHPV}_{2,a,r}\left( t \right)\cdot X_{2,a,r}^{d,v,7,s,x,\left[ 2,4 \right]}\left( t \right)-\left( {\zeta\_v}^{d,2,7}\cdot{k\_v}_{2,a}^{2,7}+{\zeta\_v}^{d,2,3}\cdot{k\_v}_{2,a}^{2,3}+{\mu\_HPV}_{2}^{d,2,s,x} \right)X_{2,a,r}^{d,v,2,s,x,[2,4]}\left( t \right)$$

*Female CIN1*

$$\frac{dX_{2,a,r}^{d,v,3,s,x,p}\left( t \right)}{dt}= {\zeta\_v}^{d,4,3}\cdot{k\_v}_{2,a}^{4,3}\cdot X_{2,a,r}^{d,v,4,s,x,p}\left( t \right) +{\zeta\_v}^{d,2,3}\cdot{k\_v}_{2,a}^{2,3}\cdot X_{2,a,r}^{d,v,2,s,x,p}\left( t \right)-\left( {\zeta\_v}^{d,3,4}\cdot{k\_v}_{2,a}^{3,4}+{\zeta\_v}^{d,3,2}\cdot{k\_v}_{2,a}^{3,2}+{\mu\_HPV}_{2}^{d,3,s,x} \right)X_{2,a,r}^{d,v,3,s,x,p}\left( t \right)$$

*Female CIN2*

$$\frac{dX_{2,a,r}^{d,v,4,s,x,p}\left( t \right)}{dt}= {\zeta\_v}^{d,5,4}\cdot{k\_v}_{2,a}^{5,4}\cdot X_{2,a,r}^{d,v,5,s,x,p}\left( t \right) +{\zeta\_v}^{d,3,4}\cdot{k\_v}_{2,a}^{3,4}\cdot X_{2,a,r}^{d,v,3,s,x,p}\left( t \right)-\left( {\zeta\_v}^{d,4,5}\cdot{k\_v}_{2,a}^{4,5}+{\zeta\_v}^{d,4,3}\cdot{k\_v}_{2,a}^{4,3}+{\mu\_HPV}_{2}^{d,4,s,x} \right)X_{2,a,r}^{d,v,4,s,x,p}\left( t \right)$$

*Female CIN3*

$$\frac{dX_{2,a,r}^{d,v,5,s,x,p}\left( t \right)}{dt}= {\zeta\_v}^{d,4,5}\cdot{k\_v}_{2,a}^{4,5}\cdot X_{2,a,r}^{d,v,4,s,x,p}\left( t \right)-\left( {\zeta\_v}^{d,5,6}\cdot{k\_v}_{2,a}^{5,6}+{\zeta\_v}^{d,5,4}\cdot{k\_v}_{2,a}^{5,4}+{\mu\_HPV}_{2}^{d,5,s,x} \right)X_{2,a,r}^{d,v,5,s,x,p}\left( t \right)$$

###### Non-vaccine-targeted HPV types and precancer equations

The non-vaccine-type HPV and precancer equations follow the same pattern as the vaccine-type HPV equations with a few updates. All values of *s* equal the values of *h* in the vaccine-type equations, and *h* equals any value. Vaccination does not depend on non-vaccine-type HPV infection status.

###### Cervical cancer equations

*Female cervical cancer, local*

*(where h=6)*

$$\frac{dX_{2,a,r}^{d,v,6,s,x,p}\left( t \right)}{dt}= {\zeta\_v}^{d,5,6}\cdot{k\_v}_{2,a}^{5,6}\cdot X_{2,a,r}^{d,v,5,s,x,p}\left( t \right)$$

*(where s=6)*

$$\frac{dX_{2,a,r}^{d,v,h,6,x,p}\left( t \right)}{dt}= {\zeta\_v}^{d,5,6}\cdot{k\_v}_{2,a}^{5,6}\cdot X_{2,a,r}^{d,v,h,6,x,p}\left( t \right)$$

*(where h=6 or s=6)*

$$\frac{dX_{2,a,r}^{d,v,h,s,1,p}\left( t \right)}{dt}=-\left( \varphi_{2}^{h,s,1,2}+{\mu\_HPV}_{2}^{d,h,s,1} \right)X_{2,a,r}^{d,v,h,s,1,p}\left( t \right)$$

*Female cervical cancer, regional (where h=6 or s=6)*

$$\frac{dX_{2,a,r}^{d,v,h,s,2,p}\left( t \right)}{dt}= \varphi_{2}^{h,s,1,2}\cdot X_{2,a,r}^{d,v,h,s,1,p}\left( t \right)-\left( \varphi_{2}^{h,s,2,3}+{\mu\_HPV}_{2}^{d,h,s,2} \right)X_{2,a,r}^{d,v,h,s,2,p}\left( t \right)$$

*Female cervical cancer, distant (where h=6 or s=6)*

$$\frac{dX_{2,a,r}^{d,v,h,s,3,p}\left( t \right)}{dt}= \varphi_{2}^{h,s,2,3}\cdot X_{2,a,r}^{d,v,h,s,2,p}\left( t \right)-\left( {\mu\_HPV}_{2}^{d,h,s,3} \right)X_{2,a,r}^{d,v,h,s,3,p}\left( t \right)$$

# VII. References

1. UNAIDS. UNAIDS Data Geneva, Switzerland: Joint United Nations Programme on HIV/AIDS; 2020.

2. Tan N, Sharma M, Winer R, Galloway D, Rees H, Barnabas RV. Model-estimated effectiveness of single dose 9-valent HPV vaccination for HIV-positive and HIV-negative females in South Africa. Vaccine. 2018;36(32 Pt A):4830-6.

3. United Nations Department of Economic and Social Affairs. World Population Prospects Geneva, Switzerland: United Nations; 2019 [Available from: <https://population.un.org/wpp/Download/Standard/Population/>.

4. Kenya National Bureau of Statistics. The 2009 Kenya Population and Housing Census. Nairobi, Kenya: Kenya National Bureau of Statistics; 2010.

5. Kenya National Bureau of Statistics. The 2019 Kenya Population and Housing Census. Nairobi, Kenya: Kenya National Bureau of Statistics; 2020.

6. Ross A, Van der Paal L, Lubega R, Mayanja BN, Shafer LA, Whitworth J. HIV-1 disease progression and fertility: the incidence of recognized pregnancy and pregnancy outcome in Uganda. AIDS. 2004;18(5):799-804.

7. Tweya H, Feldacker C, Breeze E, Jahn A, Haddad LB, Ben-Smith A, et al. Incidence of pregnancy among women accessing antiretroviral therapy in urban Malawi: a retrospective cohort study. AIDS and behavior. 2013;17(2):471-8.

8. Newell ML, Coovadia H, Cortina-Borja M, Rollins N, Gaillard P, Dabis F, et al. Mortality of infected and uninfected infants born to HIV-infected mothers in Africa: a pooled analysis. Lancet. 2004;364(9441):1236-43.

9. Badri M, Lawn SD, Wood R. Short-term risk of AIDS or death in people infected with HIV-1 before antiretroviral therapy in South Africa: a longitudinal study. Lancet. 2006;368(9543):1254-9.

10. Kenya National Bureau of S, Ministry of HK, National ACCK, Kenya Medical Research I, National Council for P, Development/Kenya. Kenya Demographic and Health Survey 2014. Rockville, MD, USA; 2015.

11. Garnett GP, Anderson RM. Factors controlling the spread of HIV in heterosexual communities in developing countries: patterns of mixing between different age and sexual activity classes. Philosophical transactions of the Royal Society of London Series B, Biological sciences. 1993;342(1300):137-59.

12. Ferry B, Caraël M, Buvé A, Auvert B, Laourou M, Kanhonou L, et al. Comparison of key parameters of sexual behaviour in four African urban populations with different levels of HIV infection. AIDS. 2001;15:S41-S50.

13. Datta P, Embree JE, Kreiss JK, Ndinya-Achola JO, Braddick M, Temmerman M, et al. Mother-to-child transmission of human immunodeficiency virus type 1: report from the Nairobi Study. J Infect Dis. 1994;170(5):1134-40.

14. Waruru A, Achia TNO, Muttai H, Ng'ang'a L, Zielinski-Gutierrez E, Ochanda B, et al. Spatial-temporal trend for mother-to-child transmission of HIV up to infancy and during pre-Option B+ in western Kenya, 2007-13. PeerJ. 2018;6:e4427.

15. Quinn TC, Wawer MJ, Sewankambo N, Serwadda D, Li C, Wabwire-Mangen F, et al. Viral load and heterosexual transmission of human immunodeficiency virus type 1. Rakai Project Study Group. N Engl J Med. 2000;342(13):921-9.

16. Hubert JB, Burgard M, Dussaix E, Tamalet C, Deveau C, Le Chenadec J, et al. Natural history of serum HIV-1 RNA levels in 330 patients with a known date of infection. The SEROCO Study Group. AIDS. 2000;14(2):123-31.

17. Lingappa JR, Hughes JP, Wang RS, Baeten JM, Celum C, Gray GE, et al. Estimating the impact of plasma HIV-1 RNA reductions on heterosexual HIV-1 transmission risk. PLoS One. 2010;5(9):e12598.

18. Hollingsworth TD, Anderson RM, Fraser C. HIV-1 transmission, by stage of infection. The Journal of infectious diseases. 2008;198(5):687-93.

19. Gray RH, Wawer MJ, Brookmeyer R, Sewankambo NK, Serwadda D, Wabwire-Mangen F, et al. Probability of HIV-1 transmission per coital act in monogamous, heterosexual, HIV-1-discordant couples in Rakai, Uganda. Lancet (London, England). 2001;357(9263):1149-53.

20. Boily MC, Baggaley RF, Wang L, Masse B, White RG, Hayes RJ, et al. Heterosexual risk of HIV-1 infection per sexual act: systematic review and meta-analysis of observational studies. The Lancet Infectious diseases. 2009;9(2):118-29.

21. Houlihan CF, Larke NL, Watson-Jones D, Smith-McCune KK, Shiboski S, Gravitt PE, et al. Human papillomavirus infection and increased risk of HIV acquisition. A systematic review and meta-analysis. AIDS (London, England). 2012;26(17):2211-22.

22. Looker KJ, Rönn MM, Brock PM, Brisson M, Drolet M, Mayaud P, et al. Evidence of synergistic relationships between HIV and Human Papillomavirus (HPV): systematic reviews and meta-analyses of longitudinal studies of HPV acquisition and clearance by HIV status, and of HIV acquisition by HPV status. J Int AIDS Soc. 2018;21(6):e25110-e.

23. Auvert B, Marais D, Lissouba P, Zarca K, Ramjee G, Williamson A-L. High-risk human papillomavirus is associated with HIV acquisition among South African female sex workers. Infect Dis Obstet Gynecol. 2011;2011:692012-.

24. Averbach SH, Gravitt PE, Nowak RG, Celentano DD, Dunbar MS, Morrison CS, et al. The association between cervical human papillomavirus infection and HIV acquisition among women in Zimbabwe. AIDS. 2010;24(7):1035-42.

25. Nowak RG, Gravitt PE, Morrison CS, Gange SJ, Kwok C, Oliver AE, et al. Increases in human papillomavirus detection during early HIV infection among women in Zimbabwe. J Infect Dis. 2011;203(8):1182-91.

26. Low AJ, Clayton T, Konate I, Nagot N, Ouedraogo A, Huet C, et al. Genital warts and infection with human immunodeficiency virus in high-risk women in Burkina Faso: a longitudinal study. BMC Infect Dis. 2011;11:20-.

27. Myer L, Denny L, Wright TC, Kuhn L. Prospective study of hormonal contraception and women's risk of HIV infection in South Africa. Int J Epidemiol. 2007;36(1):166-74.

28. Smith-McCune KK, Shiboski S, Chirenje MZ, Magure T, Tuveson J, Ma Y, et al. Type-specific cervico-vaginal human papillomavirus infection increases risk of HIV acquisition independent of other sexually transmitted infections. PLoS One. 2010;5(4):e10094-e.

29. Gallagher KE, Baisley K, Grosskurth H, Vallely A, Kapiga S, Vandepitte J, et al. The Association Between Cervical Human Papillomavirus Infection and Subsequent HIV Acquisition in Tanzanian and Ugandan Women: A Nested Case-Control Study. J Infect Dis. 2016;214(1):87-95.

30. Veldhuijzen NJ, Vyankandondera J, van de Wijgert JH. HIV acquisition is associated with prior high-risk human papillomavirus infection among high-risk women in Rwanda. AIDS. 2010;24(14):2289-92.

31. Wang C, Wright TC, Denny L, Kuhn L. Rapid rise in detection of human papillomavirus (HPV) infection soon after incident HIV infection among South African women. J Infect Dis. 2011;203(4):479-86.

32. Tanser F, Jones KG, Viljoen J, Imrie J, Grapsa E, Newell M-L. Human papillomavirus seropositivity and subsequent risk of HIV acquisition in rural South African women. Sex Transm Dis. 2013;40(7):601-6.

33. Liu G, Mugo NR, Brown ER, Mgodi NM, Chirenje ZM, Marrazzo JM, et al. Prevalent HPV infection increases the risk of HIV acquisition in African women: advancing the argument for HPV immunization. AIDS. 2021.

34. Franco EL, Villa LL, Sobrinho JP, Prado JM, Rousseau MC, Désy M, et al. Epidemiology of acquisition and clearance of cervical human papillomavirus infection in women from a high-risk area for cervical cancer. J Infect Dis. 1999;180(5):1415-23.

35. Lodi S, Phillips A, Touloumi G, Geskus R, Meyer L, Thiebaut R, et al. Time from human immunodeficiency virus seroconversion to reaching CD4+ cell count thresholds <200, <350, and <500 Cells/mm(3): assessment of need following changes in treatment guidelines. Clinical infectious diseases : an official publication of the Infectious Diseases Society of America. 2011;53(8):817-25.

36. Lyles RH, Munoz A, Yamashita TE, Bazmi H, Detels R, Rinaldo CR, et al. Natural history of human immunodeficiency virus type 1 viremia after seroconversion and proximal to AIDS in a large cohort of homosexual men. Multicenter AIDS Cohort Study. The Journal of infectious diseases. 2000;181(3):872-80.

37. Pantazis N, Morrison C, Amornkul PN, Lewden C, Salata RA, Minga A, et al. Differences in HIV natural history among African and non-African seroconverters in Europe and seroconverters in sub-Saharan Africa. PLoS One. 2012;7(3):e32369.

38. Lewden C, Gabillard D, Minga A, Ekouevi DK, Avit D, Konate I, et al. CD4-specific mortality rates among HIV-infected adults with high CD4 counts and no antiretroviral treatment in West Africa. Journal of acquired immune deficiency syndromes (1999). 2012;59(2):213-9.

39. Maduna PH, Dolan M, Kondlo L, Mabuza H, Dlamini JN, Polis M, et al. Morbidity and mortality according to latest CD4+ cell count among HIV positive individuals in South Africa who enrolled in project Phidisa. PLoS One. 2015;10(4):e0121843.

40. Adler WH, Baskar PV, Chrest FJ, Dorsey-Cooper B, Winchurch RA, Nagel JE. HIV infection and aging: mechanisms to explain the accelerated rate of progression in the older patient. Mech Ageing Dev. 1997;96(1-3):137-55.

41. Brinkhof MW, Boulle A, Weigel R, Messou E, Mathers C, Orrell C, et al. Mortality of HIV-infected patients starting antiretroviral therapy in sub-Saharan Africa: comparison with HIV-unrelated mortality. PLoS medicine. 2009;6(4):e1000066.

42. Cornell M, Johnson LF, Wood R, Tanser F, Fox MP, Prozesky H, et al. Twelve-year mortality in adults initiating antiretroviral therapy in South Africa. Journal of the International AIDS Society. 2017;20(1):21902.

43. de Coninck Z, Hussain-Alkhateeb L, Bratt G, Ekstrom AM, Gisslen M, Petzold M, et al. Non-AIDS Mortality Is Higher Among Successfully Treated People Living with HIV Compared with Matched HIV-Negative Control Persons: A 15-Year Follow-Up Cohort Study in Sweden. AIDS patient care and STDs. 2018;32(8):297-305.

44. Bouvard V, Baan R, Straif K, Grosse Y, Secretan B, El Ghissassi F, et al. A review of human carcinogens--Part B: biological agents. The Lancet Oncology. 2009;10(4):321-2.

45. Burchell AN, Richardson H, Mahmud SM, Trottier H, Tellier PP, Hanley J, et al. Modeling the sexual transmissibility of human papillomavirus infection using stochastic computer simulation and empirical data from a cohort study of young women in Montreal, Canada. Am J Epidemiol. 2006;163(6):534-43.

46. Denny L, Adewole I, Anorlu R, Dreyer G, Moodley M, Smith T, et al. Human papillomavirus prevalence and type distribution in invasive cervical cancer in sub-Saharan Africa. International journal of cancer. 2014;134(6):1389-98.

47. Dartell M, Rasch V, Kahesa C, Mwaiselage J, Ngoma T, Junge J, et al. Human papillomavirus prevalence and type distribution in 3603 HIV-positive and HIV-negative women in the general population of Tanzania: the PROTECT study. Sexually transmitted diseases. 2012;39(3):201-8.

48. Beachler DC, Jenkins G, Safaeian M, Kreimer AR, Wentzensen N. Natural Acquired Immunity Against Subsequent Genital Human Papillomavirus Infection: A Systematic Review and Meta-analysis. J Infect Dis. 2016;213(9):1444-54.

49. Johnson HC, Elfstrom KM, Edmunds WJ. Inference of type-specific HPV transmissibility, progression and clearance rates: a mathematical modelling approach. PLoS One. 2012;7(11):e49614.

50. Liu G, Sharma M, Tan N, Barnabas RV. HIV-positive women have higher risk of human papilloma virus infection, precancerous lesions, and cervical cancer. AIDS (London, England). 2018;32(6):795-808.

51. Kelly H, Weiss HA, Benavente Y, de Sanjose S, Mayaud P. Association of antiretroviral therapy with high-risk human papillomavirus, cervical intraepithelial neoplasia, and invasive cervical cancer in women living with HIV: a systematic review and meta-analysis. The lancet HIV. 2018;5(1):e45-e58.

52. Suehiro TT, Damke G, Damke E, de Azevedo Ramos PLR, de Andrade Pereira Silva M, Pelloso SM, et al. Cervical and oral human papillomavirus infection in women living with human immunodeficiency virus (HIV) and matched HIV-negative controls in Brazil. Infectious agents and cancer. 2020;15:31.

53. Rohner E, Bütikofer L, Schmidlin K, Sengayi M, Maskew M, Giddy J, et al. Cervical cancer risk in women living with HIV across four continents: A multicohort study. Int J Cancer. 2020;146(3):601-9.

54. Rohner E, Sengayi M, Goeieman B, Michelow P, Firnhaber C, Maskew M, et al. Cervical cancer risk and impact of Pap-based screening in HIV-positive women on antiretroviral therapy in Johannesburg, South Africa. International journal of cancer. 2017;141(3):488-96.

55. Sankaranarayanan R, Swaminathan R, Brenner H, Chen K, Chia KS, Chen JG, et al. Cancer survival in Africa, Asia, and Central America: a population-based study. Lancet Oncol. 2010;11(2):165-73.

56. Dryden-Peterson S, Bvochora-Nsingo M, Suneja G, Efstathiou JA, Grover S, Chiyapo S, et al. HIV Infection and Survival Among Women With Cervical Cancer. Journal of clinical oncology : official journal of the American Society of Clinical Oncology. 2016;34(31):3749-57.

57. Liu G, Sharma M, Tan N, Barnabas RV. HIV-positive women have higher risk of human papilloma virus infection, precancerous lesions, and cervical cancer. AIDS. 2018;32(6):795-808.

58. Campos NG, Burger EA, Sy S, Sharma M, Schiffman M, Rodriguez AC, et al. An updated natural history model of cervical cancer: derivation of model parameters. Am J Epidemiol. 2014;180(5):545-55.

59. Rosillon D, Baril L, Del Rosario-Raymundo MR, Wheeler CM, Skinner SR, Garland SM, et al. Risk of newly detected infections and cervical abnormalities in adult women seropositive or seronegative for naturally acquired HPV-16/18 antibodies. Cancer Med. 2019;8(10):4938-53.

60. Lilian RR, Rees K, Mabitsi M, McIntyre JA, Struthers HE, Peters RPH. Baseline CD4 and mortality trends in the South African human immunodeficiency virus programme: Analysis of routine data. South Afr J HIV Med. 2019;20(1):963.

61. Rodger AJ, Cambiano V, Bruun T, Vernazza P, Collins S, van Lunzen J, et al. Sexual Activity Without Condoms and Risk of HIV Transmission in Serodifferent Couples When the HIV-Positive Partner Is Using Suppressive Antiretroviral Therapy. Jama. 2016;316(2):171-81.

62. Eisinger RW, Dieffenbach CW, Fauci AS. HIV Viral Load and Transmissibility of HIV Infection: Undetectable Equals Untransmittable. Jama. 2019;321(5):451-2.

63. Kenya Ministry of Health National AIDS & STI Control Program. The Guidelines on Use of Antiretroviral Drugs for Treating and Preventing HIV Infection in Kenya. Nairobi: Kenya Ministry of Health 2018 August.

64. Kenya Ministry of Health. Kenya AIDS Response Progress Report 2016. Available from: <http://nacc.or.ke/wp-content/uploads/2016/11/Kenya-AIDS-Progress-Report_web.pdf>.

65. Kenya Ministry of Health National AIDS & STI Control Program. Kenya AIDS Response Progress Report, Progress Towards Zero. Nairobi, Kenya: Kenya Ministry of Health; 2014.

66. Kenya Ministry of Health National AIDS & STI Control Program. Kenya Population-based HIV Impact Assessment (KENPHIA) 2018. Nairobi, Kenya: National AIDS & STI Control Program; 2020.

67. Karcher H, Omondi A, Odera J, Kunz A, Harms G. Risk factors for treatment denial and loss to follow-up in an antiretroviral treatment cohort in Kenya. Tropical medicine & international health : TM & IH. 2007;12(5):687-94.

68. Zachariah R, Van Engelgem I, Massaquoi M, Kocholla L, Manzi M, Suleh A, et al. Payment for antiretroviral drugs is associated with a higher rate of patients lost to follow-up than those offered free-of-charge therapy in Nairobi, Kenya. Transactions of the Royal Society of Tropical Medicine and Hygiene. 2008;102(3):288-93.

69. Kenya Ministry of Health National AIDS & STI Control Program. AIDS Response Progress Report Nairobi, Kenya: Kenya Ministry of Health 2016.

70. Waithaka M, Bessinger R. Sexual Behavior and Condom Use in the Context of HIV Prevention in Kenya. Population Services International; 2001.

71. Cherutich P, Brentlinger P, Nduati R, Kiarie JN, Farquhar C. Condom use among sexually active Kenyan female adolescents at risk for HIV-1 infection. AIDS and behavior. 2008;12(6):923-9.

72. Weller S, Davis K. Condom effectiveness in reducing heterosexual HIV transmission. Cochrane Database Syst Rev. 2002(1):CD003255.

73. Manhart LE, Koutsky LA. Do condoms prevent genital HPV infection, external genital warts, or cervical neoplasia? A meta-analysis. Sex Transm Dis. 2002;29(11):725-35.

74. Ho GY, Studentsov YY, Bierman R, Burk RD. Natural history of human papillomavirus type 16 virus-like particle antibodies in young women. Cancer epidemiology, biomarkers & prevention : a publication of the American Association for Cancer Research, cosponsored by the American Society of Preventive Oncology. 2004;13(1):110-6.

75. Moscicki AB, Hills N, Shiboski S, Powell K, Jay N, Hanson E, et al. Risks for incident human papillomavirus infection and low-grade squamous intraepithelial lesion development in young females. JAMA. 2001;285(23):2995-3002.

76. Sanchez-Aleman MA, Uribe-Salas FJ, Lazcano-Ponce EC, Conde-Glez CJ. Human papillomavirus incidence and risk factors among Mexican female college students. Sex Transm Dis. 2011;38(4):275-8.

77. Siegfried N, Muller M, Deeks JJ, Volmink J. Male circumcision for prevention of heterosexual acquisition of HIV in men. Cochrane Database of Systematic Reviews. 2009(2).

78. Albero G, Castellsagué X, Lin H-Y, Fulp W, Villa LL, Lazcano-Ponce E, et al. Male circumcision and the incidence and clearance of genital human papillomavirus (HPV) infection in men: the HPV Infection in men (HIM) cohort study. BMC Infect Dis. 2014;14:75-.

79. Albero G, Villa LL, Lazcano-Ponce E, Fulp W, Papenfuss MR, Nyitray AG, et al. Male circumcision and prevalence of genital human papillomavirus infection in men: a multinational study. BMC Infect Dis. 2013;13:18-.

80. Vanbuskirk K, Winer RL, Hughes JP, Feng Q, Arima Y, Lee S-K, et al. Circumcision and acquisition of human papillomavirus infection in young men. Sex Transm Dis. 2011;38(11):1074-81.

81. Tobian AAR, Kong X, Gravitt PE, Eaton KP, Kigozi G, Serwadda D, et al. Male circumcision and anatomic sites of penile high-risk human papillomavirus in Rakai, Uganda. Int J Cancer. 2011;129(12):2970-5.

82. Tobian AA, Kong X, Wawer MJ, Kigozi G, Gravitt PE, Serwadda D, et al. Circumcision of HIV-infected men and transmission of human papillomavirus to female partners: analyses of data from a randomised trial in Rakai, Uganda. The Lancet Infectious diseases. 2011;11(8):604-12.

83. Weiss HA, Hankins CA, Dickson K. Male circumcision and risk of HIV infection in women: a systematic review and meta-analysis. The Lancet Infectious diseases. 2009;9(11):669-77.

84. Lei JH, Liu LR, Wei Q, Yan SB, Yang L, Song TR, et al. Circumcision Status and Risk of HIV Acquisition during Heterosexual Intercourse for Both Males and Females: A Meta-Analysis. PLoS One. 2015;10(5):e0125436.

85. Wawer MJ, Tobian AAR, Kigozi G, Kong X, Gravitt PE, Serwadda D, et al. Effect of circumcision of HIV-negative men on transmission of human papillomavirus to HIV-negative women: a randomised trial in Rakai, Uganda. Lancet (London, England). 2011;377(9761):209-18.

86. Shaffer DN, Bautista CT, Sateren WB, Sawe FK, Kiplangat SC, Miruka AO, et al. The protective effect of circumcision on HIV incidence in rural low-risk men circumcised predominantly by traditional circumcisers in Kenya: two-year follow-up of the Kericho HIV Cohort Study. J Acquir Immune Defic Syndr. 2007;45(4):371-9.

87. Central Bureau of Statistics CBSK, Ministry of Health MOHK, Macro ORC. Kenya Demographic and Health Survey 2003. Calverton, Maryland, USA: CBS, MOH, and ORC Macro; 2004.

88. Fleming PJ, Doshi M, Harper GW, Otieno F, Bailey RC. Integration of voluntary male medical circumcision for HIV prevention into norms of masculinity: findings from Kisumu, Kenya. Culture, health & sexuality. 2020:1-13.

89. Kenya National Bureau of Statistics - KNBS, National AIDS Control Council/Kenya, National AIDS/STD Control Programme/Kenya, Health MoP, Sanitation/Kenya, Kenya Medical Research Institute. Kenya Demographic and Health Survey 2008-09. Calverton, Maryland, USA: KNBS and ICF Macro; 2010.

90. Kenya Ministry of Health and Sanitation. National Guidelines for Prevention and Management of Cervical, Breast and Prostate Cancers. Nairobi, Kenya: Kenya Ministry of Health and Sanitation; 2012.

91. Gakidou E, Nordhagen S, Obermeyer Z. Coverage of cervical cancer screening in 57 countries: low average levels and large inequalities. PLoS Med. 2008;5(6):e132-e.

92. Chung MH, McKenzie KP, De Vuyst H, Richardson BA, Rana F, Pamnani R, et al. Comparing Papanicolau smear, visual inspection with acetic acid and human papillomavirus cervical cancer screening methods among HIV-positive women by immune status and antiretroviral therapy. AIDS. 2013;27(18):2909-19.

93. Khozaim K, Orang'o E, Christoffersen-Deb A, Itsura P, Oguda J, Muliro H, et al. Successes and challenges of establishing a cervical cancer screening and treatment program in western Kenya. International journal of gynaecology and obstetrics: the official organ of the International Federation of Gynaecology and Obstetrics. 2014;124(1):12-8.

94. Greene SA, De Vuyst H, John-Stewart GC, Richardson BA, McGrath CJ, Marson KG, et al. Effect of Cryotherapy vs Loop Electrosurgical Excision Procedure on Cervical Disease Recurrence Among Women With HIV and High-Grade Cervical Lesions in Kenya: A Randomized Clinical Trial. JAMA. 2019;322(16):1570-9.

95. Kuhn L, Wang C, Tsai WY, Wright TC, Denny L. Efficacy of human papillomavirus-based screen-and-treat for cervical cancer prevention among HIV-infected women. AIDS. 2010;24(16):2553-61.

96. Hoffman SR, Le T, Lockhart A, Sanusi A, Dal Santo L, Davis M, et al. Patterns of persistent HPV infection after treatment for cervical intraepithelial neoplasia (CIN): A systematic review. International journal of cancer. 2017;141(1):8-23.

97. Ferlay J, Soerjomataram I, Dikshit R, Eser S, Mathers C, Rebelo M, et al. Cancer incidence and mortality worldwide: sources, methods and major patterns in GLOBOCAN 2012. Int J Cancer. 2015;136(5):E359-86.

98. Jedy-Agba E, Joko WY, Liu B, Buziba NG, Borok M, Korir A, et al. Trends in cervical cancer incidence in sub-Saharan Africa. British Journal of Cancer. 2020;123(1):148-54.

99. Kenya Ministry of Health and Sanitation. 2007 Kenya AIDS Indicator Survey (KAIS). Nairobi, Kenya: Kenya Ministry of Health and Sanitation,; 2009.

100. Kenya Ministry of Health. 2012 Kenya AIDS Indicator Survey (KAIS). Nairobi, Kenya: Ministry of Health 2014.

101. Luchters SMF, Broeck DV, Chersich MF, Nel A, Delva W, Mandaliya K, et al. Association of HIV infection with distribution and viral load of HPV types in Kenya: a survey with 820 female sex workers. BMC Infect Dis. 2010;10(1):18.

102. Yamada R, Sasagawa T, Kirumbi LW, Kingoro A, Karanja DK, Kiptoo M, et al. Human papillomavirus infection and cervical abnormalities in Nairobi, Kenya, an area with a high prevalence of human immunodeficiency virus infection. J Med Virol. 2008;80(5):847-55.

103. Bruni L AG, Serrano B, Mena M, Gómez D, Muñoz J, Bosch FX, de Sanjosé S. Human Papillomavirus and Related Diseases in South Africa: Summary Report 17 June 2019.

104. Van Aardt MC, Dreyer G, Richter KL, Becker P. Human papillomavirus-type distribution in South African women without cytological abnormalities: a peri-urban study. Southern African Journal of Gynaecological Oncology. 2013;5(sup1):S21-S7.

105. Clifford GM, Rana RK, Franceschi S, Smith JS, Gough G, Pimenta JM. Human papillomavirus genotype distribution in low-grade cervical lesions: comparison by geographic region and with cervical cancer. Cancer epidemiology, biomarkers & prevention : a publication of the American Association for Cancer Research, cosponsored by the American Society of Preventive Oncology. 2005;14(5):1157-64.

106. Van Aardt MC, Dreyer G, Snyman LC, Richter KL, Becker P, Mojaki SM. Oncogenic and incidental HPV types associated with histologically confirmed cervical intraepithelial neoplasia in HIV-positive and HIV-negative South African women. S Afr Med J. 2016;106(6).

107. de Sanjose S, Quint WG, Alemany L, Geraets DT, Klaustermeier JE, Lloveras B, et al. Human papillomavirus genotype attribution in invasive cervical cancer: a retrospective cross-sectional worldwide study. Lancet Oncol. 2010;11(11):1048-56.

108. van Aardt MC, Dreyer G, Pienaar HF, Karlsen F, Hovland S, Richter KL, et al. Unique human papillomavirus-type distribution in South African women with invasive cervical cancer and the effect of human immunodeficiency virus infection. International journal of gynecological cancer : official journal of the International Gynecological Cancer Society. 2015;25(5):919-25.

109. Sung H, Ferlay J, Siegel RL, Laversanne M, Soerjomataram I, Jemal A, et al. Global cancer statistics 2020: GLOBOCAN estimates of incidence and mortality worldwide for 36 cancers in 185 countries. CA: a cancer journal for clinicians. 2021.

110. Canfell K, Kim JJ, Kulasingam S, et al. HPV-FRAME: A consensus statement and quality framework for modelled evaluations of HPV-related cancer control. Papillomavirus Res. 2019;8:100184.
